# β-Catenin Stabilization in Skin Fibroblasts Causes Fibrotic Lesions by Preventing Adipocyte Differentiation of the Reticular Dermis

**DOI:** 10.1016/j.jid.2016.01.036

**Published:** 2016-06

**Authors:** Maria Mastrogiannaki, Beate M. Lichtenberger, Andreas Reimer, Charlotte A. Collins, Ryan R. Driskell, Fiona M. Watt

**Affiliations:** 1 Centre for Stem Cells and Regenerative Medicine, King’s College London, Guy’s Hospital, Great Maze Pond, London SE1 9RT, UK; 2 Wellcome Trust Centre for Stem Cell Research, University of Cambridge, Tennis Court Road, Cambridge CB2 1QR, UK

**Keywords:** DAPI, 4′, 6-diamidino-2-phenylindole, DP, dermal papilla, ECM, extracellular matrix, EGFP, enhanced green fluorescent protein, HF, hair follicle, PDGFRα, platelet-derived growth factor receptor α, Sca1, stem cell antigen 1

## Abstract

The Wnt/β-catenin pathway plays a central role in epidermal homeostasis and regeneration, but how it affects fibroblast fate decisions is unknown. We investigated the effect of targeted β-catenin stabilization in dermal fibroblasts. Comparative gene expression profiling of stem cell antigen 1^-^ (Sca1^-^) and Sca1^+^ neonatal fibroblasts from upper and lower dermis, respectively, confirmed that Sca1^+^ cells had a preadipocyte signature and showed differential expression of Wnt/β-catenin–associated genes. By targeting all fibroblasts or selectively targeting Dlk1^+^ lower dermal fibroblasts, we found that β-catenin stabilization between developmental stages E16.5 and P2 resulted in a reduction in the dermal adipocyte layer with a corresponding increase in dermal fibrosis and an altered hair cycle. The fibrotic phenotype correlated with a reduction in the potential of Sca1^+^ fibroblasts to undergo adipogenic differentiation ex vivo. Our findings indicate that Wnt/β-catenin signaling controls adipogenic cell fate within the lower dermis, which potentially contributes to the pathogenesis of fibrotic skin diseases.

## Introduction

The epidermis is maintained by distinct subpopulations of stem cells whose fate is regulated by intrinsic mechanisms and external signals from the niche (Kretzschmar and Watt, 2014, Lim and Nusse, 2013). Signaling between epidermal stem cells and cells in the underlying dermis is reciprocal (Millar, 2002, Sennett and Rendl, 2012). Such interactions can occur at close range—for example, via extracellular matrix (ECM) deposition (Fujiwara et al., 2011)—and by long-range signaling mediated by epidermal secreted factors (Collins et al., 2011, Donati et al., 2014). Furthermore, factors secreted by proliferating adipocyte progenitors (Festa et al., 2011) and differentiated adipocytes (Plikus and Chuong, 2014, Plikus et al., 2008) regulate the hair growth cycle.

The dermis contains a variety of subpopulations of mesenchymal cells with different locations and functions (Driskell and Watt, 2015). At the E12.5 stage of embryonic development, mouse dermal cells are capable of differentiating into all the different fibroblast types present in postnatal skin. However, at about E16.5, the dermal mesenchyme undergoes commitment to two different lineages. In late embryonic and neonatal skin, the upper dermal lineage gives rise to papillary fibroblasts, the cells of the arrector pili muscle (responsible for piloerection), the dermal sheath, and the dermal papilla (DP). The lower dermal lineage gives rise to the reticular fibroblasts, which deposit most of the skin fibrillar collagen, preadipocytes and mature adipocytes (Driskell et al., 2013). The different fibroblast lineages are functionally significant, because the upper papillary lineage is required for new hair follicle (HF) formation, and the lower (or reticular) lineage is responsible for the first wave of dermal repair after wounding (Driskell et al., 2013). However, the molecular mechanisms that determine fibroblast fate decisions are largely uncharacterized (Driskell et al., 2009).

Epidermal Wnt/β-catenin signaling causes profound changes in the underlying dermis, leading to expansion of both the upper and lower dermal lineages, de novo formation of DPs, and an increase in adipocyte differentiation (Collins et al., 2011, Donati et al., 2014, Lichtenberger et al., 2016). In addition, there is compelling evidence that Wnt/β-catenin signaling in fibroblasts regulates the composition of the dermis. β-Catenin is necessary and sufficient to specify dermal fate in different body regions during mouse embryonic development (Atit et al., 2006, Ohtola et al., 2008). Wnt/β-catenin signaling has a well-characterized inhibitory effect on adipogenic differentiation (Gesta et al., 2007, Kennell and MacDougald, 2005, Longo et al., 2004) and is required in the DP to promote HF formation and control DP activity and size (Enshell-Seijffers et al., 2012, Kaushal et al., 2015, Tsai et al., 2014). Furthermore, it was shown recently that expression of stabilized β-catenin in the fibroblasts of mouse ventral dermis at E16.5 results in progressive skin fibrosis, with thickened collagen fibers and altered collagen fibril morphology (Hamburg-Shields et al., 2015).

In this study, we performed comparative gene expression profiling of distinct populations of neonatal fibroblasts, which showed differential expression of Wnt pathway genes. Using a conditional targeting approach, we examined the effects of β-catenin stabilization in all fibroblasts or selectively in reticular fibroblasts. Our findings indicate a key role of Wnt/β-catenin signaling in regulating the differentiation of reticular fibroblasts into adipocytes.

## Results

### Differential expression of adipocyte genes in upper and lower dermal fibroblasts

**Figure 1:**
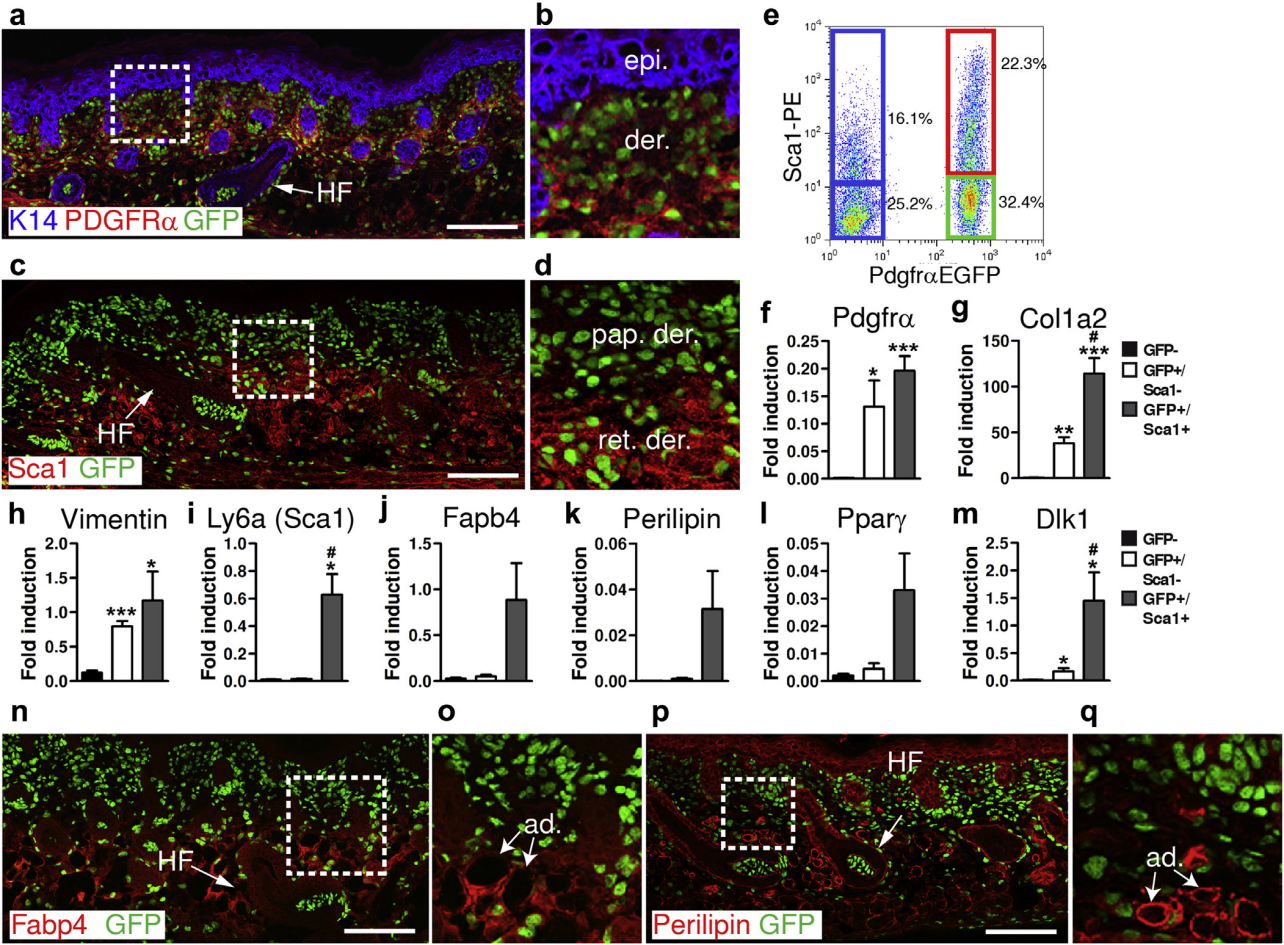
**Localization and isolation of Sca1^+^ and Sca1^-^ dermal fibroblasts.(a–d, n–q)** Sections of P2 back skin from PdgfrαEGFP mice showing expression of nuclear EGFP and immunostaining for **(a, b)** K14 and PDGFRα, **(c, d)** Sca1, **(n, o)** Fabp4, and **(p, q)** Perilipin. **(b, d, o, q)** are enlargements of selected regions of **(a, c, n, p)**, respectively. Scale bars = 150 μm. **(e)** Flow cytometry plot showing gating of different dermal subpopulations on the basis of PdgfrαEGFP and Sca1 expression. **(f–m)** Quantitative real-time PCR analysis of mRNA levels in sorted cell populations: PdgfrαEGFP^-^/Sca1^-^, PdgfrαEGFP^+^/Sca1^-^ and PdgfrαEGFP^+^/Sca1^+^. Genes were normalized to the *Gapdh* gene. Error bars represent standard error of the mean of replicates from four mice. ^∗^*P* ≤ 0.05, ^∗∗^*P* ≤ 0.005, ^∗∗∗^*P* ≤ 0.0005 compared with GFP^-^ cells; ^#^p ≤ 0.05 compared with EGFP^+^/Sca1^-^ cells. ad, adipocyte; epi, epidermis; der, dermis; EGFP, enhanced green fluorescent protein; GFP, green fluorescent protein; HF, hair follicle; pap, papillary; PDGFRα, platelet-derived growth factor receptor α; ret, reticular.

As previously reported (Collins et al., 2011), in PdgfrαEGFP mice (Hamilton et al., 2003) enhanced green fluorescent protein (EGFP) expressed under the control of the platelet-derived growth factor receptor α (PDGFRα) promoter is detected in nuclei of all dorsal skin fibroblasts (Figure 1a–d). In neonatal (P2) skin, stem cell antigen 1 (Sca1) is absent from the upper (papillary) dermis but is expressed by reticular fibroblasts, preadipocytes, and skin adipocytes in the lower dermis (Donati et al., 2014, Driskell et al., 2013, Festa et al., 2011). Immunolabeling confirmed that most reticular fibroblasts express Sca1 at P2 (Figure 1c and d). We have previously shown that fibroblasts that express the protein Delta homolog 1 (Dlk1) at E16.5 give rise to the Sca1^+^ fibroblasts and mature adipocytes present in P2 and adult skin, although Dlk1 is no longer expressed in adult skin (Driskell et al., 2013, Lichtenberger et al., 2016). To compare fibroblasts from the upper and lower dermis, we sorted EGFP^+^ cells from P2 dorsal dermis and fractionated the cells further on the basis of Sca1 surface marker expression (Figure 1e). We also sorted EGFP^-^/Sca1^-^ cells as a control.

Quantitative real-time PCR (QPCR) analysis of mRNA isolated from the different dermal subpopulations showed that mRNAs corresponding to *Pdgfra* and the fibroblast marker genes *Col1a2* and *Vimentin* were highly enriched in both Pdgfrα^+^ subpopulations relative to Pdgfrα^-^ cells (Figure 1f–h), whereas mRNA corresponding to *Ly6a* (Sca1) was highly enriched in the Pdgfrα^+^/Sca1^+^ fraction (Figure 1i), confirming the relative purity of the sorted cell populations. The adipocyte/preadipocyte marker genes *Fabp4*, *Perilipin*, *Pparg*, and *Dlk1* were also enriched in Pdgfrα^+^/Sca1^+^ cells, consistent with previous reports (Driskell et al., 2013, Festa et al., 2011) (Figure 1j–m). The QPCR results were confirmed by immunofluorescence labeling of P2 dorsal skin with antibodies to Fabp4 and Perilipin (Figure 1n–q).

### Differential expression of Wnt pathway genes in upper and lower dermal fibroblasts

**Figure 2:**
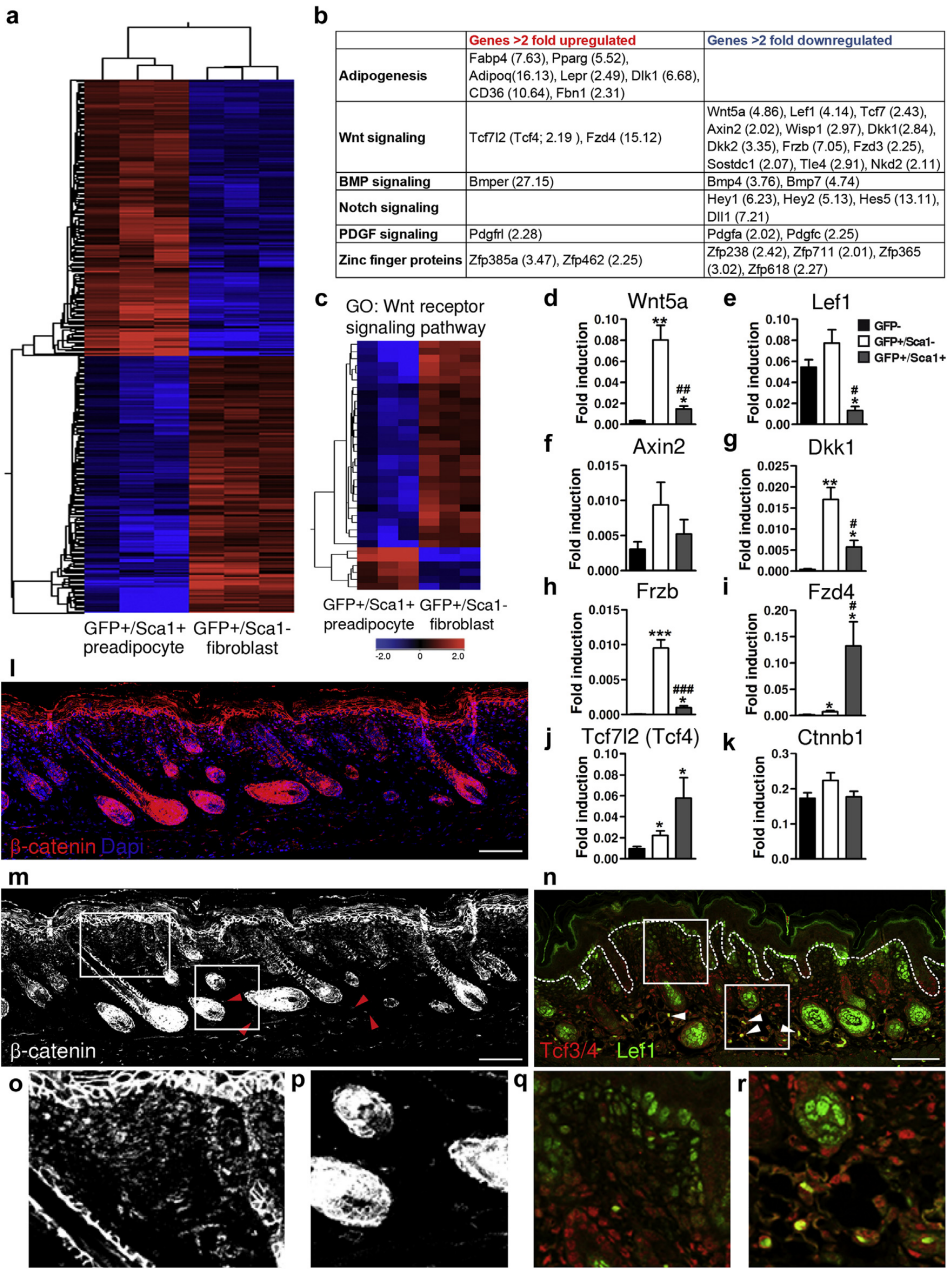
**Distinct transcriptional signature of PdgfrαEGFP^+^/Sca1^+^ dermal cells.(a)** Heat map showing hierarchical clustering (based on entities and samples) of all differentially regulated genes (*P* < 0.05, change >2-fold) between PdgfrαEGFP^+^/Sca1^+^ and PdgfrαEGFP^+^/Sca1^-^ fibroblasts. **(b)** Selected genes up-regulated or down-regulated in Sca1^+^ cells. Values in parentheses represent fold change of each gene. **(c)** Heat map showing hierarchical clustering (based on entities) of all regulated genes in the Gene Ontology term “Wnt receptor signaling pathway”. **(d–k)** Quantitative real-time PCR analysis of mRNA levels in sorted cell populations, normalized to *Gapdh* gene expression. Error bars represent standard error of the mean of replicates from four mice. ^∗^*P* ≤ 0.05, ^∗∗^*P* ≤ 0.005, ^∗∗∗^*P* ≤ 0.0005 compared with GFP^-^ cells; ^#^*P* ≤ 0.05 compared with GFP^+^/Sca1^-^ cells. **(l, m)** Immunofluorescent staining of neonatal skin with an antibody detecting β-catenin. Red arrowheads show β-catenin^+^ fibroblasts in the reticular dermis. 4′, 6-diamidino-2-phenylindole labels nuclei. Scale bar = 200 μm. **(n)** Section of P1 back skin immunostained for Tcf3/4 (red) and Lef1 (green). White arrowheads indicate double-labeled cells. Dashed lines demarcate epidermal-dermal boundary. Scale bar = 100 μm. **(o–r)** Higher-magnification images of the boxed areas in **(m, n)**, showing upper **(o, q)** and lower **(p, r)** dermis. BMP, bone morphogenic protein; DAPI, 4′, 6-diamidino-2-phenylindole; GO, Gene Ontology; PDGF, platelet-derived growth factor.

To explore the differences between Pdgfrα^+^/Sca1^+^ and Pdgfrα^+^/Sca1^-^ fibroblasts, we carried out gene expression profiling using RNA from flow-sorted cells. We found that 1,457 entities were regulated by more than 2-fold (*t* test, *P* < 0.05) (Figure 2a; see Supplementary Table S1 online), showing that global differences in gene expression distinguish the two fibroblast subpopulations. In addition to differential expression of adipogenic genes, there was differential expression of genes encoding zinc finger proteins (Gupta et al., 2012) and regulators of the Wnt, BMP, Notch, and PDGF signaling pathways (Figure 2b and c).

Because Wnt/β-catenin signaling is known to regulate dermal development, the differential expression of genes associated with this pathway was of particular interest (Figure 2c). Several Wnt/β-catenin pathway genes were differentially regulated in Pdgfrα^+^/Sca1^+^ and Pdgfrα^+^/Sca1^-^ fibroblasts, which we confirmed by QPCR in independent biological samples (Figure 2d–j). Pdgfrα^+^/Sca1^+^ fibroblasts expressed significantly lower levels of *Wnt5a* ligand, the Wnt receptor *Frzb*, and the Wnt effector *Lef1*, as well as several other Wnt pathway genes including *Axin2* and *Dkk1* (Figure 2d–h; see also Driskell et al., 2013). However, Sca1^+^ cells expressed significantly higher levels of the Wnt receptor *Fzd4* and the Wnt effector *Tcf7l2* (Figure 2i and j). Tcf7l2, commonly known as Tcf4, is expressed in human adipose tissue, and gene variants are associated with susceptibility to Type 2 diabetes and inability to lose weight after lifestyle interventions (Cauchi et al., 2006, Haupt et al., 2010). There was no significant difference in β-catenin mRNA levels in Pdgfrα^+^/Sca1^-^ and Pdgfrα^+^/Sca1^+^ fibroblasts at P2 (Figure 2k). However, immunostaining showed differential protein expression of β-catenin in the upper and lower dermis of neonatal skin, with high levels of nuclear β-catenin in papillary fibroblasts and only few nuclear β-catenin–positive cells within the adipose tissue (Figure 2l–p).

Consistent with the microarray and QPCR data, immunostaining of neonatal skin with antibodies recognizing Tcf3/4 and Lef1 showed that Tcf3/4 localized to the lower reticular dermis (Pdgfrα^+^/Sca1^+^), whereas Lef1 stained the upper papillary dermis (Pdgfrα^+^/Sca1^-^) (Figure 2n, q, and r). However, there were some scattered cells in the lower dermis that coexpressed Tcf3/4 and Lef1 (Figure 2n, white arrowheads).

We conclude that neonatal dermis is compartmentalized such that Wnt/β-catenin signaling pathway components are differentially expressed in Sca1^+^ and Sca1^-^ fibroblasts.

### Constitutive β-catenin stabilization in postnatal skin fibroblasts reduces the adipocyte layer and disturbs the hair growth cycle

**Figure 3:**
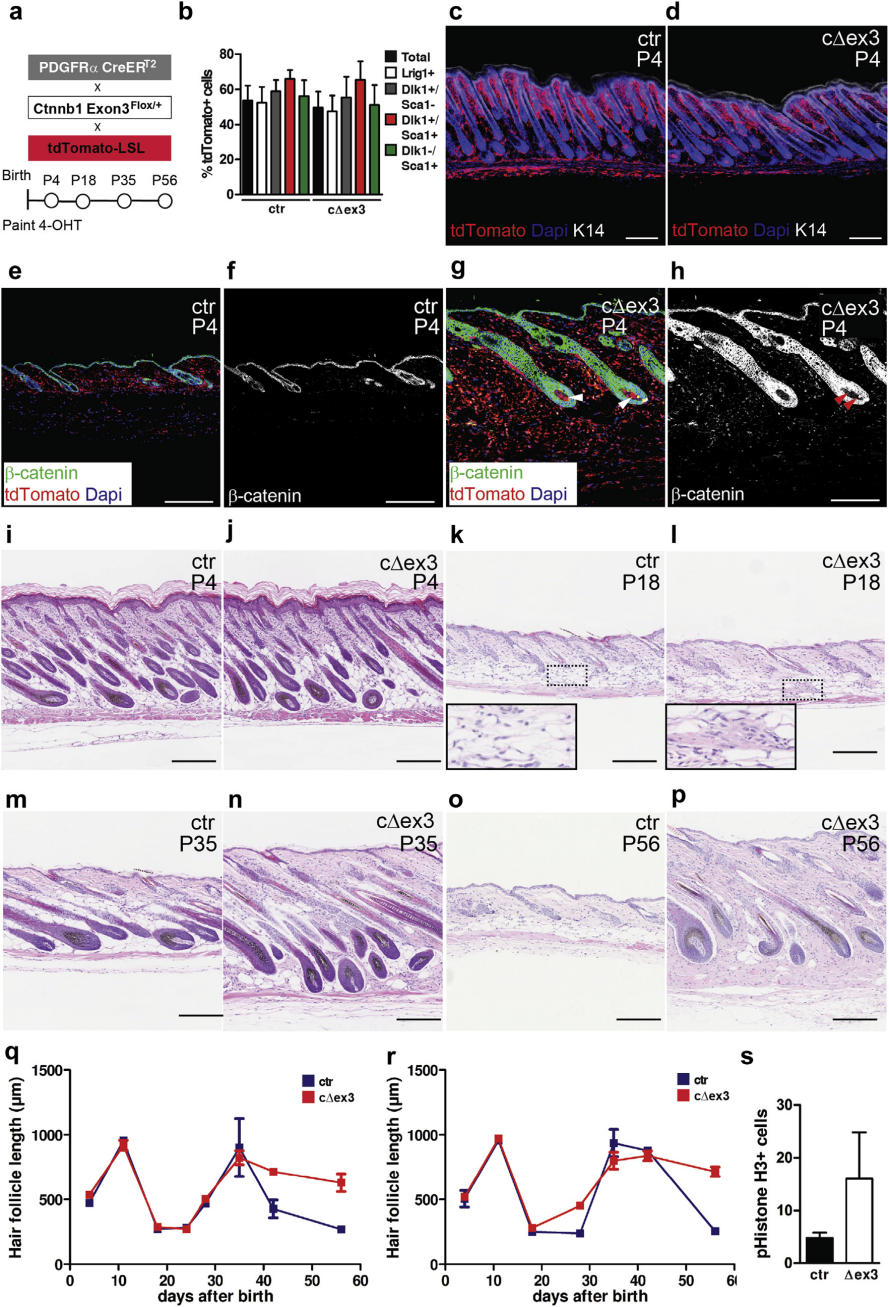
**β-Catenin stabilization in dermal fibroblasts via PdgfrαCreER^T2^.(a)** Schematic illustration of experimental strategy. **(b)** Recombination efficiency of PDGFRαCreER^T2^. Bar graph showing percentage of tdTomato^+^ cells within different fibroblast subsets after gating for ITGA6^-^/LIN^-^/CD31^-^ cells 2 days after 4-OHT–mediated recombination. Data are reported as mean ± standard error of the mean of triplicate samples in a representative experiment (n = 2–3 independent experiments). **(c, d)** tdTomato expression in 4-OHT–treated dorsal skin from **(c)** PDGFRαCreER^T2^ × tdTomato-LSL × Ctnnb1 Exon3^+/+^ (control) and **(d)** PDGFRαCreER^T2^ × tdTomato-LSL × Ctnnb1 Exon3^Flox/+^ (cΔex3) mice at P4. **(e–h)** Strong β-catenin staining is detected in tdTomato^+^ fibroblasts of mice with activated Wnt/β-catenin signaling. Note that the PDGFRαCreER^T2^ transgene is also active in cells of the dermal papilla (white arrowheads in **g**). Red arrow heads in **(h)** depict β-catenin^+^ cells in the dermal papilla. **(i–p)** Paraffin sections of back skin of control and mutant (cΔex3) littermates stained for hematoxylin and eosin at **(i, j)** P4, **(k, l)** first telogen (P18), **(m, n)** anagen (P35), and **(o, p)** second telogen (P56). Boxed areas in **(k, l)** are shown as higher-magnification inserts. Scale bars = 200 μm. **(q, r)** Hair follicle length measured in **(q)** male and **(r)** female cΔex3 and control mice at P4, P11, P18, P24, P28, P35, P42, and P56 (n = 2–8). Data points are reported as mean ± standard error of the mean. **(s)** Quantification of proliferating, phosphohistone H3^+^ cells in the outer root sheath of hair follicles in immunostained skin sections (n = 3 biological samples; ≥5 hair follicles per biological sample were scored). 4-OHT, 4-hydroxy-tamoxifen; ctr, control; Dapi, 4′, 6-diamidino-2-phenylindole; K14, keratin 14.

Given the inhibitory effect of Wnt/β-catenin signaling on adipogenic differentiation (Gesta et al., 2007, Kennell and MacDougald, 2005, Longo et al., 2004), we speculated that activating the pathway in postnatal skin fibroblasts would change the composition of the dermis by altering neonatal fibroblast lineages or differentiation. To determine the effect of active Wnt/β-catenin signaling in all neonatal fibroblasts, we produced crosses between PdgfrαCreER^T2^ (Rivers et al., 2008) and Ctnnb1 Exon3^Flox/+^ (Harada et al., 1999) mouse strains. Recombination of Ctnnb1 Exon3^Flox/+^ (referred as cΔex3) produces a variant of β-catenin that is resistant to phosphorylation by glycogen synthase kinase 3β and degradation by the adenomatous polyposis complex. Littermates were treated topically with 4-hydroxy-tamoxifen (4-OHT) on the day of birth and analyzed at different time points thereafter (Figure 3a).

By generating triple transgenics through crossing Pdgfrα-CreER^T2^, Rosa-CAG-LSL-tdTomato (tdTomato-LSL), and Ctnnb1 Exon3^Flox/+^ mice, we could show equal recombination efficiency whether or not β-catenin was stabilized. Fifty percent to 60% of all Pdgfrα^+^ fibroblasts were tdTomato^+^ at P4 in control and mutant (cΔex3) littermates (Figure 3b; see Supplementary Figure S1a online). tdTomato^+^ cells isolated from back skin at P4 did not express α6 integrin (a marker of epidermal keratinocytes), CD31 (endothelial cell marker), CD45 or other markers of hematopoietic lineages (see Supplementary Figure S1a, and data not shown).

The recombination efficiency was similar in different fibroblast subpopulations (Figure 3b). Lrig1^+^/Sca1^-^ cells are resident in the papillary dermis; Dlk1^+^/Sca1^-^ cells are a subpopulation of P2 reticular fibroblasts; Sca1^+^ cells, which can be Dlk1^+^ or Dlk1^-^, are found in the lower dermis and subdermal fat layer (Driskell et al., 2013). Analysis of skin sections confirmed that tdTomato^+^ cells were present in all dermal layers (Figure 3c and d).

A high abundance of nuclear β-catenin was detected in tdTomato^+^ fibroblasts of cΔex3 skin at P4 (see Supplementary Figure S1c–f) and was even more pronounced at P56 (Figure 3e–h). Furthermore, QPCR showed increased (albeit not significant) expression levels of known Wnt/β-catenin target genes such as *Nov*, *Sp5*, and *Gpr165* (Hamburg-Shields et al., 2015) in flow-sorted tdTomato^+^ fibroblasts isolated from mutant skin compared with control skin (see Supplementary Figure S1g) at P4. Interestingly, at P56 expression levels of Nov were similar in tdTomato^+^/Sca1^-^ fibroblasts in wild-type and mutant dermis but significantly up-regulated in tdTomato^+^/Sca1^+^ fibroblasts, thereby reaching similar levels between papillary and reticular fibroblasts (see Supplementary Figure S1h).

No differences in skin morphology were observed between mutant and control mice at P4 (Figure 3i and j). However, at P18 we observed fibroblast-rich regions within the adipocyte layer of cΔex3 mice (Figure 3k and l). At P35 these fibrotic regions were prominent (Figure 3m and n), and at P56 the adipocyte layer had been largely replaced by fibrotic dermis (Figure 3o and p).

To assess whether Wnt/β-catenin signaling in fibroblasts affects the hair cycle, we classified different stages of HFs according to their morphology (Muller-Rover et al., 2001, Paus et al., 1999) and also examined their length. For each time point we examined at least 8 HFs per skin sample and skin biopsies from 2–8 different mice of the same genotype. We examined skin at P4 and P11 (n = 3–4), during the growth phase of HF morphogenesis; at P18 (n = 3–5), P24 (n = 2–4), and P28 (n = 2), during the first postnatal telogen; at P35 (n = 6), during anagen; and at P42 (n = 4–8) and P56 (n = 3–6), when the follicles are entering catagen and the second telogen, respectively. Regardless of genotype and age, all HFs were synchronized, with the exception of P35 cΔex3 mice, when two of eight mice were still in telogen and the others were in anagen.

HF morphogenesis occurred normally in cΔex3 mice (day 4 and 10; Figure 3q and r), as did the first telogen (day 18) and first anagen (day 35; Figure 3q and r). However, from day 42 the hair cycle was disturbed in both male (Figure 3q) and female (Figure 3r) cΔex3 mice, and cΔex3 follicles remained in anagen, as assessed both by morphology and length, when wild-type follicles were entering telogen (Figure 3o–r). Based on the observation that in two of eight mutant mice the HFs were still in telogen at P35, which were not scored in (Figure 3m and n), we speculate that there may be an extended growth phase before the second catagen. This is supported by the observation that there was an increased number of proliferating phosphohistone H3^+^ cells in the outer root sheath of HFs in mutant mice (Figure 3s). Nevertheless, the HFs in mutant skin eventually entered catagen, and all HFs were in telogen at the age of 3 months (n = 4; see Supplementary Figure S1i and j). Thus, our findings indicate that fibroblast-specific β-catenin stabilization perturbs the hair cycle and extends anagen.

### Constitutive β-catenin activation results in dermal fibrosis

**Figure 4:**
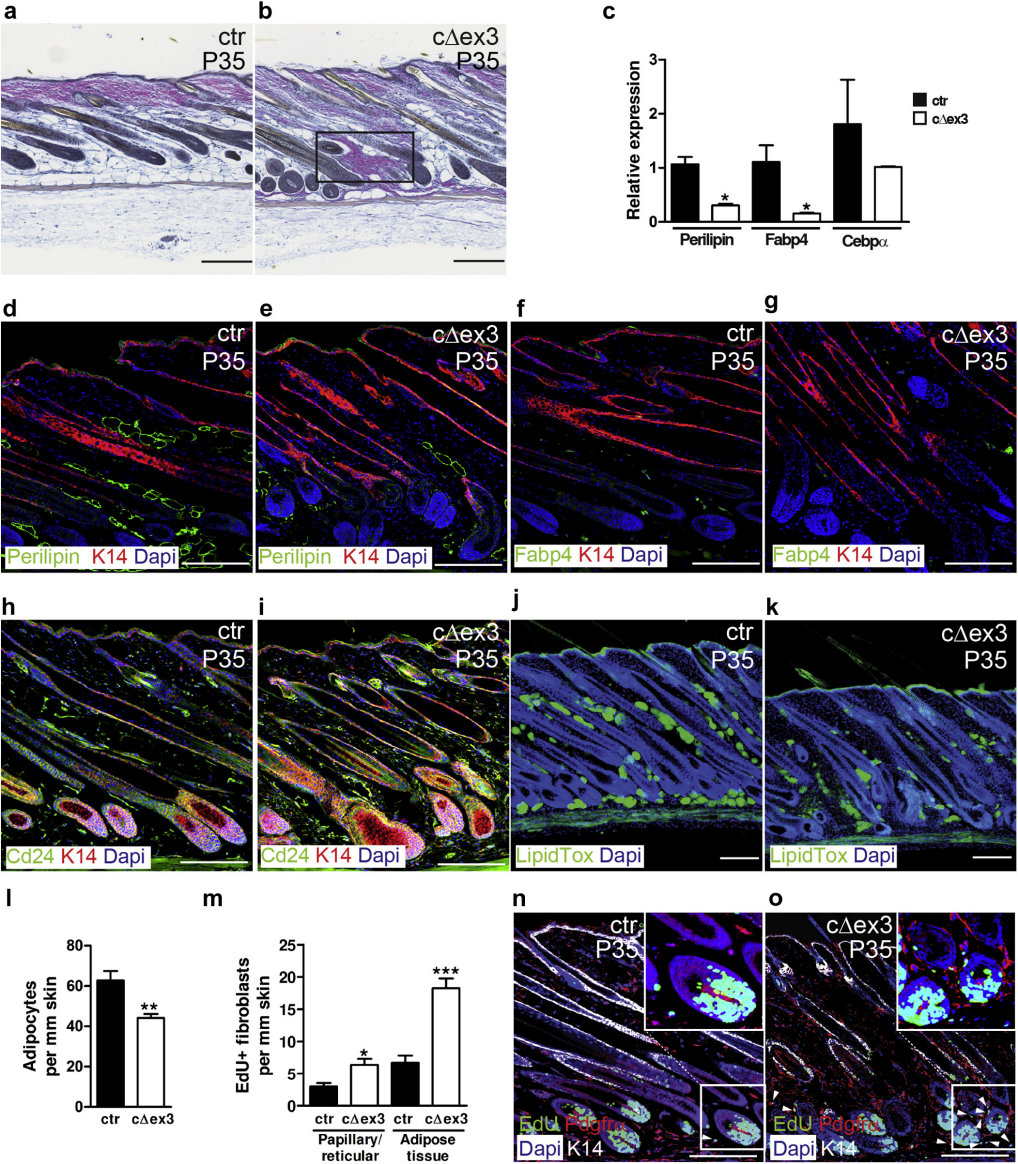
**Induction of dermal fibrosis and proliferation by β-catenin stabilization.(a, b)** Herovici’s staining differentiates between immature collagen fibers (blue) versus mature collagen (pink). Note fibrotic region with mature extracellular matrix in **(b)** (boxed). **(c)** Relative expression of adipocyte differentiation genes in flow-sorted tdtomato^+^ fibroblasts. Data represent mean ± standard error of the mean (n = 3). ^∗^*P* ≤ 0.05. **(d–i)** Immunostained paraffin sections of 35-day-old mutant and control skin. **(j, k)** Whole-mount thick sections stained with LipidTox (green) with DAPI counterstain (blue). **(l, m)** Quantification in paraffin sections of P35 skin of **(l)** adipocytes and **(m)** EdU^+^ fibroblasts in the adipocyte layer and interfollicular dermis (including papillary fibroblasts, dermal sheath, and reticular fibroblasts). The boundary between papillary/reticular and adipocyte layers was defined by the interface between the bottom of the reticular layer and the upper layer of differentiated adipocytes. Data points represent mean ± standard error of the mean; n ≥ 5 for each group. ^∗^*P* ≤ 0.05; ^∗∗^*P* ≤ 0.005; ^∗∗∗^*P* ≤ 0.0005. **(n, o)** Skin labeled with EdU (green) for 4 hours before harvesting, costained for Pdgfrα (red), keratin 14 (white), and DAPI (blue). White arrowheads indicate EdU^+^ fibroblasts within the adipose layer. Inserts are higher-magnification images of the boxed areas. Scale bars = 200 μm. ctr, control; DAPI, 4′, 6-diamidino-2-phenylindole; K14, keratin 14.

To characterize the effect of activated Wnt/β-catenin signaling in fibroblasts, we focused on P35 skin (Figure 4). The fibrotic areas within the adipose layer of cΔex3 dermis labeled pink with the histochemical Herovici’s stain, indicative of the mature fibrillar collagen (Collins et al., 2011) (Figure 4a and b). QPCR showed reduced expression of differentiation markers such as Fabp4, Perilipin, and Cebpα (Figure 4c) in mutant tdTomato^+^/Sca1^+^ fibroblasts compared with controls. Immunostaining confirmed reduced protein expression of Perilipin and Fabp4 but showed higher levels of the preadipocyte marker Cd24 (Figure 4d–i). The reduction in the number of differentiated adipocytes was confirmed by LipidTox (Invitrogen/Thermo Fisher Scientific, Waltham, MA) staining (Figure 4j and k) and was statistically significant (Figure 4l). Fibroblast proliferative activity, measured by 5-Ethynyl-2'-deoxyuridine (EdU) labeling, was increased in both the adipocyte layer and in the rest of the dermis, including papillary fibroblasts of the dermal sheath and reticular fibroblasts, the effect being most pronounced in the adipocyte layer (Figure 4m–o). However, we did not detect significant numbers of EdU^+^ DP cells, consistent with our previous finding that the number of proliferating cells in adult DP is very low, even when β-catenin is stabilized and the DP increases in size (Kaushal et al., 2015).

### Selective Wnt/β-catenin stabilization in Dlk1^+^ fibroblasts results in fibrosis within the adipose layer

**Figure 5:**
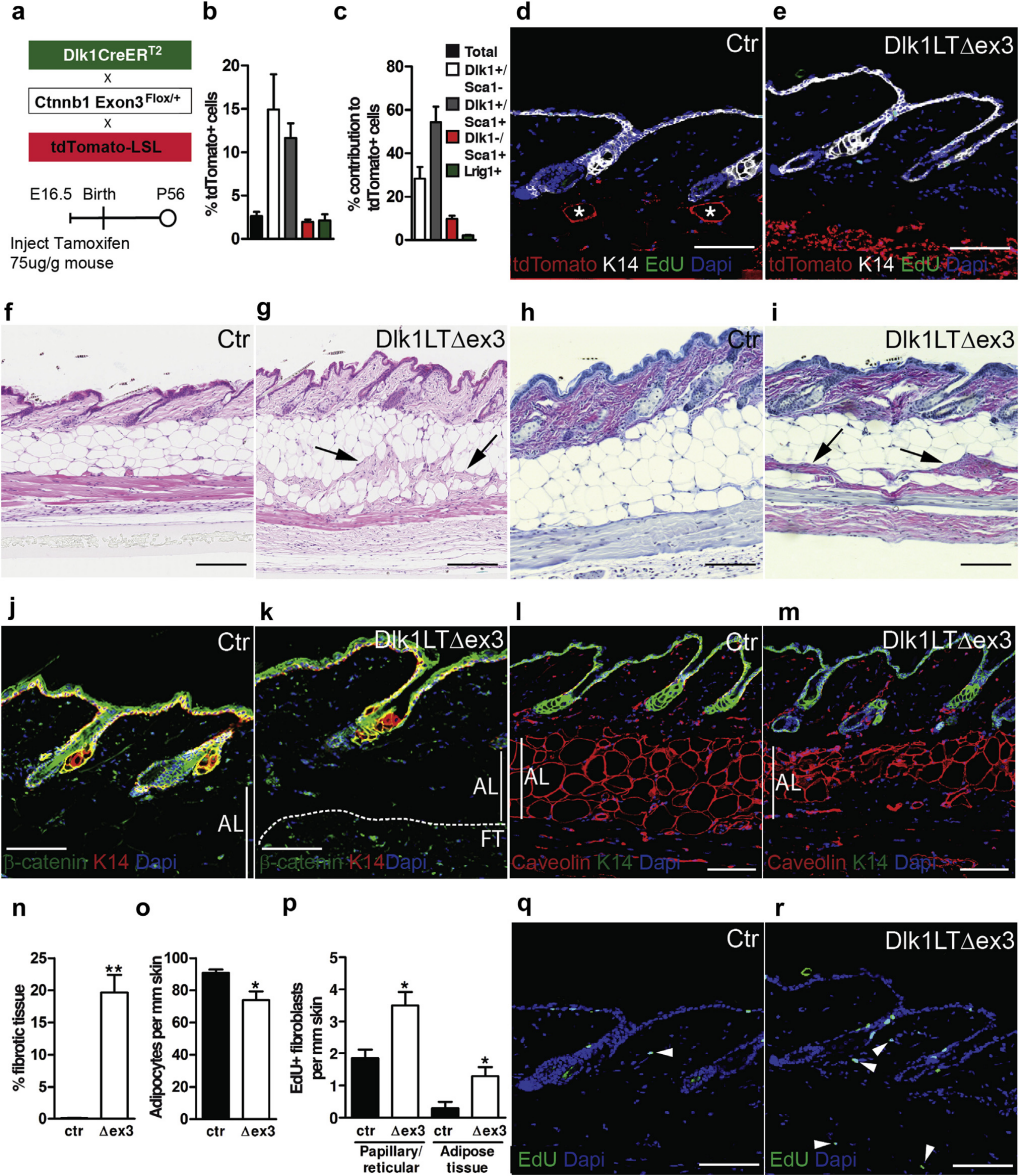
**β-Catenin stabilization in the lower dermis via Dlk1CreER^T2^.(a)** Schematic illustration of experimental strategy. **(b, c)** Quantification of tdTomato^+^ cells in E18.5 dermal cell suspensions (ITGA6^-^/LIN^-^/CD31^-^) 2 days after 4-OHT injection. **(b)** Percentage of tdTomato^+^ cells in each fibroblast subpopulation. **(c)** Contribution of each fibroblast subpopulation to tdTomato^+^ fibroblasts. Data are presented as mean ± standard error of the mean of quadruplicate samples in a representative experiment (n = 3 independent experiments). **(d, e)** tdTomato-expressing cells in P56 back skin of Dlk1CreER^T2^ × tdTomato-LSL × Ctnnb1 Exon3^+/+^ (control) and Dlk1CreER^T2^ × tdTomato-LSL × Ctnnb1 Exon3^Flox/+^ (Dlk1LTΔex3) mice. Asterisks in **(d)** indicate terminally differentiated adipocytes. **(f, g)** Hematoxylin and eosin and **(h, i)** Herovici’s staining of paraffin sections of P56 control and Dlk1LTΔex3 mouse back skin. Arrows in **(g)** and **(i)** indicate fibrotic regions. **(j–m)** P56 control and Dlk1LTΔex3 back skin labeled for β-catenin or caveolin-1 and keratin 14, counterstained with DAPI. Vertical lines indicate thickness of adipocyte layer. Dashed line in **(k)** demarcates fibrotic tissue (FT). **(n, o)** Quantification of fibrotic area shown as percentage of the adipocyte layer **(n)** and number of adipocytes **(o)**. **(p)** Quantification of EdU-labeled fibroblasts in the interfollicular dermis (including papillary fibroblasts, dermal sheath, and reticular fibroblasts) and adipose layer (defined as in Figure 4m) after a 2-hour EdU pulse. Data are presented as mean ± standard error of the mean of triplicate samples in a representative experiment (n = 3–5). ^∗^*P* ≤ 0.05; ^∗∗^*P* ≤ 0.005. **(q, r)** EdU staining of sections shown in **(d)** and **(e)**. Scale bars = 200 μm. 4-OHT, 4-hydroxy-tamoxifen; AL, adipocyte layer; ctr, control; DAPI, 4′, 6-diamidino-2-phenylindole; K14, keratin 14.

Given the differential expression of adipogenic genes in Sca1^+^ and Sca1^-^ fibroblasts at P2 (Figure 2) and the fibrosis within the adipose layer on fibroblast-specific stabilization of β-catenin (Figure 3), we hypothesized that the fibrotic effect of activating Wnt/β-catenin signaling in all Pdgfrα^+^ fibroblasts would be reproduced by selectively targeting the lower dermal lineage during skin development. Because fibroblasts that express Dlk1 at E16.5 give rise to the Sca1^+^ fibroblasts and mature adipocytes present at P2 and in adult skin (Driskell et al., 2013), we crossed Ctnnb1 Exon3^Flox/+^, Dlk1CreER^T2^, and tdTomato-LSL mice and treated them with tamoxifen at E16.5 to target the lower dermal lineage (Figure 5a).

When fibroblasts were isolated at E18.5, 2 days after tamoxifen injection in utero, 3% of total Pdgfrα^+^ fibroblasts were labeled (Figure 5b; Figure S1b). Ten percent to 15% of Dlk1^+^/Sca1^-^ and Dlk1^+^/Sca1^+^ cells were labeled, compared with 2% of Dlk1^-^/Sca1^+^ cells (Figure 5b; see Supplementary Figure S1b) and 1% of Lrig1^+^ papillary fibroblasts, confirming the selectivity of targeting. tdTomato^+^ fibroblasts comprised 30% Dlk1^+^/Sca1^-^, 55% Dlk1^+^/Sca1^+^ cells, and 10% Dlk1^-^/Sca1^+^ cells (Figure 5c). It is most likely that tdTomato^+^ cells within the Dlk1^-^/Sca1^+^ fibroblast population correspond to cells that were Dlk1^+^ at E16.5 but subsequently down-regulated Dlk1 expression (Driskell et al., 2013). When examined at P56, tdTomato^+^ cells were confined to the lower dermis in both control and Dlk1LTΔex3 skin (Figure 5d and e). In control skin most tdTomato^+^ cells had the morphology of mature adipocytes, whereas in Dlk1LTΔex3 skin the tdTomato^+^ cells had a fibroblastic morphology (Figure 5d and e; mature adipocytes are labeled with asterisks in Figure 5d).

In contrast to the effect of activating Wnt/β-catenin signaling in all fibroblasts, selective activation in Dlk1^+^ cells did not disturb the hair cycle, and at P56 both control and Dlk1LTDex3 skin was in telogen (Figure 5f and g). Nevertheless, there was an accumulation of fibroblasts within the adipose layer in Dlk1LTΔex3 skin (Figure 5f and g). These regions stained positively for fibrillar ECM using Herovici’s dye (Figure 5h and i) and expressed high levels of β-catenin (Figure 5j and k). The reduction in adipocyte numbers was confirmed by labeling for caveolin-1, a marker for preadipocytes and adipocytes (Figure 5l and m) and was statistically significant, in accordance with the appearance of fibrotic lesions (Figure 4n and o). Stabilization of β-catenin via Dlk1CreER^T2^ also resulted in an increase in fibroblast proliferation both in the adipocyte layer and throughout the dermis (Fig. 5p–r).

### Postnatal Wnt/β-catenin stabilization inhibits adipogenesis of Sca1^+^ fibroblasts in culture

**Figure 6:**
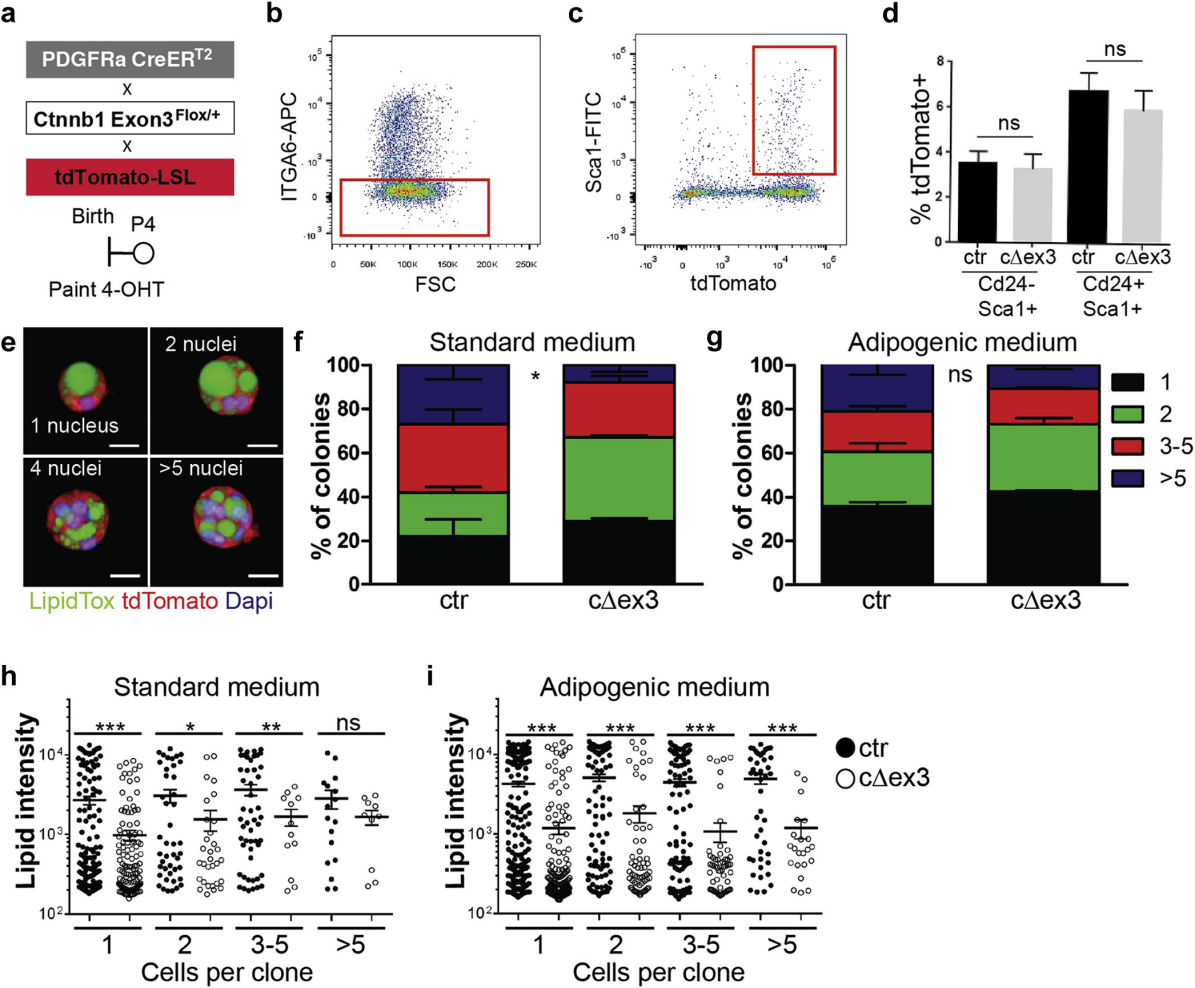
**Effect of β-catenin stabilization on adipocyte differentiation in cultured Sca1^+^ fibroblasts.(a)** Schematic illustration of experimental strategy. **(b–d)** Sca1^+^/tdTomato^+^ targeted fibroblasts from dermal cell suspensions of PDGFRαCreER^T2^ × tdTomato-LSL × Ctnnb1 Exon3^+/+^ (control) and PDGFRαCreER^T2^ × tdTomato-LSL × Ctnnb1 Exon3^Flox/+^ (cΔex3) littermates were isolated by flow cytometry. **(b, c)** Gating out α6 integrin-positive cells (ITGA6; keratinocytes) **(b)** and positive selection for Sca1^+^/tdTomato^+^ cells **(c)**. **(d)** Recombination efficiency (percentage tdTomato^+^ cells) in Cd24^+^/Sca1^-^ and Cd24^-^/Sca1^+^ cells. Data are presented as mean ± standard error of the mean of triplicate samples in a representative experiment (n = 3 independent experiments). **(e)** Examples of individual colonies formed by Sca1^+^ fibroblasts stained for LipidTox (green), tdTomato (red), and DAPI (blue). Scale bars = 10 μm. **(f, g)** Percentage of colonies containing 1, 2, 3–5, or more than 5 cells in standard **(f)** and adipogenic **(g)** medium. **(h, i)** Total LipidTox fluorescence (lipid intensity) per clone in control and mutant (cΔex3) cultures grown in standard **(h)** or adipogenic **(i)** medium. **(f–i)** n = 2–3 biological replicates and 2 technical replicates. ^∗^*P* ≤ 0.05, ^∗∗^*P* ≤ 0.01, ^∗∗∗^*P* ≤ 0.005. **(f, g)** two-way analysis of variance with a Bonferroni posttest. **(h, i)** 25% confidence intervals are shown. 4-OHT, 4-hydroxy-tamoxifen; ctr, control DAPI, 4′, 6-diamidino-2-phenylindole; FSC, forward scatter; ns, difference not significant.

To establish whether β-catenin stabilization within preadipocytes resulted in an inhibition of differentiation, we analyzed the behavior of single cells captured in ECM-functionalized hydrogels, as described previously (Driskell et al., 2012, Driskell et al., 2013). PdgfrαCreER^T2^, tdTomato-LSL, and Ctnnb1 Exon3^Flox/+^ mice were crossed and treated with 4-OHT on the day of birth (Figure 6a). Sca1^+^ tdTomato^+^ fibroblasts were isolated by flow cytometry 2 days later (Figure 6b and c). Activation of Wnt/β-catenin signaling did not affect the proportion of Sca1^+^ cells that expressed the preadipocyte marker Cd24 (Festa et al., 2011) (Figure 6d).

Sca1^+^ cells were encapsulated at clonal density in 3-dimensional hydrogels and cultured in control or adipogenic medium. After 10 days in culture, individual clones were scored for cell number and the total intensity of LipidTox staining (Figure 6e). Whereas β-catenin stabilization in vivo stimulated proliferation within the adipose layer (Figure 4m), there was no effect on clone size when Sca1^+^ cells were cultured in adipogenic medium, and in standard medium the only effect was to increase the percentage of two cell clones at the expense of clones with higher cell numbers (Figure 6f and g). Wnt/β-catenin signaling activation did, however, decrease adipocyte differentiation, as evaluated by decreased LipidTox staining, both in standard medium (Figure 6h) and in medium supplemented with adipogenic factors, regardless of clone size (Figure 6i). These findings suggest that the effect of β-catenin stabilization in neonatal fibroblasts is to prevent Sca1^+^ fibroblasts from undergoing adipocyte differentiation in a cell-autonomous manner, rather than changing the proportion of Cd24^+^/Sca1^+^ preadipocytes.

## Discussion

Here we have examined the effect of dermal Wnt/β-catenin signaling in the context of fibroblast heterogeneity. We show that expression of Wnt pathway genes such as *Wnt5a, Lef1, Tcf4,* or *Dkk1* differs between upper (Sca1^-^) and lower (Sca1^+^) dermis. Interestingly, immunostaining showed that β-catenin and Lef1 are more abundant in Sca1^-^ cells. β-Catenin stabilization in all fibroblast populations in neonatal skin resulted in a decrease in mature adipocytes and the appearance of fibrotic regions in the adipose layer, accompanied by stimulation of fibroblast proliferation throughout the dermis. The replacement of adipocytes by ectopic reticular fibroblasts also occurred when the lower dermal lineage was selectively targeted at E16.5, before adipocyte differentiation.

Our in vitro studies showed that β-catenin stabilization did not drive fibroblast proliferation cell-autonomously. The effect of β-catenin stabilization was to inhibit terminal differentiation of Sca1^+^ cells rather than to promote expansion of preadipocytes or selective proliferation of reticular fibroblasts. This is similar to the effect of β-catenin stabilization on cultured human keratinocytes, which is to expand the stem cell compartment without stimulating proliferation (Zhu and Watt, 1999). However, although we did not detect an expansion of preadipocytes within 4 days of β-catenin stabilization in vivo, Cd24 expression was increased in P35 skin. This could either be caused by an expansion of preadipocytes at a later stage, which is supported by increased fibroblast proliferation in vivo, or by the differentiation defect.

Our conclusion that targeting the lower dermal lineage accounts for the appearance of fibrotic regions is consistent with other studies highlighting the contributions of different dermal cell subpopulations to fibrosis. For example, fibroblasts that express engrailed 1 during development are responsible for the bulk of ECM deposition in dorsal dermis and mediate dermal fibrosis in response to irradiation (Rinkevich et al., 2015). Other dermal subpopulations that contribute to fibrosis include Sox2^+^ cells (Liu et al., 2014) and adiponectin-positive adipocyte precursors (Marangoni et al., 2015). In addition, Adam12^+^ perivascular mesenchymal cells expressing Pdgfrα and Sca1 are profibrotic in response to injury (Dulauroy et al., 2012).

The inhibition of adipocyte differentiation was not the only effect of dermal β-catenin activation that we observed. There was an increase in proliferation throughout the dermis, consistent with an earlier report (Cheon et al., 2002), and a disruption of the HF cycle. The effects on the hair cycle cannot be attributed solely to a decrease in mature adipocytes (Donati et al., 2014, Festa et al., 2011), because they were observed when we targeted all fibroblast subpopulations via PdgfrαCreER^T2^ and not when we selectively targeted the lower dermal lineage. This raises an interesting question as to whether increased proliferation of the fibroblast subpopulations that are known to regulate hair growth, in particular the DP, dermal sheath, and other papillary fibroblasts (Driskell et al., 2013, Enshell-Seijffers et al., 2012, Kaushal et al., 2015), is responsible. We have previously shown that β-catenin stabilization in Prominin-1–expressing DP cells results in an increase in DP size, but because the number of EdU^+^ cells is very low, the increase could be attributable to a number of factors, such as increased migration from the dermal sheath (Kaushal et al., 2015). Because the PdgfrαCreER^T2^ transgene is active in DP cells (Figure 3g and h), it is possible that the perturbed hair cycle reflects, at least in part, Wnt/β-catenin activation in DP cells. The effect of β-catenin stabilization on proliferation in the upper and lower dermis could either be direct or indirect. An indirect effect could be via dermal ECM, because Tcf-mediated transcription of several ECM protein-coding genes has recently been shown in a fibrosis model of sustained β-catenin activity in Hoxb2-derived ventral fibroblasts (Hamburg-Shields et al., 2015).

Our findings are in good agreement with previous reports that β-catenin plays a role in skin fibrosis (Beyer et al., 2012) and causes a down-regulation of adipogenic effector pathways, such as cEBP/PPARγ, in culture (Gesta et al., 2007). In addition, expression of the Wnt inhibitor Dkk1 interferes with profibrotic signaling (Akhmetshina et al., 2012). Together, these studies suggest that pharmacological modulation of Wnt signaling could be beneficial in the treatment of fibrotic skin diseases, including scleroderma (Ohgo et al., 2013). Similar mechanisms could also be responsible for the regulation of mesenchymal progenitors in other organs, including Pdgfrα^+^/Sca1^+^ muscle fibroblasts with proliferative and myofibroblast/adipogenic potential in response to muscle regeneration and ectopic fat formation (Uezumi et al., 2010).

## Materials and Methods

### In vivo experiments

The following mouse strains were maintained on a C57 Bl6/CBA background: Dlk1CreER^T2^ Institut Clinique de la Souris (ICS, Illkirch-Graffenstaden Cedex Alsace, France), PdgfrαEGFP (PdgfrαH2B-eGFP) (Jackson Laboratories, Bar Harbor, ME), Ctnnb1lox(ex3)/^+^ (Harada et al., 1999). PdgfrαCreER^T2^ (Rivers et al., 2008) and Rosa-CAG-LSL-tdTomato (Jackson Laboratories; 007905) strains were maintained as homozygotes. Cre-mediated recombination was induced by topical application of 2 mg of 4-hydroxy-tamoxifen (Sigma, St. Louis, MO; diluted in acetone + 10% DMSO) on the back skin of neonatal pups at P0–P1. For lineage tracing experiments, plugged females received a single intraperitoneal injection of 25 μg of tamoxifen per gram of body weight at E16.5. Tamoxifen was dissolved in corn oil (10–20 mg/ml) by intermittent sonication at 37°C for 20–30 minutes. Pups were harvested and fostered after surgical removal between E18.5 and E21. For in vivo proliferation assays, mice received a dose of 500 μg 5-ethynyl-2′-deoxyuridine (EdU; 2.5mg/ml in phosphate buffered saline) by intraperitoneal injection 2–4 hours before they were killed. Cohorts included male and/or female littermates, and results are representative of at least three biological samples. All experimental procedures were carried out under the terms of a UK Home Office project license after ethical review at Cambridge University or King’s College London.

### Histology

Skin samples were harvested and processed to generate paraffin (5 μm) or thick cryopreserved whole-mount tissue (50–100 μm) sections as previously described (Collins et al., 2011, Driskell et al., 2013). Immunostaining was performed using the following antibody combinations: rabbit anti-Rfp (1:300; Rockland, Limerick, PA)/rabbit anti-Perilipin 1A (1:100; Abcam, Cambridge, UK)/rabbit anti-Caveolin-1 (1:100; Abcam); rabbit anti-Fabp4 (1:100; Abcam); donkey anti-rabbit Alexa Fluor 555, 594, or 488; goat anti-PDGFRα and goat anti-Sca1 (1:100; R&D Systems, Minneapolis, MN); donkey anti-goat Alexa Fluor 488 or 647; chicken or rabbit anti-Keratin 14 (1:100; Covance, Princeton, NJ); goat anti-chicken Alexa Fluor 647 or 555; mouse β-catenin (1:100; BD Transduction, Lexington, KY); donkey anti-mouse Alexa Fluor 594 or 488; rabbit anti-TCF3/4 (1:100; Abcam) and anti-Lef1 (1:100; Cell Signalling, Danvers, MA); Alexa Fluor 488 conjugated anti-CD24 (1:300; BD Pharmingen, Franklin Lakes, NJ). LipidTox (1:500 in phosphate buffered saline; Invitrogen/Thermo Fisher Scientific, Waltham, MA) was used to stain neutral lipids in adipocytes. ProLong Gold antifade reagent (Invitrogen) or glycerol was used for mounting slides or whole-mount sections, counterstained with 4′, 6-diamidino-2-phenylindole (DAPI). The EdU click-it imaging kit (Invitrogen) was used for detecting incorporated EdU nucleoside analogs in proliferating cells. Herovici’s staining was performed as previously described (Collins et al., 2011).

### Image acquisition and quantification

Immunostained tissue sections were imaged using a Nikon A1R confocal microscope. Adobe Photoshop CS6 (Adobe, San Jose, CA) was used to adjust images and correct background. Bright field images were collected using either a Hamamatsu NanoZoomer slide scanner (Hamamatsu, Japan) or a Zeiss Axiophot microscope (Carl Zeiss, Oberkochen, Germany) with a ×10 objective and an AxioCam HRc camera. Image measurements were performed on at least six microscopic fields per biological sample.

### Cell isolation and flow cytometry

Dermis was separated from back skin of embryos (E18.5–E19.5) or postnatal pups (P2–P4) by incubation with thermolysin (0.25 mg/ml) (Sigma T7902) overnight at 4°C, digested in Dulbecco’s Modified Eagle’s Medium + 10% fetal bovine serum containing 2.5 mg/mL collagenase I (Gibco 17100-017; Gibco/Thermo Fisher Scientific, Waltham, MA), and further processed as previously described (Collins et al., 2011). Cells were labeled in phosphate buffered saline + 10% fetal bovine serum TruStain fcX anti-mouse blocking buffer with the following antibodies: anti-mouse Ly-6A/E (Sca-1)-Alexa Fluor 488, 700, or BV605 (clone D7; 1:200); CD140a/Pdgfra-APC (clone APA5; 1:20); CD45-Alexa Fluor 700 (clone 30-F11; 1:100); CD24-Fitc (Clone M1/69; 1:100) (eBioscience, San Diego, CA); Lrig1-Alexa Fluor 488 (polyclonal; 1:20) (RnD Systems); anti-rat Dlk1-Fitc (clone 24-11; 1:20) (MBL International, Woburn, MA); anti-mouse-CD31-Alexa Fluor 647 (BD Pharmingen); APC Mouse Lineage Antibody Cocktail (BD Pharmingen); and anti-human CD49f-Alexa Fluor 647 (clone NKI-GoH3; 1:20) (AbD Serotec, Kidlington, UK). A BD LSRFortess a (Becton Dickinson, Franklin Lakes, NJ) was used for flow cytometry and an Aria II (Becton Dickinson, Franklin Lakes, NJ) for sorting fibroblasts. Dead cells were excluded from analysis using DAPI. Fibroblasts were either sorted for CD140α expression or negatively selected by excluding epidermal, endothelial, and immune cells from the cell suspension (ITGA6^-^/LIN^-^/CD31^-^). Data analysis and visualization were performed using FlowJo software version 7.6.5 (Tree Star, Ashland, OR).

### Hydrogel culture and high-content imaging

Sorted fibroblasts were collected and encapsulated in Extracel (Glycosan Biosystems) as previously described (Driskell et al., 2012), at a density of 5 × 10^5^/ml in μClear 96-well plates (Corning). Cultures were maintained for 10 days at 37°C/5% CO_2_ in standard medium (Dulbecco’s modified Eagle’s medium + 10% fetal bovine serum + 1% penicillin/streptomycin) or Adipogenic medium (StemXVivo Osteogenic/Adipogenic Base Media with Adipogenic Supplement; R&D Systems), and the medium was changed every 2–3 days.

Cultures were fixed with 4% paraformaldehyde for 10 minutes, washed twice in phosphate buffered saline, permeabilized with 0.1% Triton X-100 for 5 minutes and stained with LipidTox, DAPI, and rabbit anti-Rfp (1:300 dilution; Abcam). Multiple image stacks of each colony were recorded using an Operetta High Content Imaging system (Perkin Elmer, Waltham, MA). Spheres were identified by staining for tdTomato, and the number of nuclei per sphere was determined using quantification algorithms in the Columbus analysis software (Perkin Elmer, Waltham, MA). The fluorescence intensity of LipidTox staining was measured for each sphere.

### Quantification and Statistics

GraphPad Prism 6 software (GraphPad Software Inc., La Jolla, CA) was used for all analyses of numerical data and generation of graphs and statistical tests, including one-way analysis of variance or Student *t* test. Error bars represent standard error of the mean.

### Microarray

Genome-wide expression profiling was carried out by the Paterson Institute Microarray Core Facility, as described previously (Collins et al., 2011). The data are deposited in the National Institutes of Health GEO repository under accession number GSE76751. Complementary DNA was hybridized to Affymetrix MG430.2A arrays (Affimetrix, Santa Clara, CA). Array images produced by the Affymetrix PICR 3000 scanner were imported as CEL files into Genespring GX11 (Agilent Technilogies, Santa Clara, CA) for analysis. Robust Multi-array Average normalization (baseline to median of all samples) was used. Analyses were performed on genes selected for expression above the bottom 20th percentile in all three samples within at least one of two experimental groups. To identify differentially expressed genes, we compared the two groups using the Student *t* test *(P*-value cut-off of 0.05). Entities regulated by greater than 2-fold were selected for further analysis.

## ORCIDs

Beate M. Lichtenberger: http://orcid.org/0000-0001-6882-0257

Maria M. Mastrogiannaki: http://orcid.org/0000-0003-4978-2237

Fiona M. Watt: http://orcid.org/0000-0001-9151-5154

## Conflict of Interest

The authors state no conflict of interest.

## Acknowledgments

This work was supported by grants to FMW from the Medical Research Council and the Wellcome Trust. MM and BML were supported by long-term postdoctoral fellowships from the European Molecular Biology Organization (MM) and Federation of European Biochemical Societies (BML). We gratefully acknowledge excellent support from staff at King’s College London and Wellcome Trust Centre for Stem Cell Research, particularly J. Harris, Head of the Nikon Microscopy Centre at King’s. We gratefully acknowledge financial support in the form of access to the Biomedical Research Centre flow cytometry core of Guy’s & St. Thomas’ National Health Service (NHS) Foundation Trust.

## Author Contributions

MM and BML equally contributed to performing and analyzing the experiments shown in Figure 2, Figure 3, Figure 4, Figure 5, Figure 6. AR performed and analyzed the experiments shown in Figure 6. CAC generated and analyzed the microarray data, which were validated by RRD, and generated data in Figures 1 and 2. FMW contributed to experimental design and analysis. MM, BML, and FMW wrote the manuscript. All authors contributed to interpreting the findings and editing the final manuscript.
