# Supplementary material for: β-Catenin Stabilization in Skin Fibroblasts Causes Fibrotic Lesions by Preventing Adipocyte Differentiation of the Reticular Dermis
Source: J Invest Dermatol. 2016 Jun;136(6):1130–42. doi: 10.1016/j.jid.2016.01.036 (PMC4874948; doi:10.1016/j.jid.2016.01.036)
Supplement: Supplementary Figure S1 and Supplementary Table S1 [file mmc1.pdf]

SUPPLEMENTAL MATERIAL

β-catenin stabilization in skin fibroblasts causes fibrotic lesions by preventing adipocyte differentiation of the reticular dermis

Maria Mastrogiannaki[1, 2, 3], Beate M. Lichtenberger[1, 2, 3], Andreas Reimer[1], Charlotte A. Collins[2], Ryan R. Driskell[1] and Fiona M. Watt[1]*

[1]Centre for Stem Cells and Regenerative Medicine, King's College London, Guy's Hospital, Great Maze Pond, London SE1 9RT, UK

[2] Wellcome Trust Centre for Stem Cell Research, University of Cambridge, Tennis Court Road, Cambridge CB2 1QR, UK

[3] Co-first authors

INVENTORY OF SUPPLEMENTAL MATERIAL

Supplementary Figure S1 Fibroblast-specific stabilization of β-catenin

Supplementary Table S1 Differentially regulated genes

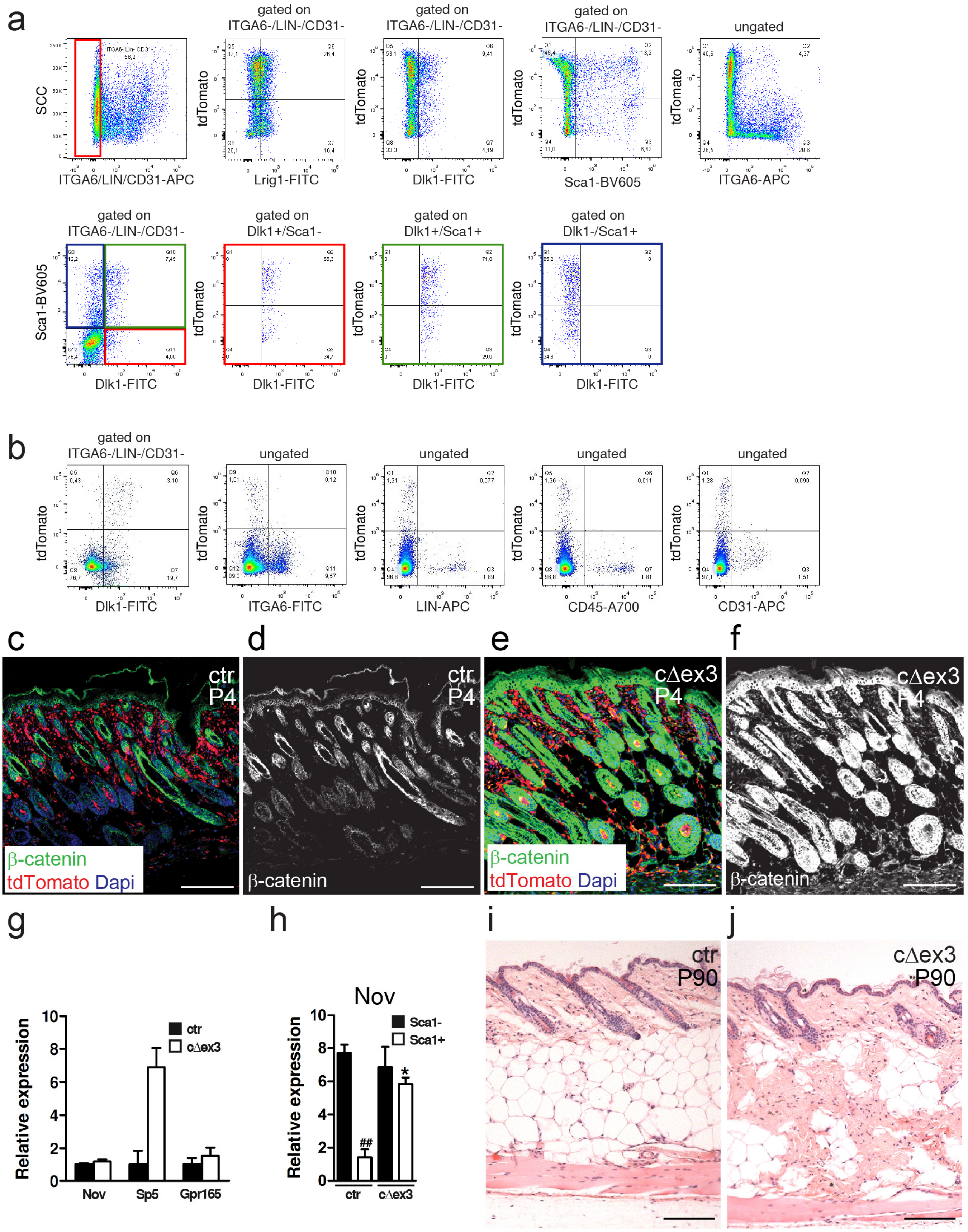

a
gated on ITGA6-/LIN-/CD31-
ungated
SCC
ITGA6/LIN/CD31-APC
tdTomato
Lrig1-FITC
Dlk1-FITC
Sca1-BV605
ITGA6-APC
gated on Dlk1+/Sca1-
gated on Dlk1+/Sca1+
gated on Dlk1-/Sca1+
b
ITGA6-FITC
LIN-APC
CD45-A700
CD31-APC
c
ctr P4
β-catenin
tdTomato Dapi
d
e
cΔex3 P4
f
g
Relative expression
ctr
cΔex3
Nov
Sp5
Gpr165
h
Nov
Sca1-
Sca1+
##
*
i
ctr P90
j
cΔex3 P90

Supplementary Figure S1. Fibroblast-specific stabilization of β-catenin (a) Gating strategy for quantifying different subpopulations of $tdTomato^{+}$ cells in Figure 3b. Representative plots are shown. (b) Specificity of $Dlk1CreER^{T2}$ tdTomato lineage tracing in Figure 5b. $ITGA6^{-}/LIN^{-}/CD31^{-}$ cells were gated as in (b). Representative plots show that recombination occurs in Dlk1+ fibroblasts but not in epidermal, endothelial or hematopoietic cells.(c-f) Immunofluorescent staining for β-catenin, tdTomato and DAPI (c, e). (d, f) show the β-catenin channel of images in (c, e). (g, h) Relative expression of the indicated genes in flow-sorted $tdTomato^{+}$ fibroblasts isolated from P4 back skin (g) and flow-sorted $tdTomato^{+}/Sca1^{-}$ and $tdTomato^{+}/Sca1^{+}$ fibroblasts isolated from P56 back skin. Data show means ± SEM (n=3-4 biological samples pooled from 2-4 animals). * p≤0.05 compared to $Sca1^{+}$ control fibroblasts; ## p≤0.005 compared to $Sca1^{-}$ control fibroblasts. (i, j) H&E stained sections of back skin of P90 mice of the genotypes indicated. Note that the hair follicles are in telogen, even though there is an evident reduction in the adipocyte layers on expression of stabilized β-catenin via $Pdgfr\alpha CreER^{T2}$. Scale bars: 200 μm.

Supplementary Table S1. Differentially regulated genes in $Pdgfr\alpha EGFP^{+}/Sca1^{+}$ fibroblasts The list of entities is compiled from genes that are differentially regulated in $Pdgfr\alpha EGFP^{+}/Sca1^{+}$ fibroblasts compared to $Pdgfr\alpha EGFP^{+}/Sca1^{-}$ (T test p<0.05, fold change >2). Some genes are represented by more than one entity. Entities are ranked by fold change and grouped according to positive (up, red) or negative (down, blue) regulation.

Table S1

Differentially regulated genes in PdgfraEGFP+/ Sca1+ adipogenic fibroblasts (T test p<0.05, fold change >2).

Gene Symbol	Entrez Gene	Gene Title	Probe Set ID	Regulation	Fold Change
Bmper	73230	BMP-binding endothelial regulator	1429273_at	up	27,14704
BB144871	101918	expressed sequence BB144871	1436453_at	up	18,301313
4921521F21Rik	70861	RIKEN cDNA 4921521F21 gene	1423381_at	up	17,208042
Adipoq	11450	adiponectin, C1Q and collagen domain contaii	1422651_at	up	16,132975
Ly6a	110454	lymphocyte antigen 6 complex, locus A	1417185_at	up	16,116531
Fzd4	14366	frizzled homolog 4 (Drosophila)	1449416_at	up	15,118653
Chrdl1	83453	chordin-like 1	1434201_at	up	14,37288
Fzd4	14366	frizzled homolog 4 (Drosophila)	1419301_at	up	12,178625
AI607873	226691	expressed sequence AI607873	1457035_at	up	10,661243
Cd36	12491	CD36 antigen	1450883_a_at	up	10,644925
C1s /// LOC100044326	100044326 ///	complement component 1, s subcomponent /	1424041_s_at	up	10,448336
Ms4a4d	66607	membrane-spanning 4-domains, subfamily A,	1418990_at	up	10,355799
Car3	12350	carbonic anhydrase 3	1449434_at	up	10,344274
Esr1	13982	estrogen receptor 1 (alpha)	1435663_at	up	10,2470875
Cxcl1	14825	chemokine (C-X-C motif) ligand 1	1419209_at	up	10,177192
Slco2b1	101488	solute carrier organic anion transporter family	1433933_s_at	up	10,113922
Akap12	83397	A kinase (PRKA) anchor protein (gravin) 12	1419706_a_at	up	10,016486
Kcnj8	16523	potassium inwardly-rectifying channel, subfar	1418142_at	up	9,899502
Fmod	14264	fibromodulin	1456084_x_at	up	9,702447
Nell1	338352	NEL-like 1 (chicken)	1433787_at	up	9,688301
Scn7a	20272	sodium channel, voltage-gated, type VII, alpł	1436043_at	up	9,442539
Rbp4	19662	retinol binding protein 4, plasma	1426225_at	up	8,717569
Cd36	12491	CD36 antigen	1423166_at	up	8,633587
100040462 /// Ifi203 /// If	100040462 ///	predicted gene, 100040462 /// interferon acti	1452348_s_at	up	8,572408
Zcchc5	213436	zinc finger, CCHC domain containing 5	1437355_at	up	8,432949
C1r	50909	complement component 1, r subcomponent	1417009_at	up	8,374341
Esr1	13982	estrogen receptor 1 (alpha)	1460591_at	up	8,108602
5430421B17	330593	hypothetical protein 5430421B17	1434756_at	up	8,048973
Agtr2	11609	angiotensin II receptor, type 2	1415832_at	up	7,8949695
Lpl	16956	lipoprotein lipase	1431056_a_at	up	7,7214975
Figf /// LOC100047108	100047108 ///	c-fos induced growth factor /// similar to FIGF	1438953_at	up	7,6774178
Ldb2	16826	LIM domain binding 2	1456786_at	up	7,6740522
Fabp4	11770	fatty acid binding protein 4, adipocyte	1417023_a_at	up	7,6263037
Figf	14205	c-fos induced growth factor	1438954_x_at	up	7,602751
Serpina3n	20716	serine (or cysteine) peptidase inhibitor, clade	1419100_at	up	7,559107
667277 /// C1r	50909 /// 667	predicted gene, 667277 /// complement comp	1456437_x_at	up	7,4601974
Atp1a2	98660	ATPase, Na+/K+ transporting, alpha 2 polype	1443823_s_at	up	7,452678
Cxcl1	14825	chemokine (C-X-C motif) ligand 1	1441855_x_at	up	7,4385967
Zfhx4	80892	zinc finger homeodomain 4	1421433_at	up	7,1405125
Mfap5	50530	microfibrillar associated protein 5	1418454_at	up	7,113682
Acaa1b	235674	acetyl-Coenzyme A acyltransferase 1B	1424451_at	up	6,887252
Clec2d	93694	C-type lectin domain family 2, member d	1419477_at	up	6,7683125
Cxcl1	14825	chemokine (C-X-C motif) ligand 1	1457644_s_at	up	6,745072
Dlk1	13386	delta-like 1 homolog (Drosophila)	1449939_s_at	up	6,682756
Ifi204	15951	interferon activated gene 204	1419603_at	up	6,6689963
Ldb2	16826	LIM domain binding 2	1439557_s_at	up	6,5811977
Efemp1	216616	epidermal growth factor-containing fibulin-like	1427183_at	up	6,355791
Fabp4	11770	fatty acid binding protein 4, adipocyte	1425809_at	up	6,318707
Ifi205 /// Mnda	226695 /// 38	interferon activated gene 205 /// myeloid cell	1452349_x_at	up	6,3019567
Aoc3	11754	amine oxidase, copper containing 3	1449396_at	up	6,247015
Mc2r	17200	melanocortin 2 receptor	1422926_at	up	6,1915946
Chrdl1	83453	chordin-like 1	1456722_at	up	6,189348
Epha3	13837	Eph receptor A3	1425575_at	up	6,15246
LOC100045833 /// Ly6c1 /	100041546 ///	similar to Lymphocyte antigen 6C precursor (I	1421571_a_at	up	6,0910254
Abcc9	20928	ATP-binding cassette, sub-family C (CFTR/MR	1420408_a_at	up	6,087254
Lrrtm1	74342	leucine rich repeat transmembrane neuronal :	1455883_a_at	up	6,072831
Cd36	12491	CD36 antigen	1450884_at	up	6,015234
Esr1	13982	estrogen receptor 1 (alpha)	1421244_at	up	5,9748487
Tfpi2	21789	tissue factor pathway inhibitor 2	1418547_at	up	5,9365153
Adamtsl1	77739	ADAMTS-like 1	1442063_at	up	5,9051614
Atp1a2	98660	ATPase, Na+/K+ transporting, alpha 2 polype	1434893_at	up	5,8637347
Ccl11	20292	chemokine (C-C motif) ligand 11	1417789_at	up	5,858775
Lgr5	14160	leucine rich repeat containing G protein coupl	1450988_at	up	5,855163
Lgals3bp	19039	lectin, galactoside-binding, soluble, 3 binding	1448380_at	up	5,8506083
Nrk	27206	Nik related kinase	1450079_at	up	5,792295
Adh1	11522	alcohol dehydrogenase 1 (class I)	1416225_at	up	5,779169
Ntrk3	18213	neurotrophic tyrosine kinase, receptor, type 3	1443970_at	up	5,758859
Plat	18791	plasminogen activator, tissue	1415806_at	up	5,7580934
Serping1	12258	serine (or cysteine) peptidase inhibitor, clade	1416625_at	up	5,562655

Parp3	235587	poly (ADP-ribose) polymerase family, membe	1426210_x_at	up	5,543732
Pparg	19016	peroxisome proliferator activated receptor ga	1420715_a_at	up	5,523724
Nrk	27206	Nik related kinase	1450078_at	up	5,5109053
Tmeff2	56363	transmembrane protein with EGF-like and two	1419073_at	up	5,415139
Prelp	116847	proline arginine-rich end leucine-rich repeat	1416321_s_at	up	5,41482
Cxcl12	20315	chemokine (C-X-C motif) ligand 12	1448823_at	up	5,4073114
Chrdl1	83453	chordin-like 1	1421295_at	up	5,381881
Epha3	13837	Eph receptor A3	1455426_at	up	5,357103
Serpina3c	16625	serine (or cysteine) peptidase inhibitor, clade	1421564_at	up	5,3193135
Figf	14205	c-fos induced growth factor	1449528_at	up	5,3093524
Parp3	235587	poly (ADP-ribose) polymerase family, membe	1451969_s_at	up	5,2564473
Chrdl1	83453	chordin-like 1	1424945_at	up	5,2362556
Nrk	27206	Nik related kinase	1436399_s_at	up	5,1634164
Mfap5	50530	microfibrillar associated protein 5	1449082_at	up	5,1602807
Mgst1	56615	microsomal glutathione S-transferase 1	1415897_a_at	up	5,1572347
Osmr	18414	oncostatin M receptor	1418675_at	up	5,156055
Cygb	114886	cytoglobin	1423630_at	up	5,1455917
Iigp1	60440	interferon inducible GTPase 1	1419043_a_at	up	5,139909
Fndc1	68655	fibronectin type III domain containing 1	1453321_at	up	5,1210327
Serpinf1	20317	serine (or cysteine) peptidase inhibitor, clade	1453724_a_at	up	5,112359
Igfbp7	29817	insulin-like growth factor binding protein 7	1423584_at	up	5,0641994
Hmga2-ps1	15365	high mobility group AT-hook 2, pseudogene 1	1440559_at	up	4,93284
Ifi203	15950	interferon activated gene 203	1452231_x_at	up	4,9315233
Gpr133	243277	G protein-coupled receptor 133	1455466_at	up	4,912697
Prelp	116847	proline arginine-rich end leucine-rich repeat	1416322_at	up	4,901169
Zfhx4	80892	zinc finger homeodomain 4	1437556_at	up	4,8906484
A530020G20Rik	319839	RIKEN cDNA A530020G20 gene	1442580_at	up	4,8489046
Ldb2	16826	LIM domain binding 2	1421101_a_at	up	4,84303
Atp1a2	98660	ATPase, Na+/K+ transporting, alpha 2 polype	1427465_at	up	4,78578
Aldh1a1	11668	aldehyde dehydrogenase family 1, subfamily /	1416468_at	up	4,7747602
Hpgd	15446	hydroxyprostaglandin dehydrogenase 15 (NAI	1419905_s_at	up	4,7588973
Hic1	15248	hypermethylated in cancer 1	1449226_at	up	4,715873
Hrct1	100039781	histidine rich carboxyl terminus 1	1429018_at	up	4,7117043
Ifi203	15950	interferon activated gene 203	1425008_a_at	up	4,6957593
Hpgd	15446	Hydroxyprostaglandin dehydrogenase 15 (NA	1419906_at	up	4,6685324
1700112E06Rik	76633	RIKEN cDNA 1700112E06 gene	1430886_at	up	4,6549993
Tnxb	81877	tenascin XB	1450798_at	up	4,627588
EG328264	328264	predicted gene, EG328264	1459259_at	up	4,589858
Ctsh	13036	cathepsin H	1443814_x_at	up	4,5242057
Lpl	16956	lipoprotein lipase	1415904_at	up	4,494355
Adam23 /// LOC10004603!	100046035 ///	a disintegrin and metallopeptidase domain 23	1423378_at	up	4,4200745
Rassf2	215653	Ras association (RalGDS/AF-6) domain family	1428392_at	up	4,3245516
Atp8b1	54670	ATPase, class I, type 8B, member 1	1455396_at	up	4,3231254
Calcrl	54598	calcitonin receptor-like	1425814_a_at	up	4,3150687
Angptl4	57875	angiopoietin-like 4	1453410_at	up	4,2794147
Fap	14089	fibroblast activation protein	1417552_at	up	4,261906
Rtn4rl1	237847	reticulon 4 receptor-like 1	1436868_at	up	4,248162
			1444767_at	up	4,247442
Adrb1	11554	adrenergic receptor, beta 1	1423420_at	up	4,22082
			1457877_at	up	4,220345
Sdpr	20324	serum deprivation response	1443832_s_at	up	4,2172365
Gstm2	14863	glutathione S-transferase, mu 2	1416411_at	up	4,216173
Adam23	23792	a disintegrin and metallopeptidase domain 23	1447946_at	up	4,1744747
Ctsh	13036	cathepsin H	1418365_at	up	4,1694384
Ephx2	13850	epoxide hydrolase 2, cytoplasmic	1448499_a_at	up	4,1521993
Fmo1	14261	flavin containing monooxygenase 1	1417429_at	up	4,1405835
Pappa2	23850	pappalysin 2	1444451_at	up	4,1380935
Cyr61	16007	cysteine rich protein 61	1438133_a_at	up	4,118732
Steap3	68428	STEAP family member 3	1430355_a_at	up	4,1047072
Osmr	18414	oncostatin M receptor	1418674_at	up	4,0872116
Cmah	12763	cytidine monophospho-N-acetylneuraminic ac	1428043_a_at	up	4,0767946
Itm2a	16431	integral membrane protein 2A	1423608_at	up	4,074737
Il31ra	218624	interleukin 31 receptor A	1451535_at	up	4,0732465
Bdkrb2	12062	Bradykinin receptor, beta 2 (Bdkrb2), mRNA	1442187_at	up	4,0623283
Pcolce	18542	procollagen C-endopeptidase enhancer protei	1448433_a_at	up	4,030286
Tek	21687	endothelial-specific receptor tyrosine kinase	1418788_at	up	4,0298963
Dpep1	13479	dipeptidase 1 (renal)	1419674_a_at	up	4,0170083
Scn7a	20272	sodium channel, voltage-gated, type VII, alph	1436044_at	up	4,0156136
Gda	14544	guanine deaminase	1435749_at	up	4,013352
Mmp19	58223	matrix metallopeptidase 19	1421976_at	up	3,981666
Ntrk3	18213	neurotrophic tyrosine kinase, receptor, type 3	1433825_at	up	3,977062
Adamtsl1	77739	ADAMTS-like 1	1430313_at	up	3,974646
Cp	12870	ceruloplasmin	1417494_a_at	up	3,972882
C1qtnf3	81799	C1q and tumor necrosis factor related protein	1422606_at	up	3,9705317

Fabp4	11770	fatty acid binding protein 4, adipocyte	1451263_a_at	up	3,9685102
Nupr1	56312	nuclear protein 1	1419665_a_at	up	3,9595222
			1442025_a_at	up	3,9378865
Mpa2l	100702	macrophage activation 2 like	1438676_at	up	3,9218245
5031426D15Rik	68144	RIKEN cDNA 5031426D15 gene	1431248_at	up	3,91754
Gucy1a3	60596	guanylate cyclase 1, soluble, alpha 3	1420534_at	up	3,8879724
Tusc5	237858	tumor suppressor candidate 5	1441096_at	up	3,8748946
Slc1a3	20512	solute carrier family 1 (glial high affinity gluta	1426340_at	up	3,8738167
Cmah	12763	cytidine monophospho-N-acetylneuraminic ac	1421214_at	up	3,866772
Ptger4	19219	prostaglandin E receptor 4 (subtype EP4)	1421073_a_at	up	3,8659325
			1458830_at	up	3,842953
Nupr1	56312	nuclear protein 1	1419666_x_at	up	3,8193142
Cdkn1c	12577	cyclin-dependent kinase inhibitor 1C (P57)	1417649_at	up	3,8065464
Ptgfr	19220	prostaglandin F receptor	1446331_at	up	3,7845705
Klhl13	67455	kelch-like 13 (Drosophila)	1448269_a_at	up	3,7767868
Csf1	12977	colony stimulating factor 1 (macrophage)	1460220_a_at	up	3,7481172
Gas6	14456	growth arrest specific 6	1417399_at	up	3,745816
Dapk1	69635	death associated protein kinase 1	1427358_a_at	up	3,733323
			1443007_at	up	3,7240856
Klhl13	67455	kelch-like 13 (Drosophila)	1416242_at	up	3,704093
H2-Q10	15007	histocompatibility 2, Q region locus 10	1425137_a_at	up	3,68651
Ccdc80	67896	coiled-coil domain containing 80	1424187_at	up	3,6658614
Gpd1	14555	glycerol-3-phosphate dehydrogenase 1 (solub	1416204_at	up	3,6570053
Adam12	11489	a disintegrin and metallopeptidase domain 12	1421172_at	up	3,6525865
Klf14	619665	Kruppel-like factor 14	1457397_at	up	3,6475642
6530401D17Rik	76219	RIKEN cDNA 6530401D17 gene	1451679_at	up	3,626804
Lgals9	16859	lectin, galactose binding, soluble 9	1421217_a_at	up	3,6264517
Hsd11b2	15484	hydroxysteroid 11-beta dehydrogenase 2	1416761_at	up	3,617153
Ltbp2	16997	latent transforming growth factor beta bindin	1418061_at	up	3,6016116
Trim9	94090	tripartite motif-containing 9	1434249_s_at	up	3,5907178
Sdpr	20324	serum deprivation response	1416779_at	up	3,5722156
Ly6e	17069	lymphocyte antigen 6 complex, locus E	1453304_s_at	up	3,560402
Ang	11727	angiogenin, ribonuclease, RNase A family, 5	1438937_x_at	up	3,5559647
Col15a1	12819	collagen, type XV, alpha 1	1448755_at	up	3,5507078
Olfml1	244198	olfactomedin-like 1	1455663_at	up	3,546517
Slc40a1	53945	solute carrier family 40 (iron-regulated transp	1448566_at	up	3,5454712
Itm2a	16431	integral membrane protein 2A	1451047_at	up	3,5412915
Egr1	13653	early growth response 1	1417065_at	up	3,5284483
Ablim1	226251	actin-binding LIM protein 1	1453103_at	up	3,5256581
Itgbl1	223272	integrin, beta-like 1	1425039_at	up	3,5249536
Slc40a1	53945	solute carrier family 40 (iron-regulated transp	1417061_at	up	3,524047
Thbs1	21825	thrombospondin 1	1460302_at	up	3,5154676
Ntrk3	18213	neurotrophic tyrosine kinase, receptor, type 3	1425070_at	up	3,5122695
Mmp19	58223	matrix metallopeptidase 19	1421977_at	up	3,5067005
Cxcl10	15945	chemokine (C-X-C motif) ligand 10	1418930_at	up	3,4980295
Zfp385a	29813	zinc finger protein 385A	1418865_at	up	3,4744093
ENSMUSG00000043248 //	100039175 ///	predicted gene, ENSMUSG00000043248 /// p	1458659_at	up	3,4681306
Ntrk3	18213	neurotrophic tyrosine kinase, receptor, type 3	1425071_s_at	up	3,4678326
Ptgfr	19220	prostaglandin F receptor	1453924_a_at	up	3,4618323
Slc1a3	20512	solute carrier family 1 (glial high affinity gluta	1452031_at	up	3,4571385
Abcc9	20928	ATP-binding cassette, sub-family C (CFTR/MR	1435751_at	up	3,417256
Pdpn	14726	podoplanin	1419309_at	up	3,4048033
			1446659_at	up	3,4046845
Nova1	664883	neuro-oncological ventral antigen 1	1426938_at	up	3,4022992
			1458236_at	up	3,3991418
Rarres2	71660	retinoic acid receptor responder (tazarotene i	1428538_s_at	up	3,3966062
Epha3	13837	Eph receptor A3	1426057_a_at	up	3,3774095
2900046F13Rik	73028	RIKEN cDNA 2900046F13 gene	1432861_at	up	3,3744204
Rarres2	71660	retinoic acid receptor responder (tazarotene i	1437902_s_at	up	3,3663216
Steap4	117167	STEAP family member 4	1425829_a_at	up	3,3491008
Dlk1	13386	delta-like 1 homolog (Drosophila)	1442724_at	up	3,3386
Agtr1a	11607	angiotensin II receptor, type 1a	1436739_at	up	3,3361428
Zbtb16	235320	zinc finger and BTB domain containing 16	1419874_x_at	up	3,332064
Col5a3	53867	collagen, type V, alpha 3	1419703_at	up	3,3200917
Cyr61	16007	cysteine rich protein 61	1416039_x_at	up	3,3016484
Ptgir	19222	prostaglandin I receptor (IP)	1427313_at	up	3,296893
Dapk1	69635	death associated protein kinase 1	1426915_at	up	3,2940066
Ogn	18295	osteoglycin	1419662_at	up	3,2930143
Slc38a4	69354	solute carrier family 38, member 4	1428111_at	up	3,2899382
Lgr5	14160	leucine rich repeat containing G protein coupl	1444519_at	up	3,286038
Ptger3	19218	prostaglandin E receptor 3 (subtype EP3)	1450344_a_at	up	3,274178
Slc10a6	75750	solute carrier family 10 (sodium/bile acid cotr	1428776_at	up	3,2636373
Depdc6	97998	DEP domain containing 6	1451348_at	up	3,2567399
Ptgfr	19220	prostaglandin F receptor	1420349_at	up	3,2464437

Ripply3	170765	ripply3 homolog (zebrafish)	1420459_at	up	3,2420065
Sh3gl2	20404	SH3-domain GRB2-like 2	1418791_at	up	3,2380815
Tmem135	72759	transmembrane protein 135	1426435_at	up	3,2342296
Ecm1	13601	extracellular matrix protein 1	1448613_at	up	3,221523
Prr16	71373	proline rich 16	1431109_at	up	3,2213507
Cp	12870	ceruloplasmin	1448734_at	up	3,2197087
Ldhb	16832	lactate dehydrogenase B	1434499_a_at	up	3,211863
Ptger4	19219	prostaglandin E receptor 4 (subtype EP4)	1424208_at	up	3,2012646
Fank1	66930	fibronectin type 3 and ankyrin repeat domain:	1447495_at	up	3,2003927
			1434632_at	up	3,193838
Man1a	17155	mannosidase 1, alpha	1417110_at	up	3,1843834
2410085M17Rik	73706	RIKEN cDNA 2410085M17 gene	1440381_at	up	3,1805358
Psap	43727,66829	prosaposin	1415687_a_at	up	3,179044428
D530031A16Rik	78724	RIKEN cDNA D530031A16 gene	1433310_at	up	3,1696248
Dpep1	13479	dipeptidase 1 (renal)	1435943_at	up	3,1255274
Rnf144a	108089	ring finger protein 144A	1438404_at	up	3,1128757
Dmrt2	226049	doublesex and mab-3 related transcription fa	1426867_at	up	3,0973058
Ppap2b	67916	phosphatidic acid phosphatase type 2B	1448908_at	up	3,0812924
Tppp3	67971	tubulin polymerization-promoting protein fam	1416713_at	up	3,081029
Nfib	18028	nuclear factor I/B	1427680_a_at	up	3,0797112
OTTMUSG00000002879	100040792	predicted gene, OTTMUSG00000002879	1442917_at	up	3,0766215
Oxtr	18430	oxytocin receptor	1440888_at	up	3,0606337
Qpct	70536	glutaminyl-peptide cyclotransferase (glutamir	1426622_a_at	up	3,054099
Loxl1	16949	lysyl oxidase-like 1	1436063_at	up	3,0504107
G0s2	14373	G0/G1 switch gene 2	1448700_at	up	3,0444157
Cpz	242939	carboxypeptidase Z	1426251_at	up	3,0368688
Nfib	18028	nuclear factor I/B	1448288_at	up	3,0337949
Slc7a10	53896	solute carrier family 7 (cationic amino acid tra	1421093_at	up	3,031173
Pcolce2	76477	procollagen C-endopeptidase enhancer 2	1451527_at	up	3,0183113
Gda	14544	guanine deaminase	1422868_s_at	up	3,015623
D4Ertd103e	52225	DNA segment, Chr 4, ERATO Doi 103, expres:	1446823_at	up	3,0103486
Epha3	13837	Eph receptor A3	1425574_at	up	3,0079312
St6galnac5	26938	ST6 (alpha-N-acetyl-neuraminyl-2,3-beta-gal	1419420_at	up	3,0051622
2900046F13Rik	73028	RIKEN cDNA 2900046F13 gene	1454388_at	up	3,0007484
Gstm1	14862	glutathione S-transferase, mu 1	1416416_x_at	up	3,0000951
6330406I15Rik	70717	RIKEN cDNA 6330406I15 gene	1426937_at	up	2,9992757
Mär.03	320253	membrane-associated ring finger (C3HC4) 3	1430918_at	up	2,990196
Kitl	17311	kit ligand	1448117_at	up	2,9896505
Cp	12870	ceruloplasmin	1417496_at	up	2,9878387
Wisp2	22403	WNT1 inducible signaling pathway protein 2	1419015_at	up	2,9857183
5730410E15Rik	319613	RIKEN cDNA 5730410E15 gene	1438667_at	up	2,985575
Csf1	12977	colony stimulating factor 1 (macrophage)	1448914_a_at	up	2,9816747
			1436614_at	up	2,9731176
Fmod	14264	fibromodulin	1415939_at	up	2,9688444
Ldhb	16832	lactate dehydrogenase B	1448237_x_at	up	2,9659722
Plcl1	227120	phospholipase C-like 1	1443974_at	up	2,9640214
Kitl	17311	kit ligand	1415855_at	up	2,9637249
			1447609_at	up	2,961126
Ldhb	16832	lactate dehydrogenase B	1455235_x_at	up	2,9609396
Tll1	21892	tolloid-like	1420753_at	up	2,9576426
Cp	12870	ceruloplasmin	1417495_x_at	up	2,956571
Chsy3	78923	chondroitin sulfate synthase 3	1429417_at	up	2,9564142
Nov	18133	nephroblastoma overexpressed gene	1426852_x_at	up	2,9561706
Hcfc1r1	353502	host cell factor C1 regulator 1 (XPO1-depende	1428405_at	up	2,9551225
Kitl	17311	kit ligand	1415854_at	up	2,955045
Bend6	320705	BEN domain containing 6	1434590_at	up	2,9500809
Slc4a4	54403	solute carrier family 4 (anion exchanger), me	1434096_at	up	2,9471393
A530020G20Rik	319839	RIKEN cDNA A530020G20 gene	1444161_at	up	2,945125
Abcc9	20928	ATP-binding cassette, sub-family C (CFTR/MR	1435752_s_at	up	2,9442856
Ogn	18295	osteoglycin	1419663_at	up	2,9352007
Nov	18133	nephroblastoma overexpressed gene	1426851_a_at	up	2,9252706
2900046L07Rik	73027	RIKEN cDNA 2900046L07 gene	1432944_at	up	2,91948
			1445032_at	up	2,9194078
Slco2a1	24059	Solute carrier organic anion transporter famil	1444242_at	up	2,9170105
Tcf7l2	21416	transcription factor 7-like 2, T-cell specific, HI	1429427_s_at	up	2,91625
Glt8d2	74782	glycosyltransferase 8 domain containing 2	1429403_x_at	up	2,9135518
Rab3il1	74760	RAB3A interacting protein (rabin3)-like 1	1456442_at	up	2,8949947
Fmod	14264	fibromodulin	1437718_x_at	up	2,8936696
Ptgfr	19220	prostaglandin F receptor	1449828_at	up	2,8861766
1200002N14Rik	71712	RIKEN cDNA 1200002N14 gene	1424524_at	up	2,879142
Fmod	14264	fibromodulin	1437324_x_at	up	2,878072
Serpinf1	20317	serine (or cysteine) peptidase inhibitor, clade	1416168_at	up	2,8732116
Acot7	70025	acyl-CoA thioesterase 7	1417094_at	up	2,8722475
Fth1	14319	ferritin heavy chain 1	1427021_s_at	up	2,8708243

Atp1a2	98660	ATPase, Na+/K+ transporting, alpha 2 polype	1452308_a_at	up	2,8707504
Fos	14281	FBJ osteosarcoma oncogene	1423100_at	up	2,8682284
Gstm1	14862	glutathione S-transferase, mu 1	1448330_at	up	2,8660765
Slc38a4	69354	solute carrier family 38, member 4	1448889_at	up	2,8582819
Gstm1	14862	glutathione S-transferase, mu 1	1425627_x_at	up	2,8574846
Nmnat3	74080	nicotinamide nucleotide adenylyltransferase 3	1424899_at	up	2,8528845
1110002E22Rik	67816	RIKEN cDNA 1110002E22 gene	1447870_x_at	up	2,8498287
Thbs3	21827	thrombospondin 3	1416623_at	up	2,844096
Agt	11606	angiotensinogen (serpin peptidase inhibitor, c	1423396_at	up	2,8418825
Rasgrf1	19417	RAS protein-specific guanine nucleotide-relea	1422600_at	up	2,8365521
Icam1	15894	intercellular adhesion molecule 1	1424067_at	up	2,8361914
Fuca2	66848	fucosidase, alpha-L- 2, plasma	1416046_a_at	up	2,8361309
			1443545_at	up	2,8361042
Rnf144a	108089	ring finger protein 144A	1421243_at	up	2,8355246
Igf2	16002	insulin-like growth factor 2	1448152_at	up	2,8334653
Ldhb	16832	lactate dehydrogenase B	1416183_a_at	up	2,8313613
Gucy1a3	60596	guanylate cyclase 1, soluble, alpha 3	1434141_at	up	2,8232827
Lama4	16775	laminin, alpha 4	1424808_at	up	2,8212523
2900062L11Rik /// 65304(	76219 /// 769	RIKEN cDNA 2900062L11 gene /// RIKEN cDN	1428333_at	up	2,8178043
Tnfrsf23	79201	tumor necrosis factor receptor superfamily, m	1422101_at	up	2,817308
Abcd2	26874	ATP-binding cassette, sub-family D (ALD), me	1438431_at	up	2,8125045
Mmp3	17392	matrix metallopeptidase 3	1418945_at	up	2,8108034
Arhgap21	71435	Rho GTPase activating protein 21	1428369_s_at	up	2,8097713
Ugt1a1 /// Ugt1a10 /// Ug	22236 /// 394	UDP glucuronosyltransferase 1 family, polype	1426260_a_at	up	2,8089626
Atp1a2	98660	ATPase, Na+/K+ transporting, alpha 2 polype	1455136_at	up	2,8070002
Fmod	14264	fibromodulin	1437685_x_at	up	2,8065066
Ly86	17084	lymphocyte antigen 86	1422903_at	up	2,80255
Timp4	110595	tissue inhibitor of metalloproteinase 4	1423405_at	up	2,7959645
			1448021_at	up	2,7948978
Oasl2	23962	2'-5' oligoadenylate synthetase-like 2	1453196_a_at	up	2,790691
Ugt1a1 /// Ugt1a10 /// Ug	22236 /// 394	UDP glucuronosyltransferase 1 family, polype	1426261_s_at	up	2,785483
LOC640441 /// Thbs1	21825 /// 640	similar to thrombospondin 1 /// thrombospon	1450377_at	up	2,7842724
Nova1	664883	neuro-oncological ventral antigen 1	1452245_at	up	2,7829785
			1445574_at	up	2,7812672
Plcl1	227120	phospholipase C-like 1	1445723_at	up	2,7769847
Pdk4	27273	pyruvate dehydrogenase kinase, isoenzyme 4	1417273_at	up	2,7758176
C920025E04Rik /// H2-T2:	15040 /// 667	RIKEN cDNA C920025E04 gene /// histocomp	1449556_at	up	2,7741213
Plvap	84094	plasmalemma vesicle associated protein	1418090_at	up	2,773081
Col9a1	12839	collagen, type IX, alpha 1	1428571_at	up	2,7696385
A930017M01Rik	239410	RIKEN cDNA A930017M01 gene	1437076_at	up	2,7682357
LOC640441 /// Thbs1	21825 /// 640	similar to thrombospondin 1 /// thrombospon	1421811_at	up	2,7664826
Abca1	11303	ATP-binding cassette, sub-family A (ABC1), m	1450392_at	up	2,7549407
Hspa1a	193740	heat shock protein 1A	1452388_at	up	2,7546208
Irak3	73914	interleukin-1 receptor-associated kinase 3	1435040_at	up	2,7545972
Gnas	14683	GNAS (guanine nucleotide binding protein, al	1427789_s_at	up	2,7491105
Tcf7l2	21416	transcription factor 7-like 2, T-cell specific, HI	1425229_a_at	up	2,745274
Gda	14544	guanine deaminase	1435748_at	up	2,7431464
Thbs2	21826	thrombospondin 2	1422571_at	up	2,7427807
Rnase4	58809	ribonuclease, RNase A family 4	1422603_at	up	2,7407696
Mmp23	26561	matrix metallopeptidase 23	1417282_at	up	2,737936
Angptl4	57875	angiopoietin-like 4	1417130_s_at	up	2,7329693
Il1r1	16177	interleukin 1 receptor, type I	1448950_at	up	2,7309873
Hgf	15234	hepatocyte growth factor	1451866_a_at	up	2,7259493
			1447411_at	up	2,7231643
4921524M04Rik	70890	RIKEN cDNA 4921524M04 gene	1433098_at	up	2,7222111
D14Ertd449e /// ENSMUSC	100039192 ///	DNA segment, Chr 14, ERATO Doi 449, expre	1428738_a_at	up	2,7213345
Aspn	66695	asporin	1416652_at	up	2,7206342
Ntrk2	18212	neurotrophic tyrosine kinase, receptor, type 2	1420837_at	up	2,715012
Fosb	14282	FBJ osteosarcoma oncogene B	1422134_at	up	2,7103639
			1441846_x_at	up	2,7058907
Adamts12	239337	a disintegrin-like and metallopeptidase (repro	1439827_at	up	2,7052522
2810433D01Rik	77132	RIKEN cDNA 2810433D01 gene	1445896_at	up	2,7051356
Aass	30956	aminoadipate-semialdehyde synthase	1423523_at	up	2,7039466
Gfra2	14586	glial cell line derived neurotrophic factor famil	1423007_a_at	up	2,7025955
2310001H17Rik	76432	RIKEN cDNA 2310001H17 gene	1446096_at	up	2,7025304
Meox1	17285	mesenchyme homeobox 1	1417595_at	up	2,701504
Casp1	12362	caspase 1	1449265_at	up	2,6995926
Xaf1	327959	XIAP associated factor 1	1443698_at	up	2,6960752
Man1a	17155	mannosidase 1, alpha	1417111_at	up	2,6942232
Cgref1	68567	cell growth regulator with EF hand domain 1	1424528_at	up	2,6838915
			1442019_at	up	2,6822023
1110012D08Rik	73827	RIKEN cDNA 1110012D08 gene	1433820_a_at	up	2,6810052
Adamts12	239337	a disintegrin-like and metallopeptidase (repro	1446666_at	up	2,6771894
Slc16a12	240638	solute carrier family 16 (monocarboxylic acid	1434188_at	up	2,6766472

Casp4	12363	caspase 4, apoptosis-related cysteine peptida	1449591_at	up	2,6738894
Gm266	212539	gene model 266, (NCBI)	1436115_at	up	2,6714623
Sphkap	77629	SPHK1 interactor, AKAP domain containing	1454926_at	up	2,6648602
Ramp2	54409	receptor (calcitonin) activity modifying protei	1418187_at	up	2,6646466
Nfib	18028	nuclear factor I/B	1416293_at	up	2,66021
ORF63	224419	open reading frame 63	1451810_at	up	2,6592054
Fam82a1	381110	family with sequence similarity 82, member A	1438036_x_at	up	2,6445165
LOC100045567 /// Pnp1 //	100045567 //	similar to purine nucleoside phosphorylase //	1453299_a_at	up	2,6417017
			1441247_at	up	2,6404731
5033415L01Rik	75982	RIKEN cDNA 5033415L01 gene	1453905_at	up	2,6381958
Nfib	18028	nuclear factor I/B	1434101_at	up	2,638135
Ddc	13195	dopa decarboxylase	1426215_at	up	2,6366358
Ifi203	15950	interferon activated gene 203	1448775_at	up	2,6357753
Hspa1b	15511	heat shock protein 1B	1427126_at	up	2,6352746
Cxcl2	20310	chemokine (C-X-C motif) ligand 2	1449984_at	up	2,633199
AW551984	244810	expressed sequence AW551984	1433435_at	up	2,6178856
Rnf144a	108089	ring finger protein 144A	1421242_at	up	2,6158516
A130040M12Rik	319269	RIKEN cDNA A130040M12 gene	1428909_at	up	2,6152039
Lox	16948	lysyl oxidase	1448228_at	up	2,6125977
Lbp	16803	lipopolysaccharide binding protein	1448550_at	up	2,612395
Pcsk5	18552	proprotein convertase subtilisin/kexin type 5	1451406_a_at	up	2,607854
6030403N03Rik	402764	RIKEN cDNA 6030403N03 gene	1446341_at	up	2,6061478
Sec16b	89867	SEC16 homolog B (S. cerevisiae)	1450734_at	up	2,6045964
Ptx3	19288	pentraxin related gene	1418666_at	up	2,6028616
Abca1	11303	ATP-binding cassette, sub-family A (ABC1), m	1421840_at	up	2,5974047
Egfr	13649	epidermal growth factor receptor	1424932_at	up	2,595796
Gas7	14457	Growth arrest-specific 7-cb protein (Gas7-cb)	1457948_at	up	2,592006
Xdh	22436	xanthine dehydrogenase	1451006_at	up	2,5889578
Zim1	22776	zinc finger, imprinted 1	1421405_at	up	2,587855
Slc1a5	20514	solute carrier family 1 (neutral amino acid tra	1416629_at	up	2,58777
			1438084_at	up	2,586697
Htra3	78558	HtrA serine peptidase 3	1419292_at	up	2,584431
1110012D08Rik	73827	RIKEN cDNA 1110012D08 gene	1433821_at	up	2,5805821
Gas7	14457	growth arrest specific 7	1445685_at	up	2,5796995
Prkar2b	19088	protein kinase, cAMP dependent regulatory, t	1438664_at	up	2,5776832
Atf3	11910	activating transcription factor 3	1449363_at	up	2,5703475
Ppp1r3c	53412	protein phosphatase 1, regulatory (inhibitor) :	1433691_at	up	2,5690846
AI987986	105573	expressed sequence AI987986	1457717_at	up	2,5634387
Gpr81	243270	G protein-coupled receptor 81	1438411_at	up	2,560625
Pcolce	18542	procollagen C-endopeptidase enhancer protei	1437165_a_at	up	2,5584404
Ptn	19242	pleiotrophin	1416211_a_at	up	2,5584304
Abi3bp	320712	ABI gene family, member 3 (NESH) binding p	1427054_s_at	up	2,557425
Hspa1b	15511	heat shock protein 1B	1427127_x_at	up	2,5573165
Nenf	66208	neuron derived neurotrophic factor	1417879_at	up	2,5572443
Pcdh19	279653	protocadherin 19	1455145_at	up	2,554321
Khk	16548	ketohexokinase	1449062_at	up	2,5535727
Prdm8	77630	PR domain containing 8	1455925_at	up	2,5483823
Adm	11535	adrenomedullin	1416077_at	up	2,5473373
Fkbp11	66120	FK506 binding protein 11	1417267_s_at	up	2,5445087
6330406I15Rik	70717	RIKEN cDNA 6330406I15 gene	1452244_at	up	2,5415778
Kazald1	107250	Kazal-type serine peptidase inhibitor domain	1436528_at	up	2,538832
Nfib	18028	nuclear factor I/B	1454834_at	up	2,535415
			1458205_at	up	2,5343528
Irgm2	54396	immunity-related GTPase family M member 2	1417793_at	up	2,523436
Cmpk2	22169	cytidine monophosphate (UMP-CMP) kinase 2	1450484_a_at	up	2,523231
Tmem135	72759	transmembrane protein 135	1452074_at	up	2,522978
Hcfc1r1	353502	host cell factor C1 regulator 1 (XPO1-depende	1428406_s_at	up	2,5210845
Pear1	73182	platelet endothelial aggregation receptor 1	1425267_a_at	up	2,5201402
Ntrk2	18212	neurotrophic tyrosine kinase, receptor, type 2	1435196_at	up	2,5180175
Mrpl19	56284	mitochondrial ribosomal protein L19	1421913_at	up	2,5175056
Acyp2	75572	acylphosphatase 2, muscle type	1427943_at	up	2,5173123
Tmed3	66111	transmembrane emp24 domain containing 3	1416108_a_at	up	2,5163722
Ifitm3	66141	interferon induced transmembrane protein 3	1423754_at	up	2,5129678
Sema3c	20348	sema domain, immunoglobulin domain (Ig), s	1429348_at	up	2,5066144
Depdc6	97998	DEP domain containing 6	1453571_at	up	2,506455
Larp6	67557	La ribonucleoprotein domain family, member	1418400_at	up	2,5057406
Steap4	117167	STEAP family member 4	1460197_a_at	up	2,495369
			1438072_at	up	2,495058
Pcdh19	279653	protocadherin 19	1437360_at	up	2,4943714
Hmgcs2	15360	3-hydroxy-3-methylglutaryl-Coenzyme A synt	1431833_a_at	up	2,49325
Kcne4	57814	potassium voltage-gated channel, Isk-related	1418156_at	up	2,4868786
Kitl	17311	kit ligand	1426152_a_at	up	2,483331
Prokr1	58182	prokineticin receptor 1	1456543_at	up	2,4809694
Klf2	16598	Kruppel-like factor 2 (lung)	1448890_at	up	2,4806216

Lepr	16847	leptin receptor	1425644_at	up	2,4782407
Depdc6	97998	DEP domain containing 6	1443579_s_at	up	2,4777882
H2-M3	14991	histocompatibility 2, M region locus 3	1421358_at	up	2,477575
LOC100045567 /// Pnp1	100045567 ///	similar to purine nucleoside phosphorylase ///	1416530_a_at	up	2,4748628
Gsn	227753	gelsolin	1415812_at	up	2,4721017
Clec14a	66864	C-type lectin domain family 14, member a	1419468_at	up	2,4674513
Lox	16948	lysyl oxidase	1416121_at	up	2,4662285
Mtap9	213582	microtubule-associated protein 9	1440231_at	up	2,4660661
			1445148_at	up	2,463572
Egfr	13649	epidermal growth factor receptor	1435888_at	up	2,4617348
Hivep2	15273	human immunodeficiency virus type I enhanc	1422018_at	up	2,460385
Arhgap20	244867	Rho GTPase activating protein 20	1429918_at	up	2,4576564
Rnf112	22671	ring finger protein 112	1456108_x_at	up	2,4549558
Aspn	66695	asporin	1448421_s_at	up	2,4539967
Gaa	14387	glucosidase, alpha, acid	1419428_a_at	up	2,4493828
Nfib	18028	nuclear factor I/B	1438244_at	up	2,447772
Tnfrsf22 /// Tnfrsf23	79201 /// 792	tumor necrosis factor receptor superfamily, m	1442590_at	up	2,4469023
Icosl	50723	icos ligand	1419212_at	up	2,442437
Engase	217364	endo-beta-N-acetylglucosaminidase	1435289_at	up	2,4367757
Pde1a	18573	phosphodiesterase 1A, calmodulin-dependent	1449298_a_at	up	2,4346316
Smoc2	64074	SPARC related modular calcium binding 2	1415935_at	up	2,4336648
Vcan	13003	versican	1447887_x_at	up	2,4330413
Wfdc12	192200	WAP four-disulfide core domain 12	1449191_at	up	2,4313686
Depdc6	97998	DEP domain containing 6	1428622_at	up	2,430418
Lama4	16775	laminin, alpha 4	1424807_at	up	2,4292648
Ptger3	19218	prostaglandin E receptor 3 (subtype EP3)	1425251_at	up	2,427172
St6galnac5	26938	ST6 (alpha-N-acetyl-neuraminyl-2,3-beta-gal	1449468_at	up	2,425418
Bckdhb	12040	branched chain ketoacid dehydrogenase E1, b	1427153_at	up	2,4231129
Nespas	56802	neuroendocrine secretory protein antisense	1427788_at	up	2,4212496
Ermn	77767	ermin, ERM-like protein	1440902_at	up	2,420563
Fgfrl1 /// LOC100046239	100046239 ///	fibroblast growth factor receptor-like 1 /// sim	1447878_s_at	up	2,418922
Slco2a1	24059	solute carrier organic anion transporter family	1450032_at	up	2,4187517
Slc1a3	20512	solute carrier family 1 (glial high affinity gluta	1440491_at	up	2,417278
Entpd5	12499	ectonucleoside triphosphate diphosphohydrola	1417382_at	up	2,4159446
Klf9	16601	Kruppel-like factor 9	1422264_s_at	up	2,4145164
Sema3c	20348	sema domain, immunoglobulin domain (Ig), s	1420696_at	up	2,4114962
Sv2b	64176	synaptic vesicle glycoprotein 2 b	1434800_at	up	2,4103732
Hmgcs2	15360	3-hydroxy-3-methylglutaryl-Coenzyme A synt	1423858_a_at	up	2,4095645
Abcd2	26874	ATP-binding cassette, sub-family D (ALD), me	1419748_at	up	2,4082794
Tgfb2	21808	transforming growth factor, beta 2	1450922_a_at	up	2,4024951
Slc4a4	54403	solute carrier family 4 (anion exchanger), me	1421225_a_at	up	2,401222
Tmem106a	217203	transmembrane protein 106A	1425025_at	up	2,396434
Timp1	21857	tissue inhibitor of metalloproteinase 1	1460227_at	up	2,3943732
Ghr	14600	growth hormone receptor	1451871_a_at	up	2,3937414
			1442214_at	up	2,388129
Sectm1a	209588	secreted and transmembrane 1A	1425002_at	up	2,3868513
Mär.03	320253	membrane-associated ring finger (C3HC4) 3	1441643_at	up	2,3785892
Il15ra	16169	interleukin 15 receptor, alpha chain	1448681_at	up	2,3737195
Abca8a	217258	ATP-binding cassette, sub-family A (ABC1), m	1427371_at	up	2,3731577
			1456648_at	up	2,3718758
Amacr	17117	alpha-methylacyl-CoA racemase	1417208_at	up	2,3714266
Ifit3	15959	interferon-induced protein with tetratricopepti	1449025_at	up	2,3704524
LOC666466 /// Obox2	246792 /// 66	similar to OBOX2 /// oocyte specific homeobo	1456017_x_at	up	2,3685236
Naalad2	72560	N-acetylated alpha-linked acidic dipeptidase 2	1422671_s_at	up	2,3666
Oxsm	71147	3-oxoacyl-ACP synthase, mitochondrial	1455395_at	up	2,3646996
Vapb	56491	vesicle-associated membrane protein, associa	1423152_at	up	2,3646252
Ang	11727	angiogenin, ribonuclease, RNase A family, 5	1438936_s_at	up	2,3616178
Slco2b1	101488	solute carrier organic anion transporter family	1454777_at	up	2,3587134
Mest	17294	mesoderm specific transcript	1423294_at	up	2,352381
Arhgap20	244867	Rho GTPase activating protein 20	1427522_at	up	2,3484936
Rassf2	215653	Ras association (RalGDS/AF-6) domain family	1444889_at	up	2,345322
Ablim1	226251	actin-binding LIM protein 1	1454708_at	up	2,3441641
Adam12	11489	a disintegrin and metallopeptidase domain 12	1421171_at	up	2,342694
Erap1	80898	endoplasmic reticulum aminopeptidase 1	1416942_at	up	2,3392491
Shisa3	330096	shisa homolog 3 (Xenopus laevis)	1438884_at	up	2,3359833
D530031A16Rik	78724	RIKEN cDNA D530031A16 gene	1433311_at	up	2,3358765
Aebp1	11568	AE binding protein 1	1422514_at	up	2,3355575
Dnajc6	72685	DnaJ (Hsp40) homolog, subfamily C, member	1431216_s_at	up	2,3326147
Igfbp6	16012	insulin-like growth factor binding protein 6	1417933_at	up	2,328691
1810010H24Rik	69066	RIKEN cDNA 1810010H24 gene	1428809_at	up	2,3272815
Rasgrf1	19417	RAS protein-specific guanine nucleotide-relea	1435614_s_at	up	2,3248394
Cgref1	68567	cell growth regulator with EF hand domain 1	1424529_s_at	up	2,3234272
Svep1	64817	sushi, von Willebrand factor type A, EGF and	1419182_at	up	2,3234165
Met	17295	met proto-oncogene	1434447_at	up	2,3198059

Kctd12b	207474	potassium channel tetramerisation domain co	1442368_at	up	2,3177152
Tmem178	68027	transmembrane protein 178	1429175_at	up	2,317604
Smarca1	93761	SWI/SNF related, matrix associated, actin dep	1460292_a_at	up	2,316353
Kctd12	239217	potassium channel tetramerisation domain co	1434881_s_at	up	2,3161242
Pcsk6	18553	proprotein convertase subtilisin/kexin type 6	1426981_at	up	2,3152976
Fxc1	14356	fractured callus expressed transcript 1	1417916_a_at	up	2,3136692
Col12a1	12816	collagen, type XII, alpha 1	1427391_a_at	up	2,3136683
Hsd17b11	114664	hydroxysteroid (17-beta) dehydrogenase 11	1434642_at	up	2,3109844
Arhgap21	71435	Rho GTPase activating protein 21	1428368_at	up	2,3105352
Fbn1	14118	fibrillin 1	1438870_at	up	2,3102384
Fitm2	228859	fat storage-inducing transmembrane protein 2	1454935_at	up	2,3070526
Zfhx3	11906	zinc finger homeobox 3	1453267_at	up	2,3065996
Gfra2	14586	glial cell line derived neurotrophic factor famil	1425578_a_at	up	2,30473
Csgalnact1	234356	chondroitin sulfate N-acetylgalactosaminyltran	1452365_at	up	2,303972
Cdh26	381409	cadherin-like 26	1456937_at	up	2,301304
Ar	11835	androgen receptor	1455647_at	up	2,295866
Col14a1	12818	collagen, type XIV, alpha 1	1427168_a_at	up	2,2934582
Clec14a	66864	C-type lectin domain family 14, member a	1419467_at	up	2,2926245
Mmp23	26561	matrix metallopeptidase 23	1417281_a_at	up	2,29155
Neurl1a	18011	neuralized homolog 1A (Drosophila)	1456854_at	up	2,2902424
			1446738_at	up	2,289112
Ttc39b	69863	tetratricopeptide repeat domain 39B	1440192_at	up	2,2870643
Susd2	71733	sushi domain containing 2	1426511_at	up	2,2856598
Sigmar1	18391	sigma non-opioid intracellular receptor 1	1416750_at	up	2,2855716
Enpp2	18606	ectonucleotide pyrophosphatase/phosphodies	1448136_at	up	2,2843342
Pltp	18830	phospholipid transfer protein	1417963_at	up	2,2837255
Pdgfrl	68797	platelet-derived growth factor receptor-like	1428896_at	up	2,282032
Sv2b	64176	synaptic vesicle glycoprotein 2 b	1435687_at	up	2,2780545
Ang	11727	angiogenin, ribonuclease, RNase A family, 5	1450717_at	up	2,277357
			1438245_at	up	2,2770379
Srpx2	68792	sushi-repeat-containing protein, X-linked 2	1427919_at	up	2,2757225
Lmbr1	56873	limb region 1	1442371_at	up	2,2755938
Rtn4rl1	237847	reticulon 4 receptor-like 1	1455664_at	up	2,274735
Abhd5	67469	abhydrolase domain containing 5	1417565_at	up	2,2726643
Ttc39b	69863	tetratricopeptide repeat domain 39B	1452008_at	up	2,2676642
Ugt1a1 /// Ugt1a10 /// Ug	22236 /// 394	UDP glucuronosyltransferase 1 family, polype	1424783_a_at	up	2,267597
			1442026_at	up	2,2661285
Glt8d2	74782	glycosyltransferase 8 domain containing 2	1429402_at	up	2,2637641
Angptl1	72713	angiopoietin-like 1	1421421_at	up	2,2597983
Rtn2	20167	reticulon 2 (Z-band associated protein)	1419056_at	up	2,258783
Nfib	18028	nuclear factor I/B	1434102_at	up	2,2586157
Vkorc1	27973	vitamin K epoxide reductase complex, subunit	1452770_at	up	2,2579803
Klhl29	208439	kelch-like 29 (Drosophila)	1434639_at	up	2,255471
			1440006_at	up	2,25377
Irak3	73914	interleukin-1 receptor-associated kinase 3	1430704_at	up	2,2530284
P4ha3	320452	procollagen-proline, 2-oxoglutarate 4-dioxyge	1446951_at	up	2,252355
Cry2	12953	cryptochrome 2 (photolyase-like)	1426383_at	up	2,252129
S100a16	67860	S100 calcium binding protein A16	1447676_x_at	up	2,2516794
Bicc1	83675	bicaudal C homolog 1 (Drosophila)	1441137_at	up	2,2511828
Zfp462	242466	zinc finger protein 462	1456789_at	up	2,2510042
Elmo2	140579	engulfment and cell motility 2, ced-12 homolo	1436011_at	up	2,2472053
Ppp1r3c	53412	protein phosphatase 1, regulatory (inhibitor) s	1425631_at	up	2,2471309
Ghr	14600	growth hormone receptor	1417962_s_at	up	2,2457824
			1436697_at	up	2,24009
Gucy1a3	60596	guanylate cyclase 1, soluble, alpha 3	1420533_at	up	2,2400296
Il1rl2	107527	interleukin 1 receptor-like 2	1434903_s_at	up	2,2392106
Gbp3	55932	guanylate binding protein 3	1418392_a_at	up	2,2366235
Adamtsl4	229595	ADAMTS-like 4	1451932_a_at	up	2,2321792
Scara3	219151	scavenger receptor class A, member 3	1427020_at	up	2,2304997
Clec11a	20256	C-type lectin domain family 11, member a	1418796_at	up	2,2285898
Lpar4	78134	lysophosphatidic acid receptor 4	1439665_at	up	2,2279236
Tmem173	72512	transmembrane protein 173	1447621_s_at	up	2,2265863
Slc1a3	20512	solute carrier family 1 (glial high affinity gluta	1426341_at	up	2,2248466
Smoc2	64074	SPARC related modular calcium binding 2	1431362_a_at	up	2,2229526
Scg3	20255	secretogranin III	1448628_at	up	2,2221677
Rsad2	58185	radical S-adenosyl methionine domain contair	1436058_at	up	2,2198434
Nfkbiz	80859	nuclear factor of kappa light polypeptide gene	1417483_at	up	2,2175415
Arhgap29	214137	Rho GTPase activating protein 29	1444512_at	up	2,2144082
Loxl1	16949	lysyl oxidase-like 1	1451978_at	up	2,2138236
LOC100047606 /// Ntrk3	100047606 ///	similar to neurotrophic tyrosine kinase, recep	1422329_a_at	up	2,2131598
2310039H08Rik	67101	RIKEN cDNA 2310039H08 gene	1417348_at	up	2,2128596
Gsto1	14873	glutathione S-transferase omega 1	1456036_x_at	up	2,2125075
Csf1	12977	colony stimulating factor 1 (macrophage)	1425155_x_at	up	2,2118797
Csgalnact1	234356	chondroitin sulfate N-acetylgalactosaminyltran	1452366_at	up	2,2118487

Abhd14b	76491	abhydrolase domain containing 14b	1451326_at	up	2,2088397
Btg2	12227	B-cell translocation gene 2, anti-proliferative	1448272_at	up	2,2054703
Fabp4	11770	fatty acid binding protein 4, adipocyte	1424155_at	up	2,2023513
Ar	11835	androgen receptor	1437064_at	up	2,2022667
Map3k5	26408	mitogen-activated protein kinase kinase kinas	1439830_at	up	2,1993523
Pla2r1	18779	phospholipase A2 receptor 1	1450144_at	up	2,1946063
			1423435_at	up	2,192574
Thbs2	21826	thrombospondin 2	1447862_x_at	up	2,1918106
Tmem107	66910	transmembrane protein 107	1429058_at	up	2,1916566
Fibin	67606	fin bud initiation factor homolog (zebrafish)	1419376_at	up	2,190497
Igtp	16145	interferon gamma induced GTPase	1417141_at	up	2,18968
Tcf7l2	21416	transcription factor 7-like 2, T-cell specific, HI	1426639_a_at	up	2,185422
Col14a1	12818	collagen, type XIV, alpha 1	1453931_at	up	2,1853142
Nucb2	53322	nucleobindin 2	1418355_at	up	2,1804714
Tmem97	69071	transmembrane protein 97	1416376_at	up	2,179775
Smtnl2	276829	smoothelin-like 2	1455794_at	up	2,1762314
Itgb5	16419	integrin beta 5	1456195_x_at	up	2,175745
D9Ertd720e	52145	DNA segment, Chr 9, ERATO Doi 720, expres	1457776_at	up	2,1746588
Angptl2	26360	angiopoietin-like 2	1421002_at	up	2,1743908
Arsa	11883	arylsulfatase A	1460346_at	up	2,1712599
C3	12266	complement component 3	1423954_at	up	2,1710334
Derl1	67819	Der1-like domain family, member 1	1423082_at	up	2,1692085
Pla2g16	225845	phospholipase A2, group XVI	1451611_at	up	2,167748
Akap7	432442	A kinase (PRKA) anchor protein 7	1433905_at	up	2,166673
Fmo5 /// LOC100046051	100046051 ///	flavin containing monooxygenase 5 /// similar	1450332_s_at	up	2,166416
Ptges	64292	prostaglandin E synthase	1439747_at	up	2,1663249
Hspa1b	15511	heat shock protein 1B	1452318_a_at	up	2,164634
Ugdh	22235	UDP-glucose dehydrogenase	1416308_at	up	2,1644487
Sdpr	20324	serum deprivation response	1416778_at	up	2,1631281
Tspan18	241556	tetraspanin 18	1442174_at	up	2,162686
Creg1	433375	cellular repressor of E1A-stimulated genes 1	1415948_at	up	2,1624777
Gfra2	14586	glial cell line derived neurotrophic factor famil	1459847_x_at	up	2,159782
Il1r2	16178	interleukin 1 receptor, type II	1419532_at	up	2,156554
Pim3	223775	proviral integration site 3	1437100_x_at	up	2,1548338
Abi3bp	320712	ABI gene family, member 3 (NESH) binding p	1427053_at	up	2,1530879
Pycr1	209027	pyrroline-5-carboxylate reductase 1	1424556_at	up	2,1524446
			1443852_at	up	2,151211
Slc5a5	114479	solute carrier family 5 (sodium iodide sympor	1436239_at	up	2,1496994
Abca1	11303	ATP-binding cassette, sub-family A (ABC1), m	1421839_at	up	2,1492865
Car3	12350	carbonic anhydrase 3	1430584_s_at	up	2,1485937
Gfra2	14586	glial cell line derived neurotrophic factor famil	1433716_x_at	up	2,1485233
C85319	97983	expressed sequence C85319	1447267_at	up	2,1464357
1700025K23Rik	66337	RIKEN cDNA 1700025K23 gene	1460553_at	up	2,1446497
Ube2l6	56791	ubiquitin-conjugating enzyme E2L 6	1417172_at	up	2,1433878
Cdc42ep2	104252	CDC42 effector protein (Rho GTPase binding)	1428750_at	up	2,1429763
Sepp1	20363	selenoprotein P, plasma, 1	1452141_a_at	up	2,1384187
100038993 /// Il11ra1 ///	100038993 ///	predicted gene, 100038993 /// interleukin 11	1417505_s_at	up	2,137587
Apod	11815	apolipoprotein D	1416371_at	up	2,1375008
Foxo1	56458	forkhead box O1	1416983_s_at	up	2,1338353
Retsat	67442	retinol saturase (all trans retinol 13,14 reduct	1424716_at	up	2,1336899
Arhgap29	214137	Rho GTPase activating protein 29	1454745_at	up	2,1323628
Acaa1a /// Acaa1b	113868 /// 23	acetyl-Coenzyme A acyltransferase 1A /// ace	1416946_a_at	up	2,130093
Htra3	78558	HtrA serine peptidase 3	1427029_at	up	2,1286478
Flywch2	76917	FLYWCH family member 2	1438982_s_at	up	2,1285608
Hp	15439	haptoglobin	1448881_at	up	2,1283066
Dda1	66498	DET1 and DDB1 associated 1	1429038_at	up	2,1272013
Mfap4	76293	microfibrillar-associated protein 4	1424010_at	up	2,1241534
Lysmd2	70082	LysM, putative peptidoglycan-binding, domair	1428626_at	up	2,1234155
Kera	16545	keratocan	1418063_at	up	2,1232023
Aig1	66253	androgen-induced 1	1420679_a_at	up	2,1230292
Il33	77125	interleukin 33	1416200_at	up	2,1229758
Sgms2	74442	sphingomyelin synthase 2	1428663_at	up	2,1225216
Rgl1	19731	ral guanine nucleotide dissociation stimulator,	1449124_at	up	2,1224513
St3gal1	20442	ST3 beta-galactoside alpha-2,3-sialyltransfera	1418946_at	up	2,1197107
Mapk8ip1	19099	mitogen-activated protein kinase 8 interacting	1440619_at	up	2,1155026
Hebp1	15199	heme binding protein 1	1418172_at	up	2,115322
Fcgrt	14132	Fc receptor, IgG, alpha chain transporter	1416978_at	up	2,114841
Gas7	14457	growth arrest specific 7	1431400_a_at	up	2,1146493
Gm715	279618	gene model 715, (NCBI)	1445503_at	up	2,112715
Lrrc61	243371	leucine rich repeat containing 61	1423843_at	up	2,105327
Slc16a10	72472	solute carrier family 16 (monocarboxylic acid	1436368_at	up	2,1052356
Gpx7	67305	glutathione peroxidase 7	1417836_at	up	2,1033463
Cited2	17684	Cbp/p300-interacting transactivator, with Glu	1452207_at	up	2,1032317
Gm22	195209	gene model 22, (NCBI)	1459622_at	up	2,1018388

Ier3	15937	immediate early response 3	1419647_a_at	up	2,1011229
S100a16	67860	S100 calcium binding protein A16	1425560_a_at	up	2,099613
Cugbp2	14007	CUG triplet repeat, RNA binding protein 2	1439777_at	up	2,0979488
Hspb8	80888	heat shock protein 8	1417013_at	up	2,0966372
Tmem140	68487	transmembrane protein 140	1424354_at	up	2,0955524
Ell2	192657	elongation factor RNA polymerase II 2	1450744_at	up	2,0927207
Pcsk5	18552	proprotein convertase subtilisin/kexin type 5	1424605_at	up	2,0921168
Mpa2l	100702	macrophage activation 2 like	1447927_at	up	2,0914454
Txnip	56338	thioredoxin interacting protein	1415996_at	up	2,0898254
Shisa3	330096	shisa homolog 3 (Xenopus laevis)	1460000_at	up	2,089582
Itgb5	16419	integrin beta 5	1417533_a_at	up	2,0859463
Echdc2	52430	enoyl Coenzyme A hydratase domain containi	1425788_a_at	up	2,084781
Gas7	14457	growth arrest specific 7	1457270_at	up	2,0840957
C1qtnf6	72709	C1q and tumor necrosis factor related protein	1431856_a_at	up	2,0800202
Lmbr1	56873	limb region 1	1426272_at	up	2,0793912
Arsb	11881	arylsulfatase B	1429189_at	up	2,07674
Il6st	16195	interleukin 6 signal transducer	1452843_at	up	2,0761027
Pcsk5	18552	proprotein convertase subtilisin/kexin type 5	1437339_s_at	up	2,0738323
Dapp1	26377	dual adaptor for phosphotyrosine and 3-phos	1421936_at	up	2,072854
Car5b	56078	carbonic anhydrase 5b, mitochondrial	1419705_at	up	2,0697057
Hint3	66847	histidine triad nucleotide binding protein 3	1418583_at	up	2,0647027
Slc44a1	100434	solute carrier family 44, member 1	1423865_at	up	2,063538
2210403K04Rik	67098	RIKEN cDNA 2210403K04 gene	1428562_at	up	2,0625873
Gk5	235533	glycerol kinase 5 (putative)	1436210_at	up	2,061971
Ctbs	74245	chitobiase, di-N-acetyl-	1452504_s_at	up	2,061387
St3gal2	20444	ST3 beta-galactoside alpha-2,3-sialyltransfer	1421892_at	up	2,0608666
Rhoc	11853	ras homolog gene family, member C	1448605_at	up	2,0594952
			1445126_at	up	2,0579598
Srxn1	76650	Sulfiredoxin 1 homolog (S. cerevisiae) (Srxn1	1419942_at	up	2,057346
5430435G22Rik	226421	RIKEN cDNA 5430435G22 gene	1424987_at	up	2,0557687
Col12a1	12816	collagen, type XII, alpha 1	1434411_at	up	2,0553148
BC028528	229600	cDNA sequence BC028528	1427996_at	up	2,0548937
Nedd9	18003	neural precursor cell expressed, development	1422818_at	up	2,0541954
4833438C02Rik	107243	RIKEN cDNA 4833438C02 gene	1436524_at	up	2,052951
Tnfaip2	21928	tumor necrosis factor, alpha-induced protein	1438855_x_at	up	2,0525901
Atf5	107503	activating transcription factor 5	1425927_a_at	up	2,05241
Id2	15902	inhibitor of DNA binding 2	1422537_a_at	up	2,0507348
Casp12	12364	caspase 12	1449297_at	up	2,0478518
Galnt12	230145	UDP-N-acetyl-alpha-D-galactosamine:polype	1437760_at	up	2,0476525
Tlr2	24088	toll-like receptor 2	1419132_at	up	2,0461113
Ccdc80	67896	coiled-coil domain containing 80	1424186_at	up	2,0449371
EG331392	331392	predicted gene, EG331392	1445504_at	up	2,043128
Angptl2	26360	angiopoietin-like 2	1450085_at	up	2,0420258
Penk	18619	preproenkephalin	1427038_at	up	2,0412729
Scube2	56788	signal peptide, CUB domain, EGF-like 2	1453486_a_at	up	2,0403678
Fam46a	212943	family with sequence similarity 46, member A	1437868_at	up	2,0401835
Creg1	433375	cellular repressor of E1A-stimulated genes 1	1415947_at	up	2,039823
ENSMUSG00000053512	56501	predicted gene, ENSMUSG00000053512	1421337_at	up	2,0387561
Acvrl1	11482	activin A receptor, type II-like 1	1451604_a_at	up	2,03822
Magi3	99470	membrane associated guanylate kinase, WW	1421035_a_at	up	2,0381196
AW551984	244810	expressed sequence AW551984	1433434_at	up	2,0364494
D930003E18Rik	100048911	RIKEN cDNA D930003E18 gene	1440007_at	up	2,0362399
Lepr	16847	leptin receptor	1425875_a_at	up	2,0338757
Crtap	56693	cartilage associated protein	1448592_at	up	2,0334806
			1456897_at	up	2,0326333
Adamts9	101401	a disintegrin-like and metallopeptidase (repro	1437785_at	up	2,0325775
Galntl1	108760	UDP-N-acetyl-alpha-D-galactosamine:polype	1416760_at	up	2,031714
2010309E21Rik	66488	RIKEN cDNA 2010309E21 gene, mRNA (cDNA	1441796_at	up	2,0314357
Rai14	75646	retinoic acid induced 14	1417401_at	up	2,0304508
Ptges	64292	prostaglandin E synthase	1449450_at	up	2,0290911
Add3	27360	adducin 3 (gamma)	1426574_a_at	up	2,026838
Endog	13804	endonuclease G	1438317_a_at	up	2,026792
Slfn5	327978	schlafen 5	1458458_at	up	2,0258422
Nmi	64685	N-myc (and STAT) interactor	1425719_a_at	up	2,0258288
Tmed5	73130	transmembrane emp24 protein transport dom	1424574_at	up	2,0254087
Nnmt	18113	nicotinamide N-methyltransferase	1432517_a_at	up	2,0251353
Il15ra	16169	interleukin 15 receptor, alpha chain	1422397_a_at	up	2,0249681
Abhd4	105501	abhydrolase domain containing 4	1439259_x_at	up	2,0249343
Klf5	12224	Kruppel-like factor 5	1451021_a_at	up	2,0226882
Scn7a	20272	sodium channel, voltage-gated, type VII, alph	1427495_at	up	2,0226653
Clybl	69634	citrate lyase beta like	1421014_a_at	up	2,022483
AI843755	100215	expressed sequence AI843755	1457227_at	up	2,0217152
Adm	11535	adrenomedullin	1447839_x_at	up	2,0216103
Sirpa	19261	signal-regulatory protein alpha	1448534_at	up	2,020367

Pcdh19	279653	protocadherin 19	1444422_at	up	2,018448
Naaa	67111	N-acylethanolamine acid amidase	1452067_at	up	2,017922
Npc2	67963	Niemann Pick type C2	1416901_at	up	2,0177844
Aldoa	11674	aldolase A, fructose-bisphosphate	1433604_x_at	up	2,0168447
Tnfrsf22	79202	tumor necrosis factor receptor superfamily, m	1426095_a_at	up	2,0148747
1110014L15Rik	66127	RIKEN cDNA 1110014L15 gene	1460487_at	up	2,0133932
Arsb	11881	arylsulfatase B	1429190_at	up	2,0117178
Klf9	16601	Kruppel-like factor 9	1428288_at	up	2,0103245
Grb10	14783	growth factor receptor bound protein 10	1430164_a_at	up	2,0093162
Aldoa	11674	aldolase A, fructose-bisphosphate	1416921_x_at	up	2,0086687
Psap	19156	prosaposin	1415687_a_at	up	2,0083747
AU015536	101232	expressed sequence AU015536	1445025_at	up	2,007708
Prkar2b	19088	protein kinase, cAMP dependent regulatory, t	1456475_s_at	up	2,0057342
Ctsk	13038	cathepsin K	1450652_at	up	2,0048711
Mr1	15064	major histocompatibility complex, class I-rela	1421898_a_at	up	2,00474
Cd55	13136	CD55 antigen	1443906_at	up	2,0034251
Lrrc17	74511	leucine rich repeat containing 17	1429679_at	up	2,0026963
Enpp2	18606	ectonucleotide pyrophosphatase/phosphodies	1415894_at	up	2,0020313
Lgals4	16855	lectin, galactose binding, soluble 4	1451336_at	up	2,0015485
Retsat	67442	retinol saturase (all trans retinol 13,14 reduct	1424715_at	up	2,0010774
Inhba	16323	inhibin beta-A	1422053_at	down	24,612768
Clstn2	64085	calsyntenin 2	1441165_s_at	down	21,766167
Prdm1	12142	PR domain containing 1, with ZNF domain	1420425_at	down	13,858948
A930038C07Rik	68169	RIKEN cDNA A930038C07 gene	1453225_at	down	13,642835
Hes5	15208	hairy and enhancer of split 5 (Drosophila)	1456010_x_at	down	13,106865
1110064A23Rik	68893	RIKEN cDNA 1110064A23 gene	1439543_at	down	12,067304
Lrrn1	16979	leucine rich repeat protein 1, neuronal	1416053_at	down	11,859629
Ephb1	270190	Eph receptor B1	1455188_at	down	11,110536
Tcfap2c	21420	transcription factor AP-2, gamma	1418147_at	down	10,916606
Dio2	13371	deiodinase, iodothyronine, type II	1418937_at	down	10,782692
Tcfap2c	21420	transcription factor AP-2, gamma	1448977_at	down	10,457016
Prokr2	246313	prokineticin receptor 2	1437695_at	down	10,420709
Prom1	19126	prominin 1	1419700_a_at	down	10,127229
A930038C07Rik	68169	RIKEN cDNA A930038C07 gene	1460465_at	down	9,490845
Corin	53419	corin	1419017_at	down	9,416158
Prex2	109294	phosphatidylinositol-3,4,5-trisphosphate-depe	1432047_at	down	9,008382
Coch	12810	coagulation factor C homolog (Limulus polyph	1423285_at	down	8,56802
			1443161_at	down	8,537113
Tcfap2c	21420	transcription factor AP-2, gamma	1436392_s_at	down	8,51615
Cxcr4	12767	chemokine (C-X-C motif) receptor 4	1448710_at	down	7,737871
5330433J24Rik	78276	RIKEN cDNA 5330433J24 gene	1432935_at	down	7,622249
			1440026_at	down	7,565159
			1445869_at	down	7,561933
			1431225_at	down	7,4960485
Foxd1	15229	forkhead box D1	1418876_at	down	7,3359303
			1441333_at	down	7,2411904
Dll1	13388	delta-like 1 (Drosophila)	1419204_at	down	7,2115498
Apcdd1	494504	Adenomatosis polyposis coli down-regulated 1	1443639_at	down	7,0736623
Frzb	20378	frizzled-related protein	1416658_at	down	7,049022
Sox11	20666	SRY-box containing gene 11	1436790_a_at	down	6,9978485
Sox11	20666	SRY-box containing gene 11	1453125_at	down	6,9200845
Rspo3	72780	R-spondin 3 homolog (Xenopus laevis)	1455607_at	down	6,913683
Prokr2	246313	prokineticin receptor 2	1440564_at	down	6,832094
Adamts20	223838	a disintegrin-like and metallopeptidase (repro	1456901_at	down	6,7124095
Col23a1	237759	collagen, type XXIII, alpha 1	1429209_at	down	6,7111096
			1440052_at	down	6,5891137
			1456983_at	down	6,5865607
Trps1	83925	trichorhinophalangeal syndrome I (human)	1457445_at	down	6,579196
C530008M17Rik	320827	RIKEN cDNA C530008M17 gene	1434645_at	down	6,533178
5330433J24Rik	78276	RIKEN cDNA 5330433J24 gene	1432936_at	down	6,500406
Dab1	13131	disabled homolog 1 (Drosophila)	1435577_at	down	6,454861
			1458328_x_at	down	6,4470425
Fbp1	14121	fructose bisphosphatase 1	1448470_at	down	6,2359433
Hey1	15213	hairy/enhancer-of-split related with YRPW mo	1415999_at	down	6,225097
Ednrb	13618	endothelin receptor type B	1437347_at	down	6,192658
Fgfr4	14186	fibroblast growth factor receptor 4	1418596_at	down	6,1750507
Slc1a1	20510	solute carrier family 1 (neuronal/epithelial hig	1425415_a_at	down	6,0274806
Col23a1	237759	collagen, type XXIII, alpha 1	1440911_at	down	6,0058417
			1436279_at	down	5,9901533
Lamc3	23928	laminin gamma 3	1451758_at	down	5,9655075
Spock1	20745	sparc/osteonectin, cwcv and kazal-like domai	1419673_at	down	5,9604
Dab1	13131	disabled homolog 1 (Drosophila)	1435578_s_at	down	5,948686
Slain1	105439	SLAIN motif family, member 1	1424824_at	down	5,8723917
Kcnj3	16519	potassium inwardly-rectifying channel, subfar	1455374_at	down	5,8590946

Frzb	20378	frizzled-related protein	1448424_at	down	5,7555947
Lmo7	380928	LIM domain only 7	1455056_at	down	5,596173
Entpd1	12495	ectonucleoside triphosphate diphosphohydrola	1450939_at	down	5,5743723
3222402N08Rik	74021	RIKEN cDNA 3222402N08 gene	1432656_at	down	5,5332184
Sgip1	73094	SH3-domain GRB2-like (endophilin) interactin	1425180_at	down	5,5281777
Bex1	19716	brain expressed gene 1	1448595_a_at	down	5,5275464
Itga4	16401	integrin alpha 4	1450155_at	down	5,5256295
Sgip1	73094	SH3-domain GRB2-like (endophilin) interactin	1431300_at	down	5,461338
Sox11	20666	SRY-box containing gene 11	1453002_at	down	5,397319
			1440563_at	down	5,3810515
4930426D05Rik	74644	RIKEN cDNA 4930426D05 gene	1430143_at	down	5,326826
Slitrk6	239250	SLIT and NTRK-like family, member 6	1437231_at	down	5,3244095
Itga4	16401	integrin alpha 4	1421194_at	down	5,3044376
Rarres1	109222	retinoic acid receptor responder (tazarotene i	1438055_at	down	5,1808944
Dio2	13371	deiodinase, iodothyronine, type II	1418938_at	down	5,170079
Trps1	83925	trichorhinophalangeal syndrome I (human)	1449530_at	down	5,129422
Hey2	15214	hairy/enhancer-of-split related with YRPW mo	1418106_at	down	5,1278424
			1446603_at	down	5,1232576
Dusp4	319520	dual specificity phosphatase 4	1428834_at	down	5,1175804
Prex2	109294	phosphatidylinositol-3,4,5-trisphosphate-depe	1436569_at	down	5,018501
3222402N08Rik	74021	RIKEN cDNA 3222402N08 gene	1432655_at	down	4,969268
Lef1	16842	lymphoid enhancer binding factor 1	1421299_a_at	down	4,952821
Slc26a7	208890	solute carrier family 26, member 7	1425841_at	down	4,9147735
3110005L21Rik	73104	RIKEN cDNA 3110005L21 gene	1453646_at	down	4,8987074
Trps1	83925	trichorhinophalangeal syndrome I (human)	1438214_at	down	4,8789186
Sox11	20666	SRY-box containing gene 11	1429372_at	down	4,869876
Wnt5a	22418	wingless-related MMTV integration site 5A	1436791_at	down	4,858532
			1436398_at	down	4,848789
Fgd5	232237	FYVE, RhoGEF and PH domain containing 5	1460578_at	down	4,823001
			1441375_at	down	4,822474
Wnt5a	22418	wingless-related MMTV integration site 5A	1448818_at	down	4,8201833
Ccbe1	320924	collagen and calcium binding EGF domains 1	1453918_at	down	4,7633357
Bmp7	12162	bone morphogenetic protein 7	1432410_a_at	down	4,7403517
Spon1	233744	spondin 1, (f-spondin) extracellular matrix pr	1451342_at	down	4,7073817
			1458947_at	down	4,603657
2610109H07Rik	70433	RIKEN cDNA 2610109H07 gene	1456158_at	down	4,5898447
Megf6	230971	multiple EGF-like-domains 6	1437058_at	down	4,575191
Efnb2	13642	ephrin B2	1449548_at	down	4,5531874
Col23a1	237759	collagen, type XXIII, alpha 1	1429210_at	down	4,535803
			1447891_at	down	4,479655
Itga8	241226	integrin alpha 8	1427489_at	down	4,4717517
Efnb2	13642	ephrin B2	1419638_at	down	4,464409
Bmp7	12162	bone morphogenetic protein 7	1418910_at	down	4,452212
Efnb2	13642	ephrin B2	1419639_at	down	4,440879
Itga8	241226	integrin alpha 8	1454966_at	down	4,4278326
Cnnm1	83674	cyclin M1	1443170_at	down	4,4044404
			1459695_at	down	4,2977657
			1446383_at	down	4,2976246
			1437673_at	down	4,290999
Spock1	20745	sparc/osteonectin, cwcv and kazal-like domai	1419672_at	down	4,269608
Speg	11790	SPEG complex locus	1448664_a_at	down	4,202552
Itga4	16401	integrin alpha 4	1436037_at	down	4,1982837
Lphn3	319387	latrophilin 3	1460440_at	down	4,1704826
Lef1	16842	lymphoid enhancer binding factor 1	1454734_at	down	4,1399627
Satb2	212712	special AT-rich sequence binding protein 2	1425904_at	down	4,1268163
Kif26b	269152	kinesin family member 26B	1440990_at	down	4,112708
			1460133_at	down	4,1111965
Mpped2	77015	metallophosphoesterase domain containing 2	1435285_at	down	4,104734
Shroom3	27428	shroom family member 3	1422629_s_at	down	4,092108
Cd24a	12484	CD24a antigen	1448182_a_at	down	4,0849314
Cd24a	12484	CD24a antigen	1416034_at	down	4,0678425
Cd24a	12484	CD24a antigen	1437502_x_at	down	4,058933
Fam134b	66270	family with sequence similarity 134, member	1424683_at	down	4,023445
			1446484_at	down	4,0143332
Etl4	208618	enhancer trap locus 4	1440064_at	down	4,008549
Prss23	76453	protease, serine, 23	1431057_a_at	down	3,9930344
ENSMUSG00000074303	100038489	predicted gene, ENSMUSG00000074303	1436287_at	down	3,9898689
Sox11	20666	SRY-box containing gene 11	1429051_s_at	down	3,989508
Entpd1	12495	ectonucleoside triphosphate diphosphohydrola	1453586_at	down	3,9760787
Ttyh1	57776	tweety homolog 1 (Drosophila)	1422694_at	down	3,9709218
Trps1	83925	trichorhinophalangeal syndrome I (human)	1434286_at	down	3,9692175
8430418B16Rik	71491	RIKEN cDNA 8430418B16 gene	1433130_at	down	3,9161096
Adamts18	208936	a disintegrin-like and metallopeptidase (repro	1446247_at	down	3,89882
Daam2	76441	dishevelled associated activator of morphoger	1455717_s_at	down	3,8847482

8030447M02Rik	399578	RIKEN cDNA 8030447M02 gene	1458601_at	down	3,8759959
			1446172_at	down	3,8284621
Mef2c	17260	myocyte enhancer factor 2C	1424852_at	down	3,8273158
Emid2	140709	EMI domain containing 2	1425310_a_at	down	3,8025134
Sox18	20672	SRY-box containing gene 18	1449135_at	down	3,781732
100041195 /// 100041874	100036568 ///	predicted gene, 100041195 /// predicted gen	1428301_at	down	3,7733335
C530014P21Rik	399604	RIKEN cDNA C530014P21 gene	1447510_at	down	3,7688124
Bmp4	12159	bone morphogenetic protein 4	1422912_at	down	3,756284
Prlr	19116	prolactin receptor	1448556_at	down	3,7403152
6430537K16Rik	320480	RIKEN cDNA 6430537K16 gene	1438878_at	down	3,7215025
Fry	320365	furry homolog (Drosophila)	1456480_at	down	3,7122905
LOC677224 /// Ubash3b	677224 /// 72	similar to RIKEN cDNA 2810457I06 /// ubiqui	1429475_at	down	3,7105694
2010111I01Rik	72061	RIKEN cDNA 2010111I01 gene	1435089_at	down	3,6873431
9330159F19Rik	212448	RIKEN cDNA 9330159F19 gene	1458379_at	down	3,669269
Sdr16c6	242286	short chain dehydrogenase/reductase family 1	1457025_at	down	3,652356
Satb2	212712	special AT-rich sequence binding protein 2	1427017_at	down	3,6483057
100041195 /// 100041874	100036568 ///	predicted gene, 100041195 /// predicted gen	1452731_x_at	down	3,6326046
Tspan7	21912	tetraspanin 7	1448737_at	down	3,6246936
Mllt3	70122	myeloid/lymphoid or mixed-lineage leukemia	1453622_s_at	down	3,6108937
Mpped2	77015	metallophosphoesterase domain containing 2	1431751_a_at	down	3,6107838
Ndp	17986	Norrie disease (pseudoglioma) (human)	1449251_at	down	3,6100245
Pdlim3	53318	PDZ and LIM domain 3	1449178_at	down	3,583547
			1451930_at	down	3,5691
C030003D03Rik	77220	RIKEN cDNA C030003D03 gene	1436304_at	down	3,5598605
			1443337_at	down	3,5598528
2010111I01Rik	72061	RIKEN cDNA 2010111I01 gene	1460695_a_at	down	3,5489194
Mef2c	17260	myocyte enhancer factor 2C	1451507_at	down	3,5357955
Hrc /// Tcfap2a	15464 /// 214	histidine rich calcium binding protein /// trans	1426048_s_at	down	3,524866
Dclk1	13175	doublecortin-like kinase 1	1451917_a_at	down	3,499963
Slco5a1	240726	solute carrier organic anion transporter family	1440874_at	down	3,4911792
Ablim3	319713	actin binding LIM protein family, member 3	1434013_at	down	3,481431
Hs3st3b1	54710	heparan sulfate (glucosamine) 3-O-sulfotrans	1433977_at	down	3,4714346
Col8a1	12837	collagen, type VIII, alpha 1	1418441_at	down	3,4628618
Dclk1	13175	doublecortin-like kinase 1	1435940_at	down	3,4576414
Pon2	330260	paraoxonase 2	1429020_at	down	3,4509447
			1446293_at	down	3,4455857
Crabp1	12903	cellular retinoic acid binding protein I	1448326_a_at	down	3,4377575
Nipsnap1	18082	4-nitrophenylphosphatase domain and non-ne	1456748_a_at	down	3,4325151
Clstn2	64085	calsyntenin 2	1422158_at	down	3,423178
Edaradd	171211	EDAR (ectodysplasin-A receptor)-associated c	1437800_at	down	3,4229245
			1442859_at	down	3,4014542
Cep135	381644	centrosomal protein 135	1444201_at	down	3,3945663
Prkch	18755	protein kinase C, eta	1422079_at	down	3,3938315
Wnt5a	22418	wingless-related MMTV integration site 5A	1456976_at	down	3,3922718
Mllt3	70122	myeloid/lymphoid or mixed-lineage leukemia	1429205_at	down	3,3907666
Col8a1	12837	collagen, type VIII, alpha 1	1455627_at	down	3,3888524
			1435479_at	down	3,3877387
Sgip1	73094	SH3-domain GRB2-like (endophilin) interactin	1444225_at	down	3,3858287
Efna5	13640	ephrin A5	1436866_at	down	3,3823822
Serpine2	20720	serine (or cysteine) peptidase inhibitor, clade	1416666_at	down	3,3659906
1110059G02Rik	68786	RIKEN cDNA 1110059G02 gene	1438288_x_at	down	3,3659568
Rapgef4	56508	Rap guanine nucleotide exchange factor (GEF	1425518_at	down	3,3651881
Dkk2	56811	dickkopf homolog 2 (Xenopus laevis)	1420512_at	down	3,3528092
Limch1	77569	LIM and calponin homology domains 1	1435106_at	down	3,3527777
Limch1	77569	LIM and calponin homology domains 1	1453802_at	down	3,3314316
			1459453_at	down	3,3259227
LOC677224	677224	similar to RIKEN cDNA 2810457I06	1435703_at	down	3,3223245
Slc25a27	74011	solute carrier family 25, member 27	1454230_a_at	down	3,3155212
Inpp4b	234515	inositol polyphosphate-4-phosphatase, type I	1457359_at	down	3,314044
			1444841_at	down	3,3060517
Sostdc1	66042	sclerostin domain containing 1	1449340_at	down	3,2953782
Btbd11	74007	BTB (POZ) domain containing 11	1428377_at	down	3,293124
Runx1	12394	runt related transcription factor 1	1422864_at	down	3,287046
			1434683_at	down	3,281955
Sorbs1	20411	sorbin and SH3 domain containing 1	1428471_at	down	3,2779431
Limch1	77569	LIM and calponin homology domains 1	1435321_at	down	3,2764692
X99384	27355	cDNA sequence X99384	1448134_at	down	3,273121
Dclk1	13175	doublecortin-like kinase 1	1423125_at	down	3,2634218
Bcl11a	14025	B-cell CLL/lymphoma 11A (zinc finger protein	1456632_at	down	3,2524757
Gfra1	14585	glial cell line derived neurotrophic factor famil	1439015_at	down	3,2518718
Mycn	18109	v-myc myelocytomatosis viral related oncoge	1417155_at	down	3,2372067
Nkd2	72293	naked cuticle 2 homolog (Drosophila)	1419466_at	down	3,2108319
Tmem204	407831	transmembrane protein 204	1436698_x_at	down	3,1935813
			1440069_at	down	3,1907682

Nkd2	72293	naked cuticle 2 homolog (Drosophila)	1434275_at	down	3,1880872
Kank4	242553	KN motif and ankyrin repeat domains 4	1436425_at	down	3,1856346
1110059G02Rik	68786	RIKEN cDNA 1110059G02 gene	1435987_x_at	down	3,180592
Chodl	246048	chondrolectin	1451440_at	down	3,1733556
Dclk1	13175	doublecortin-like kinase 1	1450863_a_at	down	3,1710317
Cnnm1	83674	cyclin M1	1441312_at	down	3,1705098
Mtss1	211401	metastasis suppressor 1	1424826_s_at	down	3,166502
Ezr	22350	ezrin	1450850_at	down	3,1616569
St8sia2	20450	ST8 alpha-N-acetyl-neuraminide alpha-2,8-sia	1420377_at	down	3,156094
Rspo1	192199	R-spondin homolog (Xenopus laevis)	1449319_at	down	3,1548786
Serpinb5	20724	serine (or cysteine) peptidase inhibitor, clade	1441941_x_at	down	3,1538353
LOC552901	552901	hypothetical LOC552901	1458505_at	down	3,148919
			1446395_at	down	3,1430042
Tmem117	320709	transmembrane protein 117	1433615_at	down	3,1404984
			1442632_at	down	3,1401694
			1459289_at	down	3,1394026
Cacna2d2	56808	calcium channel, voltage-dependent, alpha 2/	1450754_at	down	3,132764
Mtss1	211401	metastasis suppressor 1	1434036_at	down	3,1222258
D3Bwg0562e	229791	DNA segment, Chr 3, Brigham & Women's Ge	1427247_at	down	3,1094766
Rspo3	72780	R-spondin 3 homolog (Xenopus laevis), mRNA	1443187_at	down	3,1046932
A730089K16Rik	320411	RIKEN cDNA A730089K16 gene	1437263_at	down	3,1038442
Prss23	76453	protease, serine, 23	1446560_at	down	3,1037235
4930588G05Rik	78817	RIKEN cDNA 4930588G05 gene	1430175_at	down	3,097917
Spred1	114715	Spred-1	1445228_at	down	3,094401
Elovl4	83603	elongation of very long chain fatty acids (FEN	1451308_at	down	3,093332
Crabp2	12904	cellular retinoic acid binding protein II	1451191_at	down	3,0927272
Mef2c	17260	myocyte enhancer factor 2C	1421027_a_at	down	3,0922196
6720418B01Rik	77892	RIKEN cDNA 6720418B01 gene	1430448_at	down	3,0859406
Serpinb5	20724	serine (or cysteine) peptidase inhibitor, clade	1424623_at	down	3,0817635
Perp	64058	PERP, TP53 apoptosis effector	1416271_at	down	3,0797935
Akap2	11641	A kinase (PRKA) anchor protein 2	1455870_at	down	3,06605
Itga4	16401	integrin alpha 4	1456498_at	down	3,06423
D7Ertd715e	52480	DNA segment, Chr 7, ERATO Doi 715, express	1455087_at	down	3,0568333
			1445954_at	down	3,0515568
Mef2c	17260	myocyte enhancer factor 2C	1451506_at	down	3,0377862
Ebf4	228598	early B-cell factor 4	1435044_at	down	3,0365949
Tubb4	22153	tubulin, beta 4	1423221_at	down	3,0360806
Gpr123	52389	G protein-coupled receptor 123	1459750_s_at	down	3,034233
Robo1	19876	roundabout homolog 1 (Drosophila)	1427231_at	down	3,0227907
Zfp365	216049	zinc finger protein 365	1433583_at	down	3,019458
Glrb	14658	glycine receptor, beta subunit	1422504_at	down	3,0142612
Smoc1	64075	SPARC related modular calcium binding 1	1448321_at	down	3,011778
LOC100045106 /// Vstm2b	100045106 ///	hypothetical protein LOC100045106 /// V-set	1419118_at	down	3,0101628
Sox21	223227	SRY-box containing gene 21	1437059_at	down	3,0096796
Ngfr	18053	nerve growth factor receptor (TNFR superfam	1450177_at	down	3,0004706
Kif5c	16574	kinesin family member 5C	1455266_at	down	2,9973233
Rapgef4	56508	Rap guanine nucleotide exchange factor (GEF	1421622_a_at	down	2,9968548
2210408K08Rik	108743	RIKEN cDNA 2210408K08 gene	1437087_at	down	2,9943323
Robo2	268902	roundabout homolog 2 (Drosophila)	1458229_at	down	2,9863267
Rnf144b	218215	ring finger protein 144B	1439153_at	down	2,9818234
Efna5	13640	ephrin A5	1421796_a_at	down	2,9811304
Tub	22141	tubby candidate gene	1420925_at	down	2,9749398
Wisp1	22402	WNT1 inducible signaling pathway protein 1	1448594_at	down	2,9707313
Pof1b	69693	premature ovarian failure 1B	1427492_at	down	2,9617722
Fam155a	270028	family with sequence similarity 155, member	1435138_at	down	2,952587
Cacna2d3	12294	calcium channel, voltage-dependent, alpha2/d	1419225_at	down	2,9463243
B4galnt1	14421	beta-1,4-N-acetyl-galactosaminyl transferase	1418655_at	down	2,9444647
Arap2	212285	ArfGAP with RhoGAP domain, ankyrin repeat a	1436895_at	down	2,934761
1110059G02Rik	68786	RIKEN cDNA 1110059G02 gene	1436204_at	down	2,9312515
Spon1	233744	spondin 1, (f-spondin) extracellular matrix pro	1441226_at	down	2,925729
Lxn	17035	latexin	1416503_at	down	2,9213474
Apcdd1	494504	adenomatosis polyposis coli down-regulated 1	1418382_at	down	2,9196274
Tle4	21888	transducin-like enhancer of split 4, homolog c	1430384_at	down	2,9147658
Dclk1	13175	doublecortin-like kinase 1	1424271_at	down	2,9117265
Flrt1	396184	fibronectin leucine rich transmembrane protei	1443143_at	down	2,9111626
Pde7b	29863	CAMP specific phosphodiesterase 7B (PDE7B (	1445539_at	down	2,9081252
Robo1	19876	roundabout homolog 1 (Drosophila)	1457407_at	down	2,9043167
Myocd	214384	myocardin	1425978_at	down	2,9000034
Emid1	140703	EMI domain containing 1	1449581_at	down	2,8965204
Apcdd1	494504	adenomatosis polyposis coli down-regulated 1	1449070_x_at	down	2,8960326
Cd200	17470	CD200 antigen	1448788_at	down	2,8925877
			1459304_at	down	2,8857133
			1459389_at	down	2,8840916
C530014P21Rik	399604	RIKEN cDNA C530014P21 gene	1441577_at	down	2,882286

Plod2	26432	procollagen lysine, 2-oxoglutarate 5-dioxygen	1416687_at	down	2,8805656
Apcdd1	494504	adenomatosis polyposis coli down-regulated 1	1418383_at	down	2,8797998
			1440015_at	down	2,87798
Stmn2	20257	stathmin-like 2	1423281_at	down	2,877706
Fli1	14247	Friend leukemia integration 1	1433512_at	down	2,8769767
Plk2	20620	polo-like kinase 2 (Drosophila)	1427005_at	down	2,8668475
Lass6	241447	LAG1 homolog, ceramide synthase 6	1434418_at	down	2,8659105
Cytsb	432572	cytospin B	1458440_at	down	2,8647766
4833419E13Rik	74579	RIKEN cDNA 4833419E13 gene	1431480_at	down	2,8628824
Pgbd5	209966	piggyBac transposable element derived 5	1460570_at	down	2,842901
			1444227_at	down	2,8407512
2010011I20Rik	67017	RIKEN cDNA 2010011I20 gene	1451450_at	down	2,8404536
Lonrf2	381338	LON peptidase N-terminal domain and ring fir	1429965_at	down	2,8401444
Dkk1	13380	dickkopf homolog 1 (Xenopus laevis)	1458232_at	down	2,8398345
			1444904_at	down	2,8379772
Bcl11a	14025	B-cell CLL/lymphoma 11A (zinc finger protein	1453814_at	down	2,8341265
Aqp1	11826	aquaporin 1	1416203_at	down	2,824845
			1420287_at	down	2,8175924
Ucn2	171530	urocortin 2	1450607_s_at	down	2,8175087
Unc5b	107449	unc-5 homolog B (C. elegans)	1435110_at	down	2,8131213
4832441B07Rik	76861	RIKEN cDNA 4832441B07 gene	1429997_at	down	2,8071685
Kdr	16542	kinase insert domain protein receptor	1449379_at	down	2,8020391
			1457376_at	down	2,8018386
			1443174_at	down	2,7965443
Prlr	19116	prolactin receptor	1437397_at	down	2,7930083
2010011I20Rik	67017	RIKEN cDNA 2010011I20 gene	1424694_at	down	2,7916737
Fam174b	100038347	family with sequence similarity 174, member	1434273_at	down	2,7845373
Limch1	77569	LIM and calponin homology domains 1 (Limch	1440776_at	down	2,7822893
Zdhhc2	70546	zinc finger, DHHC domain containing 2	1452656_at	down	2,781918
Fli1	14247	Friend leukemia integration 1	1422024_at	down	2,776271
4930403O15Rik	73814	RIKEN cDNA 4930403O15 gene	1432251_at	down	2,768407
Nav2	78286	neuron navigator 2	1444780_at	down	2,768313
Dtx4	207521	deltex 4 homolog (Drosophila)	1455711_at	down	2,7650523
2610307P16Rik	72518	RIKEN cDNA 2610307P16 gene	1430083_at	down	2,7632208
Ralgps2	78255	Ral GEF with PH domain and SH3 binding mot	1431704_a_at	down	2,7626686
4732423E21Rik	106967	RIKEN cDNA 4732423E21 gene	1442903_at	down	2,758998
			1443364_at	down	2,755903
Capns2	69543	calpain, small subunit 2	1429067_at	down	2,7450783
Syne1	64009	synaptic nuclear envelope 1	1421545_a_at	down	2,739884
Ets1	23871	E26 avian leukemia oncogene 1, 5' domain	1452163_at	down	2,7369041
			1440587_at	down	2,7256353
Scube3	268935	signal peptide, CUB domain, EGF-like 3	1436002_at	down	2,7192953
			1427600_at	down	2,718383
Col13a1	12817	collagen, type XIII, alpha 1	1422866_at	down	2,716082
			1446682_at	down	2,7150257
B3gnt2	53625	UDP-GlcNAc:betaGal beta-1,3-N-acetylglucos	1450026_a_at	down	2,7141125
Cdh2	12558	cadherin 2	1418815_at	down	2,7128541
Dclk1	13175	doublecortin-like kinase 1	1424270_at	down	2,711567
Lamc3	23928	laminin gamma 3	1425594_at	down	2,711251
Dclk1	13175	doublecortin-like kinase 1	1451289_at	down	2,7104592
AY512938	791388	cDNA sequence AY512938	1458551_at	down	2,704252
			1445713_at	down	2,698844
Snap91	20616	synaptosomal-associated protein 91	1416688_at	down	2,694785
Runx3	12399	runt related transcription factor 3	1440275_at	down	2,686543
Vwa2	240675	von Willebrand factor A domain containing 2	1438567_at	down	2,6846986
Etl4	208618	enhancer trap locus 4	1426880_at	down	2,6802342
Mtss1	211401	metastasis suppressor 1	1440847_at	down	2,6792107
Psapl1	76943	prosaposin-like 1	1440186_s_at	down	2,675199
			1436218_at	down	2,6721058
Ccrl1	252837	chemokine (C-C motif) receptor-like 1	1426139_a_at	down	2,6606688
Rassf10	78748	Ras association (RalGDS/AF-6) domain family	1457140_s_at	down	2,6592638
Megf10	70417	multiple EGF-like-domains 10	1429841_at	down	2,6585882
			1458823_at	down	2,652342
Syne1	64009	synaptic nuclear envelope 1	1455493_at	down	2,651904
Sorbs1	20411	sorbin and SH3 domain containing 1	1436737_a_at	down	2,6471148
Adrbk2	320129	adrenergic receptor kinase, beta 2	1434450_s_at	down	2,6424506
Nav2	78286	neuron navigator 2	1435981_at	down	2,6417627
Bcl11a	14025	B-cell CLL/lymphoma 11A (zinc finger protein	1457072_at	down	2,6406693
6330403K07Rik	103712	RIKEN cDNA 6330403K07 gene	1426766_at	down	2,640151
Fryl	72313	furry homolog-like (Drosophila)	1458233_at	down	2,6388087
Ralgps2	78255	CDNA clone IMAGE:6828107	1428789_at	down	2,6355622
Rgnef	110596	Rho-guanine nucleotide exchange factor	1419458_at	down	2,6353016
Cpne5	240058	copine V	1442166_at	down	2,6318507
4833406M21Rik	74575	RIKEN cDNA 4833406M21 gene	1432686_at	down	2,6314144

Runx1	12394	runt related transcription factor 1	1422865_at	down	2,630435
1110059G02Rik	68786	RIKEN cDNA 1110059G02 gene	1436203_a_at	down	2,62672
Alpl	11647	alkaline phosphatase, liver/bone/kidney	1423611_at	down	2,6240716
Adrbk2	320129	adrenergic receptor kinase, beta 2	1440801_s_at	down	2,6214306
Scel	64929	sciellin	1422837_at	down	2,6180148
Trib2	217410	tribbles homolog 2 (Drosophila)	1426641_at	down	2,612232
C030046M01Rik	77476	RIKEN cDNA C030046M01 gene	1454565_at	down	2,6121697
Tcfap2a	21418	transcription factor AP-2, alpha	1421996_at	down	2,6120925
A130064L14Rik	320257	RIKEN cDNA A130064L14 gene	1444343_at	down	2,6108322
Arl4c /// LOC632433	320982 /// 63	ADP-ribosylation factor-like 4C /// similar to A	1436512_at	down	2,6079936
			1446721_at	down	2,6048443
Esrp1	207920	epithelial splicing regulatory protein 1	1454681_at	down	2,5977418
			1440121_at	down	2,596009
Rassf9	237504	Ras association (RalGDS/AF-6) domain family	1427942_at	down	2,5915484
			1442675_at	down	2,5893838
Meox2	17286	mesenchyme homeobox 2	1424234_s_at	down	2,5892103
Trib2	217410	tribbles homolog 2 (Drosophila)	1426640_s_at	down	2,5883665
Shisa2	219134	shisa homolog 2 (Xenopus laevis)	1423851_a_at	down	2,5878716
St8sia2	20450	ST8 alpha-N-acetyl-neuraminide alpha-2,8-sia	1449843_at	down	2,586979
			1420286_at	down	2,586974
Ccdc3	74186	coiled-coil domain containing 3	1428549_at	down	2,5757678
Tmem35	67564	transmembrane protein 35	1416710_at	down	2,5725722
Nkd1	93960	naked cuticle 1 homolog (Drosophila)	1429506_at	down	2,572057
			1440219_at	down	2,56573
A730054J21Rik	320862	RIKEN cDNA A730054J21 gene	1438531_at	down	2,5560725
Nhedc2	97086	Na+/H+ exchanger domain containing 2	1439995_at	down	2,5559669
Agrn	11603	agrin	1426670_at	down	2,5512607
Scrn1	69938	secernin 1	1428718_at	down	2,5509176
C030009J22Rik	320436	RIKEN cDNA C030009J22 gene	1458386_at	down	2,5421827
Dkk1	13380	dickkopf homolog 1 (Xenopus laevis)	1420360_at	down	2,5379176
Dab1	13131	disabled homolog 1 (Drosophila)	1427308_at	down	2,5368109
Plod2	26432	procollagen lysine, 2-oxoglutarate 5-dioxygen	1416686_at	down	2,535002
Col19a1	12823	collagen, type XIX, alpha 1	1430676_at	down	2,5346289
Zbtb20	56490	zinc finger and BTB domain containing 20	1443471_at	down	2,532204
C130090K23Rik	231293	RIKEN cDNA C130090K23 gene	1426268_at	down	2,5291626
			1458730_at	down	2,5282261
Col4a6	94216	collagen, type IV, alpha 6	1421007_at	down	2,5276017
			1442966_at	down	2,524462
Dmd	13405	dystrophin, muscular dystrophy	1417307_at	down	2,5244582
Daam2	76441	dishevelled associated activator of morphogen	1430247_at	down	2,521875
C630043F03Rik	68285	RIKEN cDNA C630043F03 gene	1433762_at	down	2,5133243
2610027H17Rik	71811	RIKEN cDNA 2610027H17 gene	1442445_at	down	2,510252
Lphn3	319387	latrophilin 3	1428523_at	down	2,505699
Zswim6	67263	Zinc finger, SWIM domain containing 6 (Zswi	1459722_at	down	2,501333
Gdpd1	66569	glycerophosphodiester phosphodiesterase dor	1424076_at	down	2,5009234
A230048O21Rik	320959	RIKEN cDNA A230048O21 gene	1441562_at	down	2,492521
Cblb	208650	Casitas B-lineage lymphoma b	1458469_at	down	2,4913714
Drp2	13497	dystrophin related protein 2	1440452_at	down	2,4882958
Spred1	114715	sprouty protein with EVH-1 domain 1, related	1423161_s_at	down	2,4856281
			1443026_at	down	2,4787664
AI481207	105188	expressed sequence AI481207	1444587_at	down	2,473709
Tanc1	66860	tetratricopeptide repeat, ankyrin repeat and c	1452714_at	down	2,4699593
Efnb2	13642	ephrin B2	1449549_at	down	2,4619884
Ubash3b	72828	ubiquitin associated and SH3 domain containi	1436805_at	down	2,4598985
Spred1	114715	sprouty protein with EVH-1 domain 1, related	1428777_at	down	2,4567564
Edn3	13616	endothelin 3	1438696_at	down	2,4557233
Glis3	226075	GLIS family zinc finger 3	1430353_at	down	2,4537642
Fryl	72313	furry homolog-like (Drosophila)	1427199_at	down	2,4519496
Ednrb	13618	endothelin receptor type B	1423594_a_at	down	2,4488652
C030003D03Rik	77220	RIKEN cDNA C030003D03 gene	1431116_at	down	2,4470992
Spint2	20733	serine protease inhibitor, Kunitz type 2	1438968_x_at	down	2,4463613
			1440446_at	down	2,4451454
Masp1	17174	mannan-binding lectin serine peptidase 1	1425985_s_at	down	2,4435573
A230048O21Rik	320959	RIKEN cDNA A230048O21 gene	1458051_at	down	2,44251
Sox9	20682	SRY-box containing gene 9	1451538_at	down	2,4415913
Chd7	320790	chromodomain helicase DNA binding protein 7	1448026_at	down	2,4414697
Hdac9	79221	histone deacetylase 9	1434572_at	down	2,4370043
6720401G13Rik	103012	RIKEN cDNA 6720401G13 gene	1435744_at	down	2,4369943
Tcf7	21414	transcription factor 7, T-cell specific	1433471_at	down	2,4325566
Lfng	16848	LFNG O-fucosylpeptide 3-beta-N-acetylglucos	1420643_at	down	2,4321642
2600014E21Rik	72134	RIKEN cDNA 2600014E21 gene	1430946_at	down	2,432108
9430018C23Rik	77267	RIKEN cDNA 9430018C23 gene	1454512_at	down	2,4317722
9430081I23Rik	320242	RIKEN cDNA 9430081I23 gene	1446088_at	down	2,429066
Tmtc1	387314	transmembrane and tetratricopeptide repeat (	1435261_at	down	2,4278579

5730409N24Rik	70485	RIKEN cDNA 5730409N24 gene	1430362_at	down	2,427774
Entpd2	12496	ectonucleoside triphosphate diphosphohydrola	1418259_a_at	down	2,42722
Plxnb1	235611	plexin B1	1435254_at	down	2,4210067
Zfp238	30928	zinc finger protein 238	1417010_at	down	2,4182632
Prmt8	381813	protein arginine N-methyltransferase 8	1435204_at	down	2,417676
9430087B13Rik	77437	RIKEN cDNA 9430087B13 gene	1433321_at	down	2,4145908
Dst	13518	dystonin	1421117_at	down	2,4128778
Emilin3	280635	elastin microfibril interfacer 3	1436965_at	down	2,4127362
Meox2	17286	mesenchyme homeobox 2	1424233_at	down	2,4103322
Gpc2	71951	glypican 2 (cerebroglycan)	1429293_at	down	2,4102075
			1446953_at	down	2,409081
Tcf4	21413	Transcription factor 4, mRNA (cDNA clone MG	1458201_at	down	2,4064906
Rassf3	192678	Ras association (RalGDS/AF-6) domain family	1448546_at	down	2,4047832
Mtus1	102103	mitochondrial tumor suppressor 1	1454824_s_at	down	2,4038467
Pde2a	207728	phosphodiesterase 2A, cGMP-stimulated	1447707_s_at	down	2,4023225
Zdhhc2	70546	zinc finger, DHHC domain containing 2	1452654_at	down	2,3999906
Arhgef5	54324	Rho guanine nucleotide exchange factor (GEF	1452304_a_at	down	2,399439
Sox4	20677	SRY-box containing gene 4	1433575_at	down	2,3949451
2900011L18Rik	77082	RIKEN cDNA 2900011L18 gene	1432758_at	down	2,393961
5330421C15Rik	78279	RIKEN cDNA 5330421C15 gene	1432920_at	down	2,3920918
Myo10	17909	myosin X	1422544_at	down	2,387685
Btbd11	74007	BTB (POZ) domain containing 11	1459838_s_at	down	2,3867276
Dclk1	13175	doublecortin-like kinase 1	1446190_at	down	2,3827229
			1457954_at	down	2,3818214
			1440461_at	down	2,3817618
Mgat5	107895	mannoside acetylglucosaminyltransferase 5	1428643_at	down	2,3772736
Nrcam	319504	neuron-glia-CAM-related cell adhesion molecu	1434709_at	down	2,3727646
Ets1	23871	E26 avian leukemia oncogene 1, 5' domain	1426725_s_at	down	2,3670537
4933431K23Rik	74475	RIKEN cDNA 4933431K23 gene	1453916_at	down	2,3597276
Slco4a1	108115	solute carrier organic anion transporter family	1438160_x_at	down	2,3583894
			1440578_at	down	2,3573973
Ccrl1	252837	Chemokine (C-C motif) receptor-like 1 (Ccrl1)	1437668_at	down	2,3555496
Fam19a2	268354	family with sequence similarity 19, member A	1438007_at	down	2,3553805
Sh3rf3	237353	SH3 domain containing ring finger 3	1456946_at	down	2,354621
Dab1	13131	disabled homolog 1 (Drosophila)	1427307_a_at	down	2,351711
			1442276_at	down	2,3419228
Cap2	67252	CAP, adenylate cyclase-associated protein, 2	1450910_at	down	2,3416212
Dsp	109620	desmoplakin	1435493_at	down	2,3399804
Tnfrsf21	94185	tumor necrosis factor receptor superfamily, m	1450731_s_at	down	2,3390703
2610109H07Rik	70433	RIKEN cDNA 2610109H07 gene	1432152_at	down	2,3390553
Plscr1	22038	phospholipid scramblase 1	1453181_x_at	down	2,3365674
9430019C24Rik	77279	RIKEN cDNA 9430019C24 gene	1453458_at	down	2,3354018
Mfsd6	98682	major facilitator superfamily domain containir	1424464_s_at	down	2,3306463
Atad2	70472	ATPase family, AAA domain containing 2	1443229_at	down	2,328408
Pik3r3	18710	phosphatidylinositol 3 kinase, regulatory subu	1456482_at	down	2,3283641
9,13E+15	74564	hypothetical 9130022E09	1429344_at	down	2,3269875
Ryr3	20192	ryanodine receptor 3	1427427_at	down	2,3269699
Jup	16480	junction plakoglobin	1426873_s_at	down	2,326715
			1459256_at	down	2,3254886
Zswim6	67263	zinc finger, SWIM domain containing 6	1434967_at	down	2,323782
Adamts18	208936	a disintegrin-like and metallopeptidase (repro	1437574_at	down	2,3236983
Cytsb	432572	cytospin B	1428794_at	down	2,3193243
Scrn1	69938	secernin 1	1439500_at	down	2,3184474
Vash1	238328	vasohibin 1	1445143_at	down	2,31768
			1441494_at	down	2,3162305
Palm2	242481	paralemmin 2	1441055_at	down	2,3127651
Dsc3	13507	desmocollin 3	1434534_at	down	2,3107224
Ctnnd2 /// LOC100045979	100045979 ///	catenin (cadherin associated protein), delta 2	1422592_at	down	2,3086753
Cdh2	12558	cadherin 2	1449244_at	down	2,307371
Casp3	12367	caspase 3	1426165_a_at	down	2,3066244
Fkbp4 /// LOC100048743	100048743 ///	FK506 binding protein 4 /// similar to FK506 I	1416363_at	down	2,303621
Tcf4	21413	transcription factor 4	1416723_at	down	2,30147
Wisp1	22402	WNT1 inducible signaling pathway protein 1	1448593_at	down	2,2998898
Gpr110	77596	G protein-coupled receptor 110	1421443_at	down	2,2993371
B4galt6	56386	UDP-Gal:betaGlcNAc beta 1,4-galactosyltrans	1460329_at	down	2,2970335
Reep1	52250	receptor accessory protein 1	1433509_s_at	down	2,2955985
Slc1a1	20510	solute carrier family 1 (neuronal/epithelial hig	1448299_at	down	2,291797
Spred1	114715	sprouty protein with EVH-1 domain 1, related	1423160_at	down	2,2871258
Adamts16	271127	a disintegrin-like and metallopeptidase (repro	1439604_at	down	2,2856505
Ccrl1	252837	Chemokine (C-C motif) receptor-like 1 (Ccrl1)	1437669_x_at	down	2,2835176
Gprasp2	245607	G protein-coupled receptor associated sorting	1434073_at	down	2,2821696
Cdkn1a	12575	cyclin-dependent kinase inhibitor 1A (P21)	1421679_a_at	down	2,2799683
Hoxd9	15438	homeo box D9	1419126_at	down	2,2796304
3010003L21Rik	109163	RIKEN cDNA 3010003L21 gene	1415875_at	down	2,279397

B930068K11Rik	320622	RIKEN cDNA B930068K11 gene	1440462_at	down	2,2768042
Tnfrsf21	94185	tumor necrosis factor receptor superfamily, m	1422740_at	down	2,2724097
Zfp618	72701	zinc fingerprotein 618	1436190_at	down	2,2702627
Apcdd1	494504	adenomatosis polyposis coli down-regulated 1	1437604_x_at	down	2,2671897
			1445949_at	down	2,2644033
Drp2	13497	dystrophin related protein 2	1441224_at	down	2,263004
2900027M19Rik	72880	RIKEN cDNA 2900027M19 gene	1431703_at	down	2,2618806
Zfp618	72701	zinc fingerprotein 618	1453247_at	down	2,2607975
			1457577_at	down	2,2606409
C79607	98149	expressed sequence C79607	1457761_at	down	2,260211
Tspan2	70747	tetraspanin 2	1424567_at	down	2,2597957
Kcnk2	16526	potassium channel, subfamily K, member 2	1449158_at	down	2,259623
2900011L18Rik	77082	RIKEN cDNA 2900011L18 gene	1432757_at	down	2,2567816
Kcnj2	16518	potassium inwardly-rectifying channel, subfar	1450503_at	down	2,2537282
Fam181b	58238	family with sequence similarity 181, member	1442800_x_at	down	2,2509394
Hs3st6	328779	heparan sulfate (glucosamine) 3-O-sulfotrans	1459646_at	down	2,2497663
Tex9	21778	testis expressed gene 9	1453360_a_at	down	2,2478886
5330421C15Rik	78279	RIKEN cDNA 5330421C15 gene	1454418_at	down	2,2470753
Fzd3	14365	frizzled homolog 3 (Drosophila)	1438101_at	down	2,2465222
			1458555_at	down	2,2463646
Pdgfc	54635	platelet-derived growth factor, C polypeptide	1419123_a_at	down	2,2462776
Fam107b	66540	family with sequence similarity 107, member	1416892_s_at	down	2,2446644
Col7a1	12836	collagen, type VII, alpha 1	1419613_at	down	2,2415502
Mboat2	67216	membrane bound O-acyltransferase domain c	1425029_a_at	down	2,240537
Fryl	72313	furry homolog-like (Drosophila)	1415758_at	down	2,239192
Vipr2	22355	vasoactive intestinal peptide receptor 2	1421391_at	down	2,2387733
Prlr	19116	prolactin receptor	1425853_s_at	down	2,2348492
			1442270_at	down	2,230609
Tubb2b	73710	tubulin, beta 2B	1452679_at	down	2,2287588
Sgip1	73094	SH3-domain GRB2-like (endophilin) interactin	1425181_at	down	2,2251518
Nptx1	18164	neuronal pentraxin 1	1434877_at	down	2,223054
Actg2	11468	actin, gamma 2, smooth muscle, enteric	1422340_a_at	down	2,222816
Cdkn1a	12575	cyclin-dependent kinase inhibitor 1A (P21)	1424638_at	down	2,2193627
Ndst1	15531	N-deacetylase/N-sulfotransferase (heparan gl	1445505_at	down	2,2166483
Tcf4	21413	transcription factor 4	1434148_at	down	2,216345
Tcf4	21413	transcription factor 4	1416724_x_at	down	2,213515
Spred1	114715	sprouty protein with EVH-1 domain 1, related	1423162_s_at	down	2,212618
Pde9a	18585	phosphodiesterase 9A	1449403_at	down	2,212482
Lass6	241447	LAG1 homolog, ceramide synthase 6, mRNA (	1440246_at	down	2,2088761
5330421C15Rik	78279	RIKEN cDNA 5330421C15 gene	1457705_at	down	2,20796
Sft2d2	108735	SFT2 domain containing 2	1425026_at	down	2,206463
AU015680	552875	expressed sequence AU015680	1444674_at	down	2,201588
			1440396_at	down	2,2005863
Amot	27494	angiomotin	1454890_at	down	2,1999304
Mllt3	70122	myeloid/lymphoid or mixed-lineage leukemia	1431890_a_at	down	2,1998434
Mef2c	17260	myocyte enhancer factor 2C	1421028_a_at	down	2,1998043
Entpd1	12495	ectonucleoside triphosphate diphosphohydrola	1423326_at	down	2,1989932
Krt17	16667	keratin 17	1423227_at	down	2,196449
Cep70	68121	centrosomal protein 70	1430231_a_at	down	2,1962893
Phtf2	68770	putative homeodomain transcription factor 2	1437637_at	down	2,1955676
Tcf4	21413	transcription factor 4	1434149_at	down	2,1893492
Irf6	54139	interferon regulatory factor 6	1418301_at	down	2,1893296
Amot	27494	angiomotin	1427584_at	down	2,1882467
			1441463_at	down	2,1869457
Pttg1	30939	pituitary tumor-transforming gene 1	1419620_at	down	2,1868606
Il17rd	171463	interleukin 17 receptor D	1429893_at	down	2,1867282
Zbtb20	56490	zinc finger and BTB domain containing 20	1437065_at	down	2,185984
Slc25a24	229731	solute carrier family 25 (mitochondrial carrier	1452717_at	down	2,1816816
Tmem132c	208213	transmembrane protein 132C	1438698_at	down	2,181436
Kcnk1	16525	potassium channel, subfamily K, member 1	1448690_at	down	2,179772
			1442393_at	down	2,1753166
			1447488_at	down	2,174129
Pon2	330260	paraoxonase 2	1429019_s_at	down	2,1683497
Myo18a	360013	myosin XVIIIA	1451422_at	down	2,167098
Runx1t1	12395	Runt-related transcription factor 1; translocat	1440310_at	down	2,1662521
Fam60a	56306	family with sequence similarity 60, member A	1448126_at	down	2,164616
Nebl	74103	nebulette	1438452_at	down	2,161714
Slc25a27	74011	Solute carrier family 25, member 27 (Slc25a2	1440090_at	down	2,1569333
			1443289_at	down	2,15682
Trp63	22061	transformation related protein 63	1418158_at	down	2,156683
Casp3	12367	caspase 3	1449839_at	down	2,1556518
Dtx4	207521	deltex 4 homolog (Drosophila)	1436545_at	down	2,1541426
			1440416_at	down	2,1515746
Ednrb	13618	endothelin receptor type B	1426314_at	down	2,1513574

			1443983_at	down	2,149415
BC059841	407813	cDNA sequence BC059841	1458544_at	down	2,148392
Rnf157	217340	ring finger protein 157	1434902_at	down	2,145635
			1439734_at	down	2,144602
			1443231_at	down	2,1441805
Aspm	12316	asp (abnormal spindle)-like, microcephaly ass	1458560_at	down	2,1419883
			1460531_at	down	2,1340663
Bcl11a	14025	B-cell CLL/lymphoma 11A (zinc finger protein	1426552_a_at	down	2,1335917
Sft2d2	108735	SFT2 domain containing 2	1425027_s_at	down	2,1332662
5730507A09Rik	70638	RIKEN cDNA 5730507A09 gene	1433945_at	down	2,1329582
Tcf4	21413	transcription factor 4	1424089_a_at	down	2,1325438
Ptpn14	19250	protein tyrosine phosphatase, non-receptor ty	1436442_at	down	2,128153
Odz2	23964	odd Oz/ten-m homolog 2 (Drosophila)	1420718_at	down	2,1248674
			1421534_at	down	2,121648
Tpd52	21985	tumor protein D52	1419493_a_at	down	2,1209803
Wasf1	83767	WASP family 1	1418545_at	down	2,1189039
Nkd2	72293	naked cuticle 2 homolog (Drosophila)	1419465_at	down	2,1183846
Plscr1	22038	phospholipid scramblase 1	1429527_a_at	down	2,116541
Cacna1b	12287	calcium channel, voltage-dependent, N type,	1436602_x_at	down	2,1139188
Etl4	208618	enhancer trap locus 4	1432266_at	down	2,1132562
Tpm2	22004	tropomyosin 2, beta	1449577_x_at	down	2,1116247
D330050I23Rik	399603	RIKEN cDNA D330050I23 gene	1434301_at	down	2,1096816
Sft2d2	108735	SFT2 domain containing 2	1435142_at	down	2,1096764
Pgm2l1	70974	phosphoglucomutase 2-like 1	1438774_s_at	down	2,1096015
Rnf144b	218215	ring finger protein 144B	1425282_at	down	2,108766
Myo1e	71602	myosin IE	1428509_at	down	2,1072545
AU020094	105596	expressed sequence AU020094	1455172_at	down	2,106962
4932437C15Rik	74380	RIKEN cDNA 4932437C15 gene	1432881_at	down	2,1055777
Fam49b	223601	family with sequence similarity 49, member B	1436088_at	down	2,1031208
Egflam	268780	EGF-like, fibronectin type III and laminin G dc	1434647_at	down	2,1019144
Crybg3	224273	beta-gamma crystallin domain containing 3	1434410_at	down	2,101042
Pde7b	29863	phosphodiesterase 7B	1450213_at	down	2,101029
Sms	20603	Spermine synthase (Sms), mRNA	1434190_at	down	2,0984173
			1443042_at	down	2,095779
E230012J19Rik	319664	RIKEN cDNA E230012J19 gene	1458156_at	down	2,0936668
Shisa2	219134	shisa homolog 2 (Xenopus laevis)	1423852_at	down	2,092647
Ctxn1	330695	cortexin 1	1435083_at	down	2,0911677
			1453788_at	down	2,089681
Samd5	320825	sterile alpha motif domain containing 5	1437403_at	down	2,0894613
Fzd3	14365	frizzled homolog 3 (Drosophila)	1450135_at	down	2,08882
EG623112 /// Stmn1	16765 /// 623	predicted gene, EG623112 /// stathmin 1	1448113_at	down	2,0851233
			1438310_at	down	2,084721
Sdc1	20969	syndecan 1	1415944_at	down	2,08385
Chml	12663	choroideremia-like	1435926_at	down	2,0834217
Stmn1	16765	stathmin 1	1415849_s_at	down	2,0833745
Tns3	319939	tensin 3	1455333_at	down	2,0826979
Cdc2l6	78334	cell division cycle 2-like 6 (CDK8-like)	1455031_at	down	2,0823557
Cenpf	108000	centromere protein F	1427161_at	down	2,0815115
E430022K19Rik	320086	RIKEN cDNA E430022K19 gene	1440072_at	down	2,0806162
Clca1 /// Clca2	12722 /// 807	chloride channel calcium activated 1 /// chlori	1460259_s_at	down	2,0805821
Fam65b	193385	family with sequence similarity 65, member B	1453837_at	down	2,0801558
			1446230_at	down	2,0791948
			1437916_at	down	2,078198
Cdh13	12554	cadherin 13	1434115_at	down	2,0775642
2010011I20Rik	67017	RIKEN cDNA 2010011I20 gene	1424695_at	down	2,0774949
			1441461_at	down	2,0761251
Ccnjl	380694	cyclin J-like	1459978_x_at	down	2,0760589
			1455344_at	down	2,0744314
Sox9	20682	SRY-box containing gene 9	1424950_at	down	2,0740557
Spsb4	211949	splA/ryanodine receptor domain and SOCS bc	1451418_a_at	down	2,0733273
			1441517_at	down	2,070923
Apcdd1	494504	adenomatosis polyposis coli down-regulated 1	1454822_x_at	down	2,0703459
			1456209_x_at	down	2,069197
			1440903_at	down	2,0670555
Sostdc1	66042	sclerostin domain containing 1	1460250_at	down	2,066888
D430019H16Rik	268595	RIKEN cDNA D430019H16 gene	1455447_at	down	2,0639782
Dgkh	380921	diacylglycerol kinase, eta	1457213_a_at	down	2,062617
Slco5a1	240726	solute carrier organic anion transporter family	1439588_at	down	2,0618925
Rgnef	110596	Rho-guanine nucleotide exchange factor	1419457_at	down	2,0590491
Abhd6	66082	abhydrolase domain containing 6	1419103_a_at	down	2,055367
Ednra	13617	endothelin receptor type A	1451691_at	down	2,0545623
			1458537_at	down	2,053818
			1456661_at	down	2,0532918
LOC100048050 /// Lphn2	100048050 ///	similar to calcium-independent alpha-latrotox	1444906_at	down	2,0524287

Kctd1		106931	potassium channel tetramerisation domain co	1422293_a_at	down	2,0519454
Ednra		13617	endothelin receptor type A	1433525_at	down	2,0516608
Lrig1		16206	leucine-rich repeats and immunoglobulin-like	1434210_s_at	down	2,0493374
Tle4		21888	transducin-like enhancer of split 4, homolog c	1450853_at	down	2,0489664
Edn3		13616	endothelin 3	1441924_x_at	down	2,0481906
Hmga2		15364	high mobility group AT-hook 2	1450781_at	down	2,0472178
				1459906_at	down	2,0449865
Etl4		208618	enhancer trap locus 4	1457147_at	down	2,0438077
Agrn		11603	agrin	1443980_at	down	2,042774
Mex3a		72640	mex3 homolog A (C. elegans)	1455529_at	down	2,0420113
Otud7a		170711	OTU domain containing 7A (Otud7a), mRNA	1438286_at	down	2,040725
Camta1		100072	calmodulin binding transcription activator 1	1433971_at	down	2,039872
Alx4		11695	aristaless-like homeobox 4	1421737_at	down	2,0397582
Mreg		381269	melanoregulin	1437250_at	down	2,0386143
Fez1		235180	fasciculation and elongation protein zeta 1 (zy	1454674_at	down	2,0384018
Grhl2		252973	grainyhead-like 2 (Drosophila)	1429086_at	down	2,038247
Cep70		68121	centrosomal protein 70	1422653_at	down	2,0382106
Cyp26b1		232174	cytochrome P450, family 26, subfamily b, pol	1460011_at	down	2,0380127
Asl		109900	argininosuccinate lyase	1448350_at	down	2,037876
Cd109		235505	CD109 antigen	1436346_at	down	2,0376651
AU042950		107306	expressed sequence AU042950	1458648_at	down	2,0368447
Zfp618		72701	zinc fingerprotein 618	1455591_at	down	2,036463
Col11a1		12814	collagen, type XI, alpha 1	1418599_at	down	2,0350263
Krt15		16665	keratin 15	1422667_at	down	2,0348265
				1443253_at	down	2,0320005
Cpsf2		51786	cleavage and polyadenylation specific factor 2	1431089_at	down	2,030634
Tmem38b		52076	transmembrane protein 38B	1456544_at	down	2,0279696
Pde3a		54611	phosphodiesterase 3A, cGMP inhibited	1450284_at	down	2,024972
Fam126a		84652	family with sequence similarity 126, member	1450724_at	down	2,0242426
Fam129a		63913	family with sequence similarity 129, member	1422567_at	down	2,023488
Dpysl4		26757	dihydropyrimidinase-like 4	1418298_s_at	down	2,0221665
Rbmx		19655	RNA binding motif protein, X chromosome	1416354_at	down	2,0216236
Axin2		12006	axin2	1436845_at	down	2,0212426
Atp8a2		50769	ATPase, aminophospholipid transporter-like, c	1460294_at	down	2,0204237
Pdgfa		18590	platelet derived growth factor, alpha	1449187_at	down	2,0192535
Myo1b		17912	myosin IB	1448990_a_at	down	2,0173988
Tpm2		22004	tropomyosin 2, beta	1419738_a_at	down	2,0169108
Gjb3		14620	gap junction protein, beta 3	1416715_at	down	2,0167115
				1444620_at	down	2,0146978
Spon1		233744	spondin 1, (f-spondin) extracellular matrix pr	1424415_s_at	down	2,0140998
D5Ertd525e		52336	DNA segment, Chr 5, ERATO Doi 525, expres	1443419_at	down	2,0133772
Zfp711		245595	zinc finger protein 711	1432750_at	down	2,0131595
Megf6		230971	multiple EGF-like-domains 6	1437057_at	down	2,012785
Cd9		12527	CD9 antigen	1416066_at	down	2,0116765
2900026A02Rik		243219	RIKEN cDNA 2900026A02 gene	1429089_s_at	down	2,0106432
Fam65b		193385	family with sequence similarity 65, member B	1460555_at	down	2,0089526
Fam59a		381126	family with sequence similarity 59, member A	1456271_at	down	2,0084226
Mtch2		56428	mitochondrial carrier homolog 2 (C. elegans)	1438842_at	down	2,007356
Ankrd50		99696	ankyrin repeat domain 50	1435880_at	down	2,0073507
Fgfr4		14186	fibroblast growth factor receptor 4	1427845_at	down	2,0062437
Lemd3		380664	LEM domain containing 3 (Lemd3), mRNA	1439587_at	down	2,0062237
	100042424	100042424	PREDICTED: Mus musculus similar to TF-1 ap	1445929_at	down	2,0053694
Palld		72333	palladin, cytoskeletal associated protein	1433768_at	down	2,0045664
Nrxn2		18190	neurexin II	1435908_at	down	2,0043695
Nol11		68979	nucleolar protein 11	1439166_at	down	2,0030134
				1460117_at	down	2,0011382
